# Supplementary material for: Reversal of Osseointegration as a Novel Perspective for the Removal of Failed Dental Implants: A Review of Five Patented Methods
Source: Materials (Basel). 2021 Dec 17;14(24):7829. doi: 10.3390/ma14247829 (PMC8707383; doi:10.3390/ma14247829)
Supplement: Supplementary file 1 [file materials-14-07829-s001.zip › P1 (KR) Yonsei University, Korea, Lee English machine translation.pdf]

Europäisches Patentamt
European Patent Office
Office européen des brevets

Patent Translate

Powered by EPO and Google

Notice

This translation is machine-generated. It cannot be guaranteed that it is intelligible, accurate, complete, reliable or fit for specific purposes. Critical decisions, such as commercially relevant or financial decisions, should not be based on machine-translation output.

DESCRIPTION KR20100082188

The dental implant fixture removal device for {A Device for Removing Dental Implant Fixture}

The present invention relates to a device for a dental implant fixture removed.

Implant prosthesis that means to restore it when the loss of dental tissue, but says the artificial tooth implants. Dental implants can help to replace the roots of lost teeth, after which adhesions planted artificial tooth root made of titanium without rejection in the human body in the alveolar tooth drained by fixing an artificial tooth to restore the original function of the teeth a state-of-the-art treatment. If the dental implant does not have a big problem if you complete all at once, if you then implant fixture placement inappropriate for prosthetic restorations or change your treatment plan, you need to remove the dental implant fixture for the implant to the existing If the arises. If this is the case, typically pine tree version (Trephine bur), Burr Round (Round bur) or blood Shure server (Fissure bur), which is mainly used to implant removal and implant fixture. However, if you are using these devices requires extensive surgery with the surrounding bone and after surgery also results in a difficult placement of large bone defects blossomed added a new implant, as well as mounting of removable dentures. The naemeurosseo out by combining the extraction tool into the separation device for the implant (implant) so that you can easily remove the placement of the implant fixture (Republic of Korea Patent No. 721 603 No.) or implant fixture engaging part implant fixture using the current lever principle this technology was developed to separate from the bone (Republic of Korea utility model No. 414 573). However, problems caused large defect parts of each corrosion body surrounding the case of removing dental implants sheet metal corrosion body as described above, and do not address, the need for equipment to solve this problem has emerged. Reference throughout this specification has a number of patents and publications are cited that are displayed. The disclosure of these patents and publications cited contents incorporated by reference herein as to more fully describe this invention and the state of the art to which this invention pertains.

The present invention provides a dental implant fixture for removal device which can ensure the stability of the implant procedure and improve the ease of the procedure. In particular, the present invention provides a dental implant fixture removal device for minimizing bone parts when removing the metal sheet corrosion body dental implant using a partially deficient golgoe reaction by the high temperature applied to the predetermined time of implantation the implant fixture to a suitable temperature. The present invention also simplifies the procedure to remove the implant the implant fixture and the dental implant fixture removal device provides for excellent stability. Other objects and advantages of the present invention will become apparent from the following detailed description, claims and drawings. According to one aspect of the invention there is provided a heater unit for heating the implant fixture (a); (B) a temperature sensor portion for sensing a temperature of the implant fixture; And (c) to provide a dental implant fixture removal device comprising a control unit for controlling the temperature of the heater by the temperature sensed by the temperature sensor. As used herein, the term "implant fixture" means an artificial tooth root which is used in the dental implant operation. The implant fixture may preferably be made of a metal or non-metal, it is made of a metal and more preferably. Most preferably is a titanium (Titanium) of a material. According to embodiments of the present invention, the unit characterized in that the heater has a shape capable of contacting with the inner side of the implant fixture. According to embodiments of the present invention, it is characterized in that it comprises a heat insulating member that can block the transfer of heat to the outside from the temperature sensor unit. According to embodiments of the present invention, the control unit and when heated to a preset temperature to the implant fixture, which is more than a predetermined time characterized in that parts of the heater-off (off). According to the embodiment, the preset temperature is characterized in that the 40-60 ° C. According to the embodiment, the predetermined time is characterized in that the time point at which the surrounding bone necrosis of the implant fixture after reaching the preset temperature.

As used herein, the term "necrosis (osteronecrosis)" means a physical necrosis of bone cells, this cellular tissue breakdown caused by heat or external function is stopped. According to the embodiment, the case wherein the device further includes an alarm to inform an alarm signal, and reaches the preset temperature, or time value, characterized in that the alarm control to express additional alarm signals. According to embodiments of the present invention, wherein the device further comprises a

user input, the control unit is characterized in that parts of the heater control based on the temperature or time values input through the user input. A summary of the features and advantages of the present invention as follows: (i) The invention according to the heater unit to the device for dental implant fixture removed, heating the implant fixture (a); (B) a temperature sensor portion for sensing a temperature of the implant fixture; And (c) a dental implant fixture removal device comprising a controller to control the heater unit temperature by the temperature sensed by the temperature sensor unit. (Ⅱ) the present invention is not suitable for prosthetic restorations after implant or can advantageously minimizes a implant fixture removed during bone defects implanted due to the treatment plan changes, and simplify the removal procedure. (iii) the present invention can be separated to be applied to the implant fixture of the various types equipped with a heater, and, by using a heater for the implant fixture exhibit exceptional thermal conductivity and energy efficiency. (iv) the present invention can be removed safely and quickly alert the added implant fixture implanted in indicating the temperature and the time.

Or less with reference to the accompanying drawings, described in detail a preferred embodiment of the present invention, but the present invention is not limited or restricted by the embodiments. In the following description, specific details for the well-known functions or constructions may be omitted to clarify the gist of the present invention. 1 is a view showing the configuration of the dental implant fixture removal device in accordance with the invention. The device is composed of a control unit for controlling the temperature of the heater by the temperature sensed by the temperature sensor and temperature sensor that can detect the temperature of the heater unit, fixture to heat the fixture. 2 is a cross-sectional view in the state coupled with the heater unit and the temperature sensor unit Miniature fix dental implant of the present invention. Figure 3 to 6 are diagrams showing the process by sequentially operating the heater unit and the temperature sensor portion of the present invention to remove the dental implant fixture. Hereinafter will be described as a device for dental implant fixture removed through the process of repairing or removing the prosthesis unsuitable for a dental implant fixture implanted due to the treatment plan such as change. In the heater unit structure, the external forms may typically have a cylindrical shape, may be shaped in a variety of shapes that correspond to the internal structure of the implant fixture. Characterized in that said heater unit has a form that can be the implant inside the point, line or surface contact. May consist of the inner heater unit is self-heating material, the thermal conductivity may include a high heat generating element, the inside and the outside are separated may include a high thermal conductivity material. The temperature of the heater may dissipate heat of at least 40-1000 ° C. Way that the heat dissipation in the heater portion is outside, it is possible to radiate directly or indirectly. Through the method and the medium for transferring heat by direct contact with the heater unit and the implant fixture can have a way to transfer heat. The medium comprises a liquid, gas, or solid. Passing the said heat can be variously changed according to conditions and design specifications.

The heater unit can be a device or a temperature sensor and detachable. Connections of the heater unit and the heater unit and the temperature sensor device or the part may be variously changed according to conditions and design specifications. If you remove the dental implant fixture after first inserting a heater inside the heater fixture causes a fever. At this time, in order to improve the thermal conductivity and the energy efficiency of the heater and the part and the fixture body it can be added to the medium inside the fixture. Preferably, the matrix body may be used for liquid, gas or solid, and more preferably may use a liquid or gas, can be most preferably used for the liquid. The liquid medium is typically used for the body means a non-toxic liquid harmless to the human body for use in dental procedures. The temperature sensor portion includes a temperature sensor that can detect the temperature of the fixture, and a heat insulating member to block the heat of the heat and the external temperature sensor. A temperature sensor unit is integrated with the separation device and the heater unit that is configured to operatively connect between (operatively linked) may be configured as external to operate. The temperature sensor can be configured in a manner to sense the infrared rays is possible to detect the temperature of the fixture in an analog or digital manner, the blood contacting the contact water and the direct sensor system for sensing the temperature of the radiation in the contacted water . For example, contacting the sensor directly to the dental implant fixture passes the electrical signal to the controller or by sensing the temperature senses the dental implant fixture, or infrared radiation from the surrounding gum delivers an electric signal to the control unit. The heat insulating member may be composed of a material that can block the normally open, if clinically proven safe and material, it is possible either to. The heat insulating member may be of a shape is surrounding the temperature sensor in order to block the heat from the temperature sensor to the outside. The control unit may be included in the handpiece, is separated can be configured independently. The control unit is characterized in that for controlling the implant fixture and heated to a preset temperature, by a heater-off (off) when a certain time or more.

The preset temperature of 40-60 ° C, more preferably 45-50 ° C, most preferably 46-47 ° C which means that. The predetermined time means that the surrounding bone of the fixture and then heated to the preset temperature of the implant fixture when the necrosis has occurred. If the device that further includes an alarm to inform an alarm signal and, based on the set temperature is reached or a time value, characterized in that the alarm control to express additional alarm signals. Alarm signal is expressed by sound, light or vibration, and the user on the basis of this warning signal may determine the use of the implant device. The device further comprises a user input, the control unit is characterized in that parts of the heater control based on the temperature or time values input through the user input. Hereinafter will be described the operational structure of a dental surgery apparatus in accordance with the invention. 7 is a block diagram illustrating the structure of the operation of dental surgery apparatus in accordance with the invention. In addition, as for the above-described configuration and the same, and many of the same and have the same or substantial same reference numerals, and detailed description thereof will be omitted. As shown in Figure 7, the operator sets the temperature and time via the input unit, and thereby the heat generating portion of the heater device for the dental implant fixture by removing the rear heater fix the implant insertion inside the Miniature. In this case, inserting the parts of the heater before the addition of a medium inside the fixture body can increase the thermal conductivity and thermal efficiency. When temperature is reached, or the time the temperature of the dental implant

fixture has a predetermined sends a notification signal to alert the operator finally sending part electrical signal. Operator received the notification signal may terminate the procedure by measuring the state of the gum around the implant fixture to the naked eye, or determine whether additional treatment. Hayeotneun described a preferred embodiment of the present invention in detail by bar, this specific technique according to a person of ordinary skill in the art is only for a preferred implementation example, and this point is not to be limited in scope the invention is obvious.

Accordingly, the scope of this invention is to be determined by appended claims and their equivalents.
