# Supplementary material for: Reversal of Osseointegration as a Novel Perspective for the Removal of Failed Dental Implants: A Review of Five Patented Methods
Source: Materials (Basel). 2021 Dec 17;14(24):7829. doi: 10.3390/ma14247829 (PMC8707383; doi:10.3390/ma14247829)
Supplement: Supplementary file 1 [file materials-14-07829-s001.zip › P2 (US) Bern University, Switzerland, Braegger.pdf]

US 20130071813A1

(19) **United States**

(12) **Patent Application Publication**

Braegger et al.

(10) **Pub. No.: US 2013/0071813 A1**

(43) **Pub. Date: Mar. 21, 2013**

(54) **DEVICE FOR DETACHMENT AND EXPLANTATION OF BONE IMPLANTS**

(75) Inventors: **Urs Braegger**, Neuenegg (CH); **Michel Gigandet**, Worb (CH); **Jan Vizek**, Bern (CH); **Ulrich Hegnauer**, Ruedtligen (CH)

(73) Assignee: **UNIVERSITAET BERN**, Bern (CH)

(21) Appl. No.: **13/699,329**

(22) PCT Filed: **May 9, 2011**

(86) PCT No.: **PCT/EP2011/002284**

§ 371 (c)(1), (2), (4) Date: **Nov. 21, 2012**

(30) **Foreign Application Priority Data**

May 21, 2010 (EP) 10405104.0

Publication Classification

(51) **Int. Cl.**
A61C 8/00 (2006.01)

(52) **U.S. Cl.**
CPC *A61C 8/0089* (2013.01)
USPC **433/173**

(57) **ABSTRACT**

A device for detachment and explantation of bone implants, in particular tooth implants, from their connection to the bone tissue comprises a unit having an elongated handle (**10**), a cooling module (**7**), a vibration module (**8**) for generating mechanical vibrations, and a grip head (**6**) which is secured to the handle (**10**) and which has a seat (**14**) for the bone implant to be explanted, in particular an implant head of the bone implant, or for an adapter piece for gripping the bone implant. The grip head (**6**) is coupled to the cooling module (**7**) and to the vibration module (**8**) in such a way that it is coolable to a desired temperature by means of the cooling module (**7**), and the vibrations of the vibration module (**8**) are transferable into the grip head and further to the bone implant or the adapter piece. The seat in the grip head (**6**) has at least one clamping element (**12-15**) which is able to exert radial clamping forces, with respect to a rotational axis, on the bone implant or adapter piece held in the seat (**14**). The rotational axis is situated at an angle, preferably at essentially right angles, with respect to a longitudinal axis of the handle (**10**). The grip head (**6**) is also coupled to the handle (**10**) in such a way that a manually applied rotational motion of the handle (**10**) about the rotational axis is transferable to the bone implant or the adapter piece. This device allows a bone implant to be explanted easily, quickly, and safely, without damage to tissue.

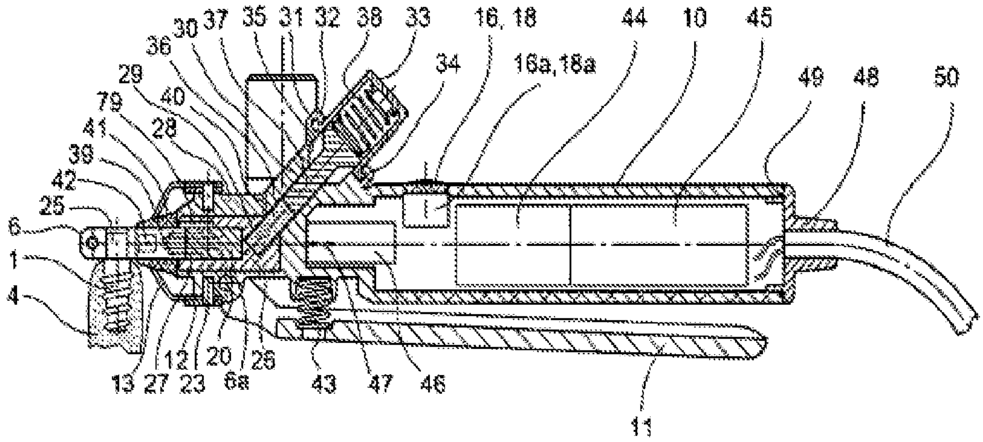

Fig. 1

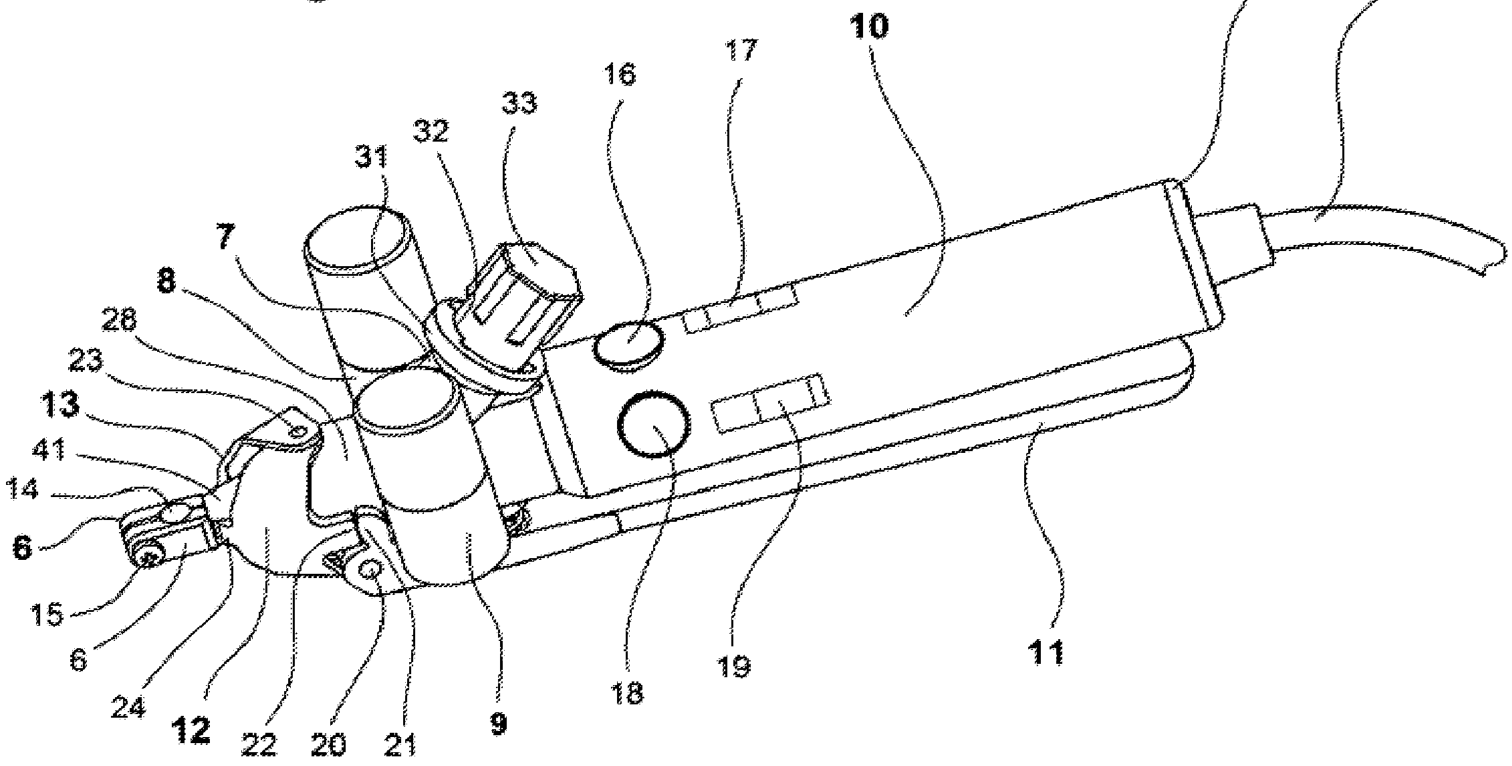

Fig. 2
(A-A)

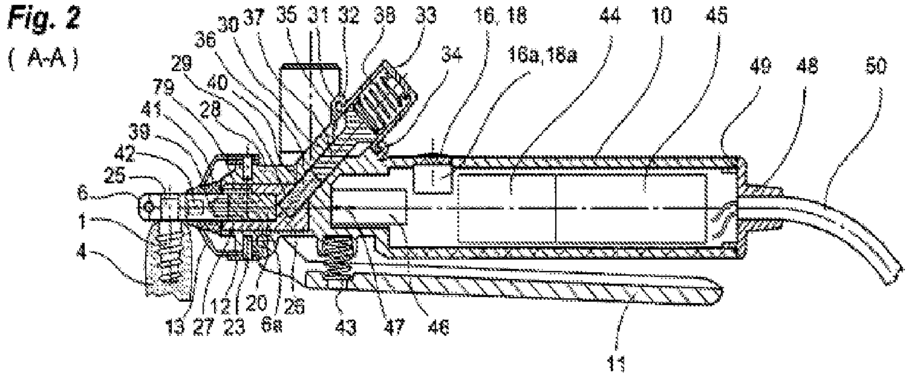
37
35
31
32
38
33
16, 18
44
10
45
36
30
34
16a,18a
49
48
50
29
79
40
28
41
39
42
25
6
1
4
13
27
23
12
20
26
6a
43
47
46
11

Fig. 3

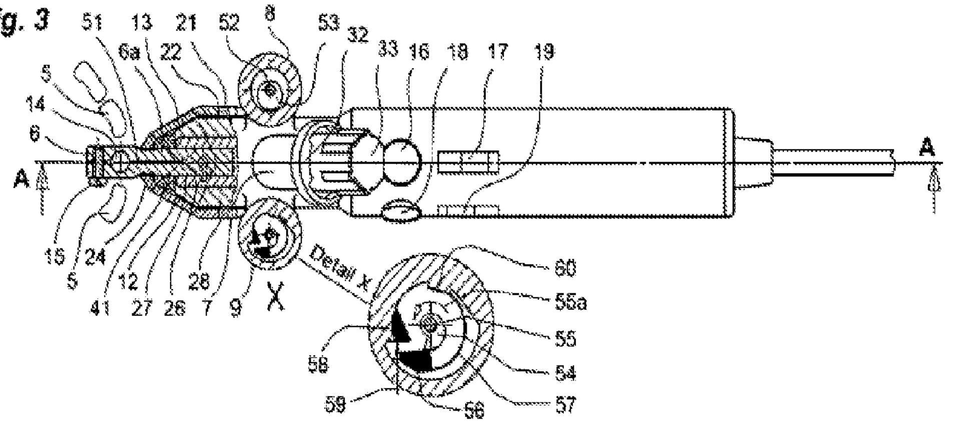
51
13
21
52
8
53
32
33
16
18
17
19
6a
22
5
14
6
A
A
15
24
5
12
28
41
27
26
7
9
X
Detail X
60
55a
55
54
57
58
59
56

Fig. 4

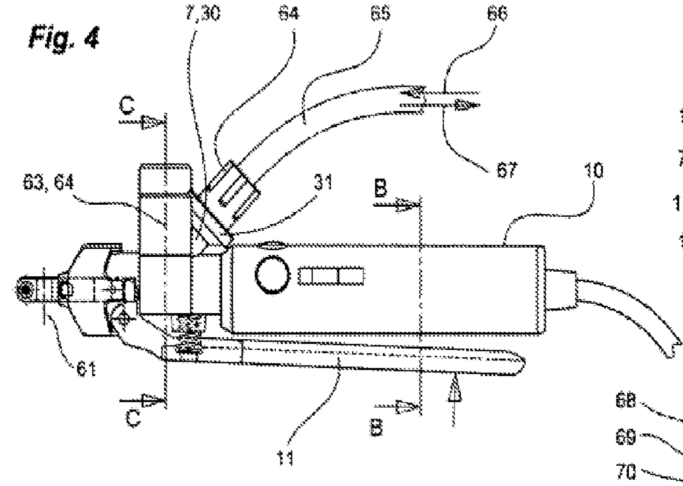

Fig. 5
(B-B)

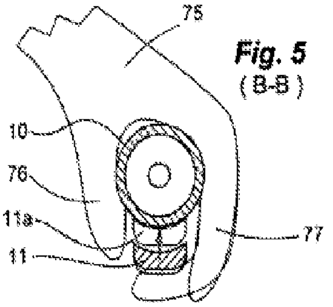

Fig. 6
(C-C)

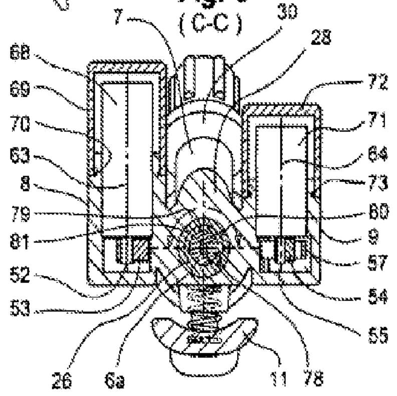

Fig. 7

Fig. 7b

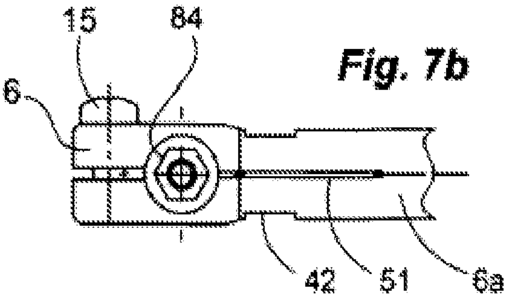

Fig. 7c (D-D)

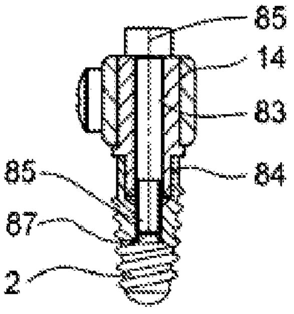

Fig. 7a

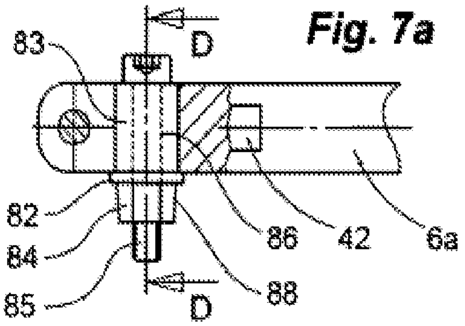

Fig. 8

Fig. 8b

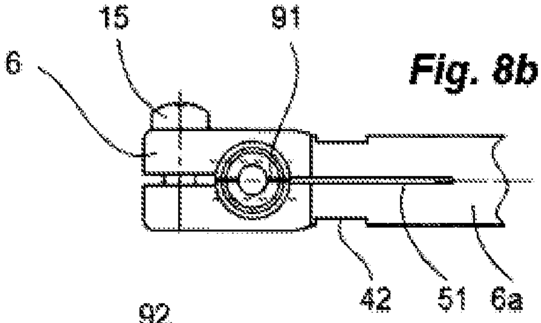

Fig. 8c (E-E)

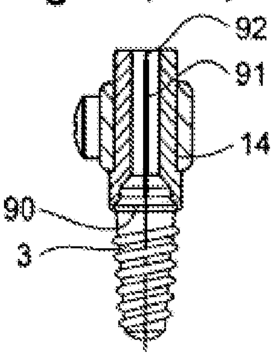

Fig. 8a

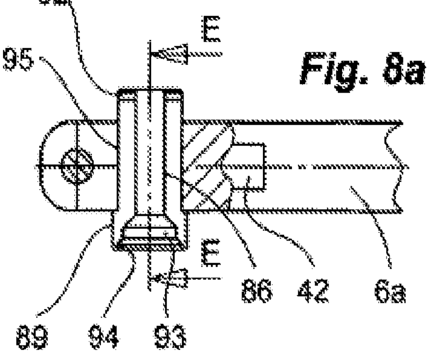

DEVICE FOR DETACHMENT AND EXPLANTATION OF BONE IMPLANTS

TECHNICAL FIELD

[0001] The invention relates to a device for detachment and explantation of bone implants, in particular dental implants, from their connection to the bone tissue.

PRIOR ART

[0002] Dental implants have been successfully placed in bone tissue for the past 30 years. In general, dental implants take over, support, or replace the functional, static, and mechanical tasks of tooth roots for anchoring the dental prosthesis. Such implants do not achieve their full load capacity and functionality until they have completely bonded with the surrounding bone substance. Depending on the clinical situation, implants are subjected to load, i.e., joined to the prosthetic reconstruction, immediately after surgery, either early (within 2-8 weeks) or with a conventional delay (healing period of greater than 2 months).

[0003] In Switzerland, for example, approximately 100, 000 dental implants are installed per year (source: Swiss Implant Foundation). Using well-documented implant systems and established clinical concepts, at the present time it may be expected that 98-99% of installed implants will heal in the tissue. However, studies have shown that over a period of 5 years, 2-5%, i.e., 2000-5000 out of 100,000 implants, and over a period of 10 years, approximately 5-8%, i.e., 5000-8000 out of 100,000 implants, fail. In 2007, the penetration rate of prosthetic implant care in Europe was only 3-5% of the prosthetic cases.

[0004] The success, demand, and patient expectations may disproportionately increase the penetration rate. Unfortunately, after approximately 10 years, depending on the study, 6.6% to 14% of patients have excessive bone atrophy due to peri-implantitis.

[0005] Prophylactic measures may have a long-term benefit in minimizing this rate. However, if an implant is allowed to undergo further peri-implantitis without treatment, the surrounding bone is resorbed; in the worst case, fracture of the jaw has even been reported. In addition, there are groups of patients who are at increased risk due to general medical reasons or because of smoking.

[0006] From the current perspective, and not just for dental implants, on average much greater than 10% of all installed bone implants unexpectedly fail. For dental implants, in recent years research and development have focused, among other things, on qualitative improvement and acceleration of the adhesion between the implant surfaces and the bone. The results of successful animal testing with increasing unscrewing torque are directly measurable. Thus, any improvement in the surface adhesion is inversely related to corresponding increasing difficulty in performing a necessary explantation, for various reasons, without damage to the surrounding tissue. At the present time, surgical explantations are generally very delicate, high-risk procedures which entail high costs, risks, and complications in follow-up treatment. Frequently, as the result of an explantation it may no longer be possible to reinsert a new implant, and the patient may be in a much worse state than before the procedure itself. As a rule, the attending dental practitioner is confronted with unforeseeable difficulties, and without suitable instruments and methods, inadvertently damages the surrounding bone tissue.

[0007] For the required explantation of dental implants, to date there is no known or practicable system or method which allows simple, rapid, and safe explantation without damage to tissue. Methods and procedures are certainly available for explantation of an implant which are medically proper and acceptable. However, the possible severe damage and consequential damage and the resulting problems are clinically documented, and are described to some extent in the present document. In summary, the corresponding techniques are referred to in the present document as "hard explantation." The direct converse is "soft explantation," which in a novel and advantageous manner allows gentle attachment of the anchoring of the implant in bone tissue by using the novel explantation device according to the invention.

[0008] First of all, one self-evident option is to unscrew the implant. However, for dental implants the unscrewing torque is of key importance. Stated in simple terms, the higher this value and the more quickly high values are achieved, the better and more quickly the surface of the implant bonds to the bone tissue. Therefore, high unscrewing torques are desirable. In animal testing, the explantation torques of osseointegrated implants reach 1.485 Nm after 2 weeks, 1.709 Nm after 4 weeks, and 1.345 Nm after 8 weeks, and in the so-called hydrophilic SLactive implant design, are grown in 8-21% more securely than hydrophobic SLA implants.

[0009] Of course, the extreme unscrewing torques in the range of 100 to 200 Ncm may be determined only in animal testing, under laboratory conditions and with destruction of the animals. In practice, it is difficult to use the unscrewing method on account of the space constraints during the procedure, the difficulties in grasping the various shapes of the implant head, etc. The mounting piece, the neck of the implant, or even the surrounding bone segment may fracture, with inability to define the rupture site.

[0010] If an implant which is not yet completely healed is exposed to a torque too early (for example, in the fastening of prosthetic secondary parts), the implant rotates. This is perceived and reported by the patient as pain. These situations are referred to as so-called "spinners." It is still possible for the implant to heal after 2-3 months if it is not further rotated or treated.

[0011] For so-called mini-implants, which are used in orthodontics as a fixed point for applying forces for tooth movement, after the tooth movements are completed the miniscrews may be easily removed using wrenches and ratchets. For dental implants, unscrewing is successful only when the implant has apically integrated only a few millimeters. For this reason, when there are bone defects which cannot be successfully treated, implants are also often left in until the body itself rejects the implant, i.e., until the inflammatory processes have progressed far past the apical location, and the implant may be easily unscrewed, or it spontaneously exfoliates. This results in a bone defect which becomes increasingly larger, with the disadvantages mentioned above.

[0012] As described above, years of effort by implant manufacturers have measurably qualitatively and quantitatively increased and accelerated the adhesion of the surfaces between the implant and the bone substance.

[0013] For the above reasons, only the hard explantation method of milling is currently used in clinical practice. According to the current state of the art, for implant failures, for example peri-implantitis, implant fracture, etc., or for intentional explantations, for example after an orthodontic treatment involving a gum implant, dental implants are

removed in multiple steps using a concave cutter, or are extracted from the bone in a circular manner using a thin milling cutter. The appropriate hollow-tube cutter which fits the particular dental implant diameter is offered in a product range by most dental implant manufacturers. Following the milling operation, the remaining composite of the implant with the bone may have to be unscrewed or luxated from the bone, which is possible only with significant application of force using forceps or other extraction instruments. Naturally, this type of explantation results in significant bone defects, which have considerable adverse effects for possible subsequent implantations. For example, for implants whose upper part is expanded in the shape of a collar, if this topmost part is removed first using a diamond cutter (so that the tubular cutter may be guided as closely as possible along the implant), high frictional temperatures result which, in the most unfavorable case, lead to denaturing of the tissue surrounding the implants. The site remaining after the explantation is often damaged, even after a considerable period of time, so that the bone regeneration or healing may not be successful since the walls are reduced too greatly to provide a satisfactory base for regeneration. If an implant should be subsequently placed at the same site, significant bone augmentation is first necessary. In confined spaces such as in the lower front teeth region, there is a further risk of the concave cutter injuring the roots of the adjacent teeth, which may even result in loss of a healthy neighboring tooth. In addition, the titanium particles generated in the milling process are absorbed in the body, where they remain in the tissue.

[0014] Another disadvantage of milling which is difficult to subsequently remedy lies in the tool itself. Removal of the implant from the bone can occur only with the sacrifice (i.e., milling out) of the bone mass which is cut away using the cutting tool. This drawback, in particular the loss of the cross-sectional volume, may be compensated for in very few cases by using an implant having a larger diameter. However, this is not possible until after the surgical injuries have healed after a period of time which cannot be precisely determined in advance. Usually, an attempt must be made to replace the milled-out cross-sectional mass, using methods not described here, in order to achieve the same static load capacity. Here as well, the period of time between the explantation and a new approach which meets the expectations of the patient is difficult to determine in advance, although it must be noted that this always takes several months. During this time, the patient is generally directed to have temporary restorations, which are not inexpensive, and which are unusable after a possible reimplantation.

[0015] Piezosurgery instruments also exist for the explantation. Some case reports demonstrate the advantages of this method over concave milling. However, in this method as well, the bone surrounding the implant must be removed. In the process, at least as much volume is removed as the space occupied by the tool.

[0016] If the hard explantation is dispensed with, the patient with the implant is left with chronic inflammation with suppuration from the soft tissue. The chronic inflammation results in a slowly progressing peri-implantive bone defect. Ultimately, a situation similar to that after milling explantation is observed.

[0017] To a certain extent, attempts have also been made to remove the implant from the tissue by introduction of cold. However, one problem is that not only implants used in dentistry, but also other bone implants are often made of a material, preferably ceramic, which does not conduct cold.

[0018] It is known from the processing of histological sections that biopsies containing osseointegrated metal components may be removed from surrounding bone using the freeze fracturing technique. The samples are flash-frozen at −196° C. and placed in interim storage at −80° C. A fracture forms at the interface between the metal surface and the surrounding bone (Donath, K. (1988), The cutting/thin-ground technique for producing histological preparations of non-cuttable tissues and materials [English translation of title], Der Präparator 34: 197-206; Berglundh, T. & Lindhe, J. (1997), Healing around implants placed in bone defects treated with Bio-Oss®: An experimental study in the dog, Clinical Oral Implants Research 8: 117-124). Small residues of organic substances or bone islands sometimes adhere to the implant surface. However, the majority of the surrounding bone may be removed without damage. Salmassy & Pogrel (Salmassy, D A & Pogrel, M A (1995), Liquid nitrogen cryosurgery and immediate bone grafting in the management of aggressive primary jaw lesions, Journal of Oral and Maxillofacial Surgery 53: 784-790) have shown that under the influence of cold, bone lesions heal with less risk, and therefore a new implantation may be performed more quickly. Since tooth roots together with periodontal tissues may also be present in the surroundings of the implants to be removed, the cold shock must not exert a necrotizing destructive effect on this tissue. In animal testing, cryoprobes at −81° C. were applied to the buccal bone of tooth roots, which was frozen and thawed three times. Histological samples were analyzed after 1 hour, 48 hours, and 30 days. After 30 days the histological profile returned to normal, with no observable resorption or ankylosis. The frozen lesion made a recovery (Tal, H., Kozlovsky, A., Pitaru, S. (1991), Healing of sites within the dog periodontal ligament after application of cold to the periodontal attachment apparatus, Journal of Clinical Periodontology 18: 543-547).

[0019] The cold shock technique is also used in the treatment of tumors in bone tissue. On the one hand, the tumor tissue may be radically removed in the vicinity of surgical probes which are brought to very low temperatures using liquid nitrogen, for example, but on the other hand, bone transplants and subsequent implants may be successfully installed again into the healing bone lesions (Salmassy & Pogrel 1995, see above).

[0020] Ultra high-frequency electrosurgery has previously been tested in some patients for removal of osseointegrated implants. The implant to be removed was contacted by the surgical loop for three seconds at 27 MHz, with no local anesthesia. After two weeks the implants were unscrewed. The authors reported necrosis which was limited to the extension.

[0021] Heat necroses are generally considered to be poorly healing wounds. Risks and side effects have not been studied, and since 2004 there have been very few publications regarding the method (Massei, G. & Szmukler-Moncler, S. (2004), Thermo-explantation: A novel approach to remove osseointegrated implants, European Cells and Materials 7 (Suppl. 2): 48).

[0022] The prevention of heat generation plays an important role in the placement of implants. For this reason, the preparations for the implant tunnels are made using slowly rotating, efficiently cutting pilot drills and twist drills. The

entire preparation takes place without pressure, with continuous irrigation with cooled physiological saline solution.

[0023] If implant components must be ground down for prosthetic reasons, once again intensive cooling during the preparation is recommended. The thermal conductivity of titanium allows massive heat increases deep into the tissue if there is no (water) cooling (spray, air). Within seconds, temperatures are reached which may result in denaturing of protein and heat necrosis (Bragger, U., Wermuth, W., Török, E. (1995), Heat generated during preparation of titanium implants of the ITI® Dental Implant system: An in vitro study, Clinical Oral Implants Research 6: 254-259).

[0024] Theoretically, another option for removing implants from the bone is to detach the implant from the tissue using a lower-frequency vibration than in the above-mentioned method (UHF electrosurgery).

[0025] This is described, for example, in WO 88/02246 (C. A. Dicecca, F. G. Heller), using a device which by means of back-and-forth pulses of a relatively large, pneumatically driven piston is designed to detach and knock out bone implants from their connection with the bone mass.

[0026] WO 2006/020803 A2 (John Hopkins University) likewise discloses a device for extracting implants, which includes a generator for repetitive mechanical force, and which may also have a heat transfer mechanism for controlling the temperature of the implant. The mechanical forces are transmitted to the implant via a rod.

[0027] The often underestimated clamping effect of the bone when the bone mass grows onto the surface of the implant results in very high strength of the connection between the bone and the implant. The powerful pulsing in the manner of a jackhammer is therefore not suitable for the brittle, porous bone mass, which has insufficient volume. It is not apparent from the cited publications how a bone implant may be gripped and extracted. There are no known clinical applications of the described device.

[0028] For the required explantation of dental implants, or any bone implant, to date there is no system, device, or generally accepted method which allows simple and safe explantation without damage to tissue. The explantation procedures are currently performed without applicable standards, and it has been shown that in the design, too little attention has been paid to the expected and statistically proven failures. The primary focus has been on the methods and devices for implantation; explantation has instead been regarded as unlikely and accorded little attention to date.

DESCRIPTION OF THE INVENTION

[0029] The object of the invention is to provide a device which is part of the technical field stated at the outset, and which allows simple, rapid, and safe explantation of a bone implant without damage to tissue.

[0030] The achievement of the object is defined by the features of claim **1**. According to the invention, the device comprises a unit having

[0031] a) an elongated handle;

[0032] b) a cooling module;

[0033] c) a vibration module for generating mechanical vibrations;

[0034] d) a grip head which is secured to the handle and which has a seat for the bone implant to be explanted, in particular an implant head of the bone implant, or for an adapter piece for gripping the bone implant;

[0035] wherein

[0036] e) the grip head is coupled to the cooling module and to the vibration module in such a way that it is coolable to a desired temperature by means of the cooling module, and the vibrations of the vibration module are transferable into the grip head and further to the bone implant or the adapter piece;

[0037] f) the seat in the grip head has at least one clamping element which is able to exert radial clamping forces, with respect to a rotational axis, on the bone implant or adapter piece held in the seat;

[0038] g) the rotational axis is situated at an angle, preferably at essentially right angles, with respect to a longitudinal axis of the handle; and wherein

[0039] h) the grip head is coupled to the handle in such a way that a manually applied rotational motion of the handle about the rotational axis is transferable to the bone implant or the adapter piece.

[0040] In the future, gentle and simple explantation must be established as a general quality standard (Yeakley, B. & Goswami, T. (2009), Orthopedic Implant Retrieval-Imperatives and Possibilities, Annals of Biomedical Engineering 37: 2326-2336). Explantations must therefore generally be carried out in a manner that is predictable, gentle, flexible, and independent of the planned life expectancy of an implant. The new generation of dental implants does not always accept explantation as a failure; on the contrary, very rapid re-implantation will make the development of new treatment methods possible in the future. Successful innovations often make it difficult to simultaneously address the successful management of the already expected statistical failures, for which there is often a willingness to remain silent. Optimal methods and devices will be of paramount importance in the future, not only for the use of implants and improved connections with the bone tissue, but also for simple, gentle explantation. The new generation of all bone implants should be designed from the outset in such a way that the use of an explantation tool and the connection to an explantation device are flawless and as precise as possible. It is also conceivable that new implants as delivered may include an explantation tool part for docking to an explantation device. An analogous situation is found in the aviation industry, which uses a removal and disassembly tool that exactly fits the heads of the particular screws or other elements to allow the screws or elements to be repeatedly screwed and unscrewed at the appropriate part of the aircraft with the aid of the appropriate devices, with application of the precisely prescribed forces, in order to perform specified periodic service.

[0041] The device according to the invention allows an analogous procedure, in that the application of force between the head of the implant which is made accessible is possible in a twist-resistant manner with respect to the handle, using a wrench attachment. With the aid of a handle and using the appropriate force, an attempt may then be made to detach the connection between the implant and the bone tissue in the direction opposite the screwing direction. The seat of the grip head is advantageously essentially cylindrical, the rotational axis corresponding to the cylindrical axis. This allows the accommodation of common implant heads as well as connecting parts of adapter pieces having a matching shape. The cylindrical shape also allows transmission of force over a large surface, and thus, a secure hold of the accommodated element under various forces.

[0042] To obtain a compact and ergonomic tool, the grip head is advantageously secured directly to a distal end of the handle. The angled configuration between the rotational axis and the longitudinal axis of the handle then results primarily from the orientation of the seat in the grip head.

[0043] Due to its design, handling, and combination of detachment steps in the explantation, the novel explantation device may be used on most bone implants. The reason is that the majority of bone implants are made primarily of metal, preferably titanium or titanium alloys. The bone implants are cylindrical or slightly conical, and as a rule are provided with an external thread for screwing into a hole previously made in the bone. The bone implants vary only in their size, diameter, length, and in particular head shape, which is decisive for the basic function of the implant. Thus, when the explantation device is used, the same physical, mechanical, and biological basic conditions logically apply for all bone implants. Of course, for application in a particular medical field, the dimensions of the device, the magnitude and quality of the variables used, the shape and size of the grip head and of the tool elements, the space conditions, and the positioning of the implant to be explanted in the body or the lower or upper jaw, as well as the procedural options of the surgeon are taken into consideration. This cautious and finely differentiated procedure is particularly important for explantation from the upper jaw, or from bone parts which have become brittle due to the aging process and have become increasingly porous in structure, and which do not tolerate inordinately large mechanical effects.

[0044] In principle, and for success of the device according to the invention, it is necessary for the implant head to be gripped firmly and in a positive-fit manner so that coupled cold and mechanical energy may act without losses. For the currently used implants the exact head dimensions are known, whereas for the bone implants which have been in use for years, their shape and exact dimensions are frequently unknown. After exposing the implant head with the aid of the tool elements of the novel device, the surgeon himself may perform the necessary shaping and adapting of the gripping elements within a reasonable period of time.

[0045] Although the present patent specification addresses the explantation of dental implants in detail and describes a corresponding device as well as embodiment variants thereof, other bone implants may be explanted using appropriately modified docking tool elements and an adapted geometry of the device.

[0046] The novel device is characterized in that that it is designed as a compact and easily handled unit having a grip head and a cooling module, whereby the latter, with a suitable coolant, is able to cool the bone implant until the desired detachment effect due to the cold or the initial weakening of the connection between the implant and the bone tissue occurs. The cooling causes the structure of the adjacent bone tissue to change in such a way that radial tension force develops on the implant. The grip head, and thus the implant held therein or the adapter for holding the implant, are cooled to the desired temperature by the cooling module, and the cold is conducted into the implant over a period of time that is determined to be optimally effective. Fixed docking of the grip head tool to the dental implant is advantageously provided in such a way that the supplied cooling energy cannot damage the surrounding tissue.

[0047] In dentistry, so-called "CO_2 snow" (dry ice) is preferably used, which is provided from an external pressurized gas container into a collection container via an outlet nozzle, and is then compressed into an iced body which is shaped to fit in a receptacle in the cooling module. The iced body may be inserted into the cooling module, where the cold is then introduced into the grip head. Instead of a CO_2 iced body, a cooling element composed of some other material having sufficient cold storage capacity, for example, which is externally cooled in advance, may also be inserted into the cooling module. Alternatively, it is conceivable for cold to be generated directly from a pressurized CO_2 vial which is dockable in the handle.

[0048] Alternatively, the cooling may be achieved by circulating a suitable cooling medium. Using such a device, for specially designed implants it is likewise very simple to employ a cooling effect and a heating effect in any desired alternation for removing the surface; i.e., the contraction method and expansion method may be applied using the same device.

[0049] In the cooling chamber, also outside the device, liquid cooled to minus 30°-40° may be generated and filled into the cooling chamber through an appropriate line, and after the delta in cooling energy is delivered to the thermal jackets, the liquid is returned through the outlet opening to the cooling unit, in which it is once more lowered to the minus temperature in a circuit and led to the thermal chamber. This supplied cold energy is controllable at all times by measurement upon entry and exit and by transfer to the connecting piece and to the grip head, and may be interrupted or shut off completely as needed.

[0050] The thermal chamber, which has the same design with regard to shape as the flowthrough method, provides another option for generating the cold energy. In this variant, a thermal block in the shape of multiple "pineapple rings" stacked one on top of the other is precooled to the desired minus temperature (preferably to minus 50°-70° C.) in a freezer. The entire block is made of a material that is able to absorb sufficient cold energy, and when inserted into the cooling chamber, to appropriately deliver the preloaded cold energy to the connecting piece and further to the grip head. In this variant the temperature difference, and thus, also the period of time over which cooling may take place, is already known. However, in this method the procedure cannot be regulated or terminated as easily as for the flowthrough variant. For the expansion method, the heating module ensures the cooling effect in a quickly and easily regulatable manner. The valve nozzle mounted in the cooling chamber (heating module) is opened by activation via a pressure element, and spraying may be performed from the gas canister directly onto a thermal cap mounted on the grip head. The cold generated in this way is further transmitted to the bone implant, thus cooling the bone implant to the desired minus temperature. By use of a sensor, the temperature at the grip head may be read at any time, and thus, may be accordingly cooled by pressing on the activating head. The resulting residues are then discharged through a line.

[0051] In summary, the advantageous arrangement of the heating module also ensures that the generation of cold always takes place within the cooling module, i.e., a closed circuit. Thus, it is not possible for the cooling substances, which often are very toxic, to contact the body, and accordingly, the application of cold as a principal element of the invention is also possible at any time in any desired quantity. Importantly, this process thus makes a nonharmful procedure possible for the patient and the attending medical practitioner.

[0052] Empirical tests have shown that the transfer of cold from the heating module to the grip head and into the bone implant is surprisingly rapid, and may be carried out within a period of time (approximately 2-3 minutes) that is reasonable for patients as well as surgeons. Likewise, computer animation of cooling a bone implant at the head shows that the cold may be transmitted surprisingly quickly through the cross section of the implant head, surrounded by a body temperature of approximately 36° C., to the implant tip. The calculated time is less than one minute.

[0053] According to the invention, the grip head may be designed in a technically advantageous manner in such a way that even influences from aging, an angled geometry, or other factors do not greatly increase the theoretically calculated time. Of course, the application of cold while manipulating the device is dependent on the shape, the length, etc. of the implant. However, since the effect of cold is important for detaching the implant surface from the bone tissue, a somewhat longer cooling time may be accepted.

[0054] By means of the further detachment process, the interfaces between the bone mass and the implant are gently detached from one another or further loosened by supplying precisely metered vibration energy which is adequately adapted to the particular bone implant. In this case, the proper holding of the implant head by the grip head transmits the necessary mechanical energy, preferably vibrations, for detaching the surfaces from one another. The design of the grip head which is adapted to the shape of the particular implant head allows use to be made of the vibration energy, transmitted through the implant head, as a second element for detaching or loosening the implant from the bone.

[0055] The vibration energy may be generated directly next to the handle, from which it is guided to the grip head. However, externally generated vibration energy may also be guided through the grip head. The vibration energy may be generated, for example, using an electric motor (6000-40,000 rpm) having an eccentric. It is thus possible to generate vibrations in the handle in the frequency range of approximately 100-700 Hertz, and to transmit same to the grip head tool. Higher-frequency vibrations may be generated if needed, for example using piezo crystal vibration elements. The frequency range in this case preferably varies from 5 kHz to 30 kHz.

[0056] The energy generated by vibrations is converted into heat in the tissue. Possible heat generation with adverse effects on healing is prevented by prior and/or simultaneous cooling using the device according to the invention.

[0057] Within the scope of the invention, the type of vibrations which are best suited for detaching the surfaces may be set by selecting a suitable output, and thus, a certain power of the drive motor and a design and weight of the eccentric. The vibrations independently propagate through a punctiform bridge from the holder of the grip head into the bone implant. Thus, even relatively long bone implants, up to the tip, are brought into this state so that detaching is effective, as well as the cold energy in its transmission in exchange with the holder of the implant which surrounds the body. Thus, for very long implants, when a certain length of the bone implant is exceeded it is possible that the cooling which consistently occurs by dissipating the corresponding heat energy from above, over the implant head, may no longer be able to propagate. At the location in question, the energy dissipation resulting from cooling and the new supply from the surrounding body tissue maintain a balance. In particular in this case as well, the vibration for reliable detachment is also very important beneath this location. The vibrations may be introduced into the bone implant using a piezo vibration unit, for example.

[0058] The combined effects of cooling and vibration, which mutually reinforce one another in the detachment effect, are thus used at the same time within the scope of the invention. Thus, the necessary prerequisites are provided in order to subsequently employ the torque effect using the device. In this regard, it is advantageous when an angle between the rotational axis and an axis perpendicular to the longitudinal axis of the handle is less than 30°, preferably less than 15°. As the result of a twist-resistant mounting between the grip head and the bone implant, and with the aid of the long lever formed by the handle, the bone implant is removed from its mounting in the counter-screwing direction. Due to the preceding cold and vibration effect, the surfaces have been detached from one another beforehand so that the bone implant may be easily unscrewed without damaging the bone tissue.

[0059] After the cooling process has been completed, the vibration energy is generated next to the handle, and subsequently a torque is provided via the grip head for unscrewing the implant or for final detachment of the connection between the implant and the bone. The previous detachment or weakening of the connection between the bone and the implant surface by cooling and subsequent vibration allows an implant or a bone screw to be unscrewed with as little damage as possible to the surrounding bone, and with a lower required torque. A rotation carried out using the handle, preferably in the direction of rotation opposite the screwing direction, and also by fine back-and-forth tilting and rocking, allows an extremely gentle explantation of the bone implant.

[0060] The anti-twist resistance between the grip head and the implant may be increased, for example, by axially drilling through the outer shell of the grip head and the implant head and establishing a positive-fit connection with a pin inserted into this borehole for additional (frictionally engaged) clamping. The explantation of ceramic implants is thus easily performed using the device according to the invention.

[0061] The explantation may thus be carried out in a gentle manner using cold, vibration, and torque without the entire bone mass, which has grown between thread pitches and at the lateral layer surface of the implant, also being pulled out.

[0062] As mentioned at the outset, the novel device is usable for the explantation of most bone implants which in principle have a similar design. It is crucial not only that use is made of an individual detachment procedure, but also that the device according to the invention can enable the desired detachment of the implant surface and the bone tissue from one another by using various procedures. The bone implant is gripped and enclosed by a grip head. This docking of the grip head ensures the loss-free transmission of the cold and mechanical energy to the bone implant. For the explantation of dental implants, the grip head is designed in such a way that the procedure is also possible between two neighboring teeth. The device is usable for both the upper and the lower jaw, and only requires the appropriate manipulation.

[0063] As a supporting unit, the handle is advantageously dimensioned to be sufficiently large and designed in such a way that the vibration and cold energy may be generated directly in the handle, and in particular the cooling module and the vibration module are at least partially accommodated in the handle. The handle design may also allow externally

generated thermal energy and also the vibrations to be led through the handle and to the grip head. The handle is angled with respect to the grip head, and in its final function, i.e., after the cooling phase and after the vibration phase, the surgeon may rotate and tilt the handle about the longitudinal axis of the grip head.

[0064] The device according to the invention combines the effects of cold, vibrations, and a powerful final unscrewing torque via the handle. The device preferably includes another, fourth element, namely, hammer blows, which may be generated by a hammering module. For this purpose, an eccentric is preferably mounted on a motor axle for the basic vibration, together with a gearwheel which drives a pulse wheel. By means of a pin secured to this wheel, a striking hammer is tensioned which, after the circular travel of the pin, causes the hammer to strike the support housing. This hammer may be used for the fine hammering resonance of the device in the unscrewing direction, and with a second hammer may also be used in the screwing direction.

[0065] In one preferred embodiment, the hammering module has a movable hammer head and a stationary anvil, the hammer head periodically striking the anvil, and a striking direction of the hammer head preferably being essentially perpendicular to the rotational axis which is defined by the seat.

[0066] This fourth detachment dimension may be intentionally initiated after carrying out the chilling phase (cold and vibrations), and also has an additional positive effect as detachment energy. This hammering vibration has a quite unexpected additional detachment effect, since the lever arm is relatively long in relation to the longitudinal axis of the bone implant, and due to the high impact frequency, this “small” rotation effect using the large handle prepares the connecting surfaces extremely effectively for the final application of the detachment force of the handle, in both the unscrewing and screwing directions. The hammering direction, the same as for the mechanical device for unscrewing, initially acts in the direction opposite the screwing direction of the bone implant. The impact force may be influenced and regulated via the mass of the striking pin. However, the same unit may also be easily mounted on the opposite side, but in the other direction of the bone implant, with the same effect. The frequency of the electromagnetic striking units may in each case be set at the handle.

[0067] As a fifth dimension, a forceps handle as a clamping handle may also be mounted on the main handle. By squeezing the forceps handle and the main handle together, in each case two clamping jaws are activated which act on the bone implant radially inwardly with respect to the longitudinal axis. As a secondary clamping force, the corresponding clamping force may overlap a basic clamping force which is generated by other means. The clamping jaws are advantageously designed in such a way that they firmly grip the dental implant on both sides, but are narrow enough that teeth to the left and right allow sufficient space for this clamping jaw variant. The effect of the vibration as well as the cold and the large unscrewing torque, and the impact vibration in the unscrewing direction are also transmitted via these clamping jaws, and optionally an adapter, to the implant. The device according to the invention may thus be used in many ways, and the main action elements or dimensions of cold, vibration, primary torque, impact vibrations, and the holding by two clamping jaws ensure the versatility of the device.

[0068] Further advantageous embodiments and feature combinations of the invention result from the following detailed description and the entirety of the patent claims.

[0069] In the future, gentle and simple explantation must be established as a general quality standard for the placement of implants. As a rule, it must therefore be possible for the explantations to be carried out in a predictable, gentle, and risk-free manner, so that a new implantation is easily possible in the shortest possible time. Successful innovations often make it difficult to simultaneously address the successful management of the already expected statistical failure for the implantation. Optimal methods and devices will be of paramount importance in the future, not only for the use of implants and an improved connection with the bone tissue, but also for simple, gentle removal. Based on standards, when using any new implant the surgeon must have the expertise, training, and the appropriate devices for proper explantation.

BRIEF DESCRIPTION OF THE DRAWINGS

[0070] The drawings used to explain the exemplary embodiment show the following:

[0071] FIG. **1** shows a schematic overview illustration of one exemplary embodiment of the device according to the invention, in an isometric view;

[0072] FIG. **2** shows a schematic illustration of the exemplary embodiment according to FIG. **1**, in a side view along plane A-A indicated in FIG. **3**;

[0073] FIG. **3** shows a schematic illustration of the exemplary embodiment according to FIG. **1**, in a top view in partial cross section;

[0074] FIG. **4** shows a schematic overall side view of the exemplary embodiment;

[0075] FIG. **5** shows section B-B from FIG. **4**;

[0076] FIG. **6** shows section C-C from FIG. **4**;

[0077] FIGS. **7***a-c* show schematic partial views of a grip head for the exemplary embodiment; and

[0078] FIGS. **8***a-c* show schematic partial views of a further grip head for the exemplary embodiment.

[0079] Identical parts are basically provided with the same reference numerals in the figures.

APPROACHES FOR CARRYING OUT THE INVENTION

[0080] FIG. **1** shows the schematic overview illustration of one exemplary embodiment of the device according to the invention, in an isometric view. The device is composed essentially of the following functional units: grip head **6**, heating or cooling module **7**, vibration module **8**, hammering module **9**, handle **10**, and a secondary clamping system composed of a clamping lever **11** and clamping jaws **12**, **13** situated on both sides.

[0081] The grip head **6** is designed as a clamping part, and may be docked in an analogously designed seat (interface) on the base device (detailed description in conjunction with FIGS. **2** and **3**). The implant screw head (or a correspondingly designed intermediate adapter) may be inserted into the cylindrical hole **14** and initially lightly clamped by the clamping screw **15**. A basic clamping force is thus provided by the clamping screw **15**.

[0082] The heating or cooling module **7** may be activated for cooling, or alternatively, for heating (a detailed description is provided in conjunction with FIGS. **2** and **3**).

[0083] After an appropriate period of exposure from the cooling/(heating) phase, the vibration generated by a vibration generator may be switched on by an on/off switch **16** which is preferably integrated into the handle, the plane of action of the vibration being situated perpendicular to the implant screw axis. The desired vibration frequency may preferably be adapted using an integrated controller, which may be adjusted via a selector switch **17**.

[0084] Overlapped with or staggered in time with respect to same, an on/off switch **18** which is preferably likewise integrated into the handle may be used to switch on the hammering which is generated by an appropriately designed drive, the plane of action of the hammering being situated perpendicular to the implant screw axis, and the hammer blows which act at a radial distance as a peripheral force cause rhythmical torque pulses about the implant screw axis. The desired hammering frequency may preferably be adapted using an integrated controller, which may be adjusted via a selector switch **19**.

[0085] After the cooling/(heating), vibration, and hammering phases (or optionally also overlapped with respect to time), the handle **10** may be used to exert, over the entire device, a manually generated unscrewing torque about the longitudinal axis of the implant screw which is clamped in the grip head **6**; in addition, manually generated rocking motions in various directions may be simultaneously or sequentially exerted on the implant. For these manually generated force action phases it is advantageous to increase the primary clamping effect generated on the grip head **6** by a clamping screw **15** in order to allow large transmittable friction and holding forces via an additional secondary clamping. For this purpose, a manually activated clamping system is preferably combined with the handle in an ergonomically meaningful configuration. A clamping lever **11**, which is manually activated by a finger grip and which is supported so as to be pivotable about axle pins **20** which are arranged symmetrically on both sides, presses with the pressure fingers **21**, which are likewise arranged symmetrically on both sides, on the contact surfaces **22** of the upper and lower clamping jaw **12** (left) and clamping jaw **13** (right) which are pivotably supported by axle pins **23**. Due to their pivotability, the two clamping jaws transmit this introduced force, and with their pressure noses **24** symmetrically press on the longitudinally slitted grip head **6** on both sides, thus generating the additional (secondary) clamping effect on the implant head seated in the receiving hole **14**. By means of the elements arranged in the illustrated configuration, the leverage of the clamping lever **11** and the pressure fingers **21** allows a large transmission of the force which is manually introduced at the lever **11**. As a result of this secondary clamping system. it is possible for the clamping forces to be finely metered by the touch of the surgeon during the manual force action phases (unscrewing torque and rocking motions).

[0086] FIG. **2** shows a schematic illustration of the exemplary embodiment according to FIG. **1** in a side view, as a sectional illustration A-A from FIG. **3**.

[0087] An implant screw **1** (having a cylindrical head **25** as an exemplary embodiment) is screwed into the bone tissue **4**. Via the cylindrical receiving hole **14**, the grip head **6** is mounted on the cylindrical implant head **25** and firmly clamped via the clamping screw **15** (primary clamping).

[0088] In the front part, the explantation device according to the invention contains the heating or cooling module **7**, which in the illustrated exemplary embodiment is composed of a metallic holding sleeve **26** which has the highest possible thermal conductivity and has a central receiving hole **27** situated in the longitudinal axis; the grip head **6** is docked in the holding sleeve by pushing the elongated shank **6***a* against the explantation device. The holding sleeve **26** is embedded, preferably cast, in the surrounding housing casing **28**, which is made of heat-insulating material, preferably thermoplastic. In the illustrated exemplary embodiment, a blind hole **29** is provided obliquely from above at the rear end of the holding sleeve **26**, and continues obliquely to the rear in the same axis, aligned in the housing casing **28**. The housing part which extends obliquely rearwardly/upwardly forms a connector **30**. In the illustrated exemplary embodiment, at the rear end of the connector **30** a connecting flange **31** having holding tabs **32** is designed in such a way that a cover plate **33**, which is provided with retaining cams **34** as a counterpart to the holding tabs **32**, may thus be easily detachably connected with a ¼ revolution according to the known bayonet lock principle.

[0089] When the cover **33** is removed, a dry ice body **36** (compressed CO_2 snow) preformed to fit may be pushed into the receiving hole, which at the rear end is preferably provided with a funnel-shaped expansion **35**, and after the cover **33** is mounted, with the aid of a compression spring **38** supported in the rear of the cover base the dry ice body is pushed forward by an interior piston plunger **37** into the blind hole, and at this location delivers cold to the holding sleeve **26** by direct contact, and due to the good thermal conductivity properties the holding sleeve conveys the cold to the interior shank **6***a* of the grip head **6**, resulting in a cold flow **39** toward the front to the clamped implant head **25**, by means of which the metallic implant screwed into the bone tissue may be cooled. The dry ice body which is inserted for each application may be specifically dimensioned for any explantation procedure, so that it is able to deliver the necessary cooling capacity for the particular implant to be explanted. Since during the delivery of cold, the state of aggregation of the dry ice body changes directly into gaseous CO_2 (sublimation), appropriately dimensioned gas outlet openings **40** are provided at the housing casing.

[0090] Instead of the cooling with dry ice bodies, the cooling module **7** having a connector may be constructed in such a way that the connecting flange **31** is designed as a connecting point for a pressurized gas vial which may be coupled at the location of the cover **33**. This pressurized gas container may be filled with gas, for example CO_2 gas or other suitable gases frequently used for cooling purposes. This gas passes through a valve which is additionally integrated into the connector **30** and which is preferably activated via a pushbutton, and passes through an appropriately dimensioned hole to the holding sleeve **26** to be cooled. The cooling effect is caused by expansion of the gas. After the cooling energy is delivered, the gas may exit from the housing via appropriately designed exhaust openings **40**.

[0091] The part of the elongated grip head shank **6** protruding forwardly from the front section of the housing may be enclosed by an insulating sleeve **41**, made of thermally insulating material, which reduces the undesired discharge of cold to the surroundings. The insulating sleeve **41** has recesses on both sides for the pressure noses **24** described for FIG. **1**, the pressure noses pressing on the lateral pressure surfaces **42** mounted on the grip head shank **6***a* when the secondary clamping system is activated.

[0092] In the unactivated position, the clamping lever **11** is pushed back into the illustrated base position by a restoring spring **43**. Various electrical components, roughly schematically illustrated, which are necessary for the functioning of the device according to the invention are installed in the inner cavity of the handle **10**: the switching contacts **16***a*, **18***a* for the corresponding on/off switches **16**, **18**, an electronic printed circuit board **44** for all required switching and control functions, optionally a chargeable battery **45** for cordless operation of the device, and optionally as an alternative or in addition to the unbalanced oscillating drive described for FIGS. **1**, **3**, **6**, an installed piezo oscillating drive **46** which preferably generates vibration pulses **47** directed within the longitudinal axis, i.e., transversely with respect to the implant axis. The hollow handle **10** is provided with a housing cover **48**, which by means of a seal **49** seals off the interior from entry of cleaning fluid and steam during sterilization of the device. A connecting cable **50** for supplying electrical power is preferably inserted through this cover. In the case of a design having a battery, the required charging cable is preferably connected by a plug-in connection, which is mounted in the rear of the cover, for charging the device.

[0093] FIG. **3** shows a schematic illustration of the exemplary embodiment according to FIG. **1**, in a top view in partial cross section. The grip head **6** with its elongated shank **6***a* in the holding sleeve **26** is docked on the device. The grip head shank **6***a* has a slit **51**, extended toward the rear, which allows elastic bending of the two shank halves, so that when the pressure noses **24** on both sides are symmetrically pressed onto the pressure surfaces **44** on both sides, the implant head seated in the receiving hole **14** (or corresponding intermediate adapter) is additionally clamped (secondary clamping system). For dental applications, the grip head **6** may have a narrow design so that enough space remains between two neighboring teeth **5** to perform a small unscrewing motion (pivot angle) for loosening the implant screw.

[0094] A known principle of the vibration generation, which preferably occurs due to an unbalanced body **53** (eccentric) which is secured to a rotating axle **52** in a rotationally fixed manner, is illustrated in a partial cross section of the vibration module **8**. A vibration plane situated perpendicular to the implant screw axis results from the rotating axle **52** lying parallel to the implant screw axis. If desired, the device may be designed with a vibration plane which is not perpendicular to the implant screw axis; the vibration module **8** may also be optionally brought into various angular positions by an adjustable attachment to the device.

[0095] Instead of the rotating imbalance, other known principles for generating vibration are conceivable, such as a piezo crystal element. The electromagnetic oscillation of a vibrating body due to a controlled electrical alternating frequency on a magnetic coil likewise results in directed vibration pulses, similarly as for the piezo principle.

[0096] A mechanical principle of the generation of hammer blows is illustrated as an exemplary embodiment in a partial cross section of the hammering module **9**. As the result of a control cam **54** which is secured in a rotationally fixed manner to an axle **55** which rotates in the arrow direction **55***a*, a hammer head **56** which is preferably made of a heavy metal and which is secured to a leaf spring **57** that is pretensioned toward the center is periodically pushed outwardly, and after the highest cam position is overrun, due to the spring tension force is thrown inwardly onto the impact surface of the anvil **58** which is fixedly connected to the housing; the leaf spring end **60** situated opposite from the hammer head is fixedly anchored to the housing. Directed hammer blows **59** are thus generated which act at a radial distance as peripheral force pulses about the implant screw axis, thus generating torque pulses on the implant screw to be loosened, the frequency of which is proportional to the rotational drive speed of the rotating axle **55**.

[0097] Instead of the mechanical generation, other known principles of hammer blows **59** are conceivable, for example pulse generation by a piezo crystal element. The electromagnetic oscillation of a vibrating body due to alternating frequency on a magnetic coil likewise results in directed force pulses.

[0098] FIG. **4** shows a schematic overall side view of the exemplary embodiment. Apparent here are the rotational axes **63**, **64**, extending parallel to the implant screw axis **61**, of the vibration generation systems of the vibration module **8** and of the hammering module **9** described in FIG. **3**, as well as the cooling/heating module **7** together with its connector **30**.

[0099] As an embodiment variant of the cooling system shown in FIG. **2**, an exemplary embodiment having a cooling or heating circuit with a suitable liquid transfer medium (CFC-free refrigerant, for example) is illustrated here. A so-called twin hose **65** having two integrated tube channels is connected at an appropriately designed connecting flange **31** via a hose connection coupling **64**, by means of which a circuit having a forward flow line **66** and a reverse flow line **67** of the transfer medium is formed, the transfer medium being conducted inside the housing to the holding sleeve **26** and flowing around same via a suitably shaped, for example spiral-shaped, flow channel, resulting in cold or heat exchange toward the front with respect to the grip head. Outside the device, this circuit may be conducted via a suitable cooling or heating unit, inside of which the liquid transfer medium is supplied with the appropriate cold or heat energy.

[0100] By means of suitable components connected in-between in the circuit, such as an electric solenoid valve, pump (hose pump or diaphragm pump, for example), the heating module may be switched on/off as desired, and optional cooling or heating may be alternatively used.

[0101] Instead of a cooling/heating circuit having an external connection, the cooling or heating may be carried out using electrical power which is supplied via the connecting cable **50**. For heating, known principles, for example, are also conceivable which may be integrated into the device in direct contact with the outer shell of the holding sleeve **26**, for example the use of an electric heating cartridge, an electric heating coil, or a Peltier element (suitable for cooling or also possibly for heating).

[0102] FIG. **5** shows section B-B from FIG. **4**. Apparent here are the hand **75** of the surgeon, which encloses the handle **10** with the thumb **76** and fingers **77**, which optionally by gripping with individual fingers surround the clamping lever **11** and press upwardly against the handle (position **11***a*), by means of which the secondary clamping system may be activated. This manipulation analogously corresponds to the known activation process of a bicycle brake lever.

[0103] FIG. **6** shows section C-C from FIG. **4**. This figure illustrates the modules **8** (vibration), **9** (hammering), and parts of the heating module **7** (cooling/heating) in cross section. The holding sleeve **26** embedded in the surrounding thermally insulating housing **28** together with the grip head shank **6***a* inserted therein are visible in the area of the heating module **7**. A pin **78** which protrudes radially on one side is

preferably mounted in the grip head shank **6***a*, the pin being pushed through a longitudinal groove **79** provided in the longitudinal direction in the hole in the holding sleeve **26**, when the grip head shank is pushed in, and which in the rear impact position is pushed through a transverse groove **80**, provided at an appropriate depth in the sleeve hole and extending radially by an angle of 90° to the right, by a corresponding 90° rotation of the pushed-in grip head **6** positions same in the correct angular position according to the known bayonet lock principle and at the same time secures it from being pulled out. A groove **81** extending radially to the left is also provided in the holding sleeve hole, preferably in a mirror-image configuration with respect to the groove **80** extending radially 90° to the right, by means of which the grip head may optionally also be positioned and locked by a 90° rotation to the left. This allows the grip head to optionally also be used so that it is directed upwardly, rotated by 180° (use in the lower/upper jaw).

[0104] The vibration module **8** illustrated here in cross section shows the drive motor **68** installed with a vertical axis **63** and having a downwardly projecting rotating axle **52** on which the unbalanced body **53** is mounted in a rotationally fixed manner. After the drive is installed, the motor compartment, which is open at the top in the illustrated exemplary embodiment, may be hermetically sealed with a cover **69** and a seal **70**, and is thus suited for wet cleaning and sterilization of the overall device. Electrical connecting cables to the motor are preferably guided inside the housing to the switching/control electronics system installed in the handle.

[0105] The hammering module **9** illustrated here in cross section shows the drive **71** installed with a vertical axis **64** and having a downwardly projecting rotational axle **55** on which the control cam **54** is mounted in a rotationally fixed manner, as well as a cross section of the leaf spring **57**, at whose end the hammer head **56** illustrated in FIG. **3** is attached. After the drive is installed, the motor compartment, which is open at the top in the illustrated exemplary embodiment, may be hermetically sealed with a cover **72** and a seal **73**, and is thus suited for wet cleaning and sterilization of the overall device. Electrical connecting cables to the motor are preferably guided inside the housing to the switching/control electronics system installed in the handle.

[0106] FIG. **7** shows schematic partial views of the grip head **6** in a side view in partial cross section (FIG. **7***a*) and in a view from below (FIG. **7***b*), and section D-D (FIG. **7***c*) shows an embodiment variant of the grip head for use on implant screws having a polygonal socket head. The implant head is not directly clamped in the grip head **6**; instead, an additional polygonal intermediate adapter **82** is clamped. The intermediate adapter **82** is inserted with its polygonal head **84**, which is specifically designed to congruently fit the particular implant screw, into the implant screw **2** having a polygonal socket head. Such implant screws are typically provided with a deeper-seated internally threaded blind hole **87**. The adapter piece may be fixedly secured to the implant screw using a screw **85** which is inserted through the central through hole **86** in the adapter **82**. For a secure seat of the adapter in the implant screw, the polygonal head **84** may preferably be designed having conicity **88**, which in the topmost part is slightly overdimensioned with respect to the implant polygonal socket, and which results in a secure, play-free seat of the adapter when the center tensioning screw **85** is tightened.

[0107] The cylindrical adapter head **83** is then inserted into the receiving hole **14** in the grip head **6**, and by means of the clamping screw **15** is initially lightly clamped, and subsequently, during the force introduction phases, is additionally clamped by the above-described secondary clamping system as a result of the pressure noses **24** acting symmetrically on the lateral pressure surfaces **42** with the aid of the elasticity of the grip head shank **6***a* made possible by the slit **51**.

[0108] FIG. **8** shows schematic partial views of a further grip head **6** in a side view in partial cross section (FIG. **8***a*), in a view from below (FIG. **8***b*), and in cross section E-E (FIG. **8***c*). FIG. **8** shows an embodiment variant of the grip head for use on implant screws having a mushroom head-shaped head. The implant head is not directly clamped in the grip head **6**; instead, an additional mushroom head intermediate adapter **89** is clamped.

[0109] The intermediate adapter **89**, which is specifically designed to fit the particular implant screw, with its congruent prismatic inner groove **94** corresponding to the mushroom head geometry, is mounted on the implant screw **3** having a mushroom head **90**. The adapter is preferably divided in two over its entire length by an oblong slit **91**, and at the upper end preferably has support ribs **92** on both sides which support one another in the middle. These support ribs are preferably provided with an inwardly directed convex curvature so that they may gently roll over one another.

[0110] Instead of a two-part adapter, a one-piece part may optionally [be used], which with one central or multiple radially star-shaped oblong slit(s) is/are connected in the topmost part of the cylindrical shank **95** by a remaining material web. This results in a part provided with elastically resilient segments, analogous to the known collet chuck principle, which due to its elasticity may be snapped onto the mushroom head of the implant screw.

[0111] The adapter piece **89** preferably has a central through hole **86** which at the bottom has a larger opening **93**, which allows better machining of the prismatic inner groove **94** which is to be precisely adapted to the mushroom head geometry.

[0112] The cylindrical shank **95** of the mushroom head adapter is then inserted into the receiving hole **14** in the grip head **6**, and is initially lightly clamped by the clamping screw **15**. If the implant screw has an internally threaded blind hole, optionally as shown in FIG. **7**, the adapter piece may be additionally secured to the implant screw by inserting a screw **85** through the through hole **86**. In the subsequent force introduction phases, the adapter may be additionally clamped by the above-described secondary clamping system.

1. Device for detachment and explantation of bone implants, in particular tooth implants, from their connection to bone tissue, comprising:

a unit having

a) an elongated handle;

b) a cooling module;

c) a vibration module for generating mechanical vibrations;

d) a grip head which is secured to the handle and which has a seat for the a bone implant to be explanted, an adapter piece for gripping the bone implant;

wherein

e) the grip head is coupled to the cooling module and to the vibration module in such a way that it is coolable to a desired temperature of via the cooling module, and the vibrations of the vibration module are transferable into the grip head and further to the bone implant or the adapter piece;

f) the seat in the grip head has at least one clamping element which is able to exert radial clamping forces, with respect to a rotational axis, on the bone implant or adapter piece held in the seat;

g) the rotational axis is situated at an angle, with respect to a longitudinal axis of the handle; and wherein

h) the grip head is coupled to the handle in such a way that a manually applied rotational motion of the handle about the rotational axis is transferable to the bone implant or the adapter piece.

2. Device according to claim **1**, wherein an angle between the rotational axis and an axis perpendicular to the longitudinal axis of the handle is less than 30°, preferably less than 15°.

3. Device according to claim **1**, wherein the seat is essentially cylindrical, the rotational axis corresponding to the cylindrical axis.

4. Device according to claim **1**, wherein the grip head is directly secured to a distal end of the handle.

5. Device according to claim **1**, wherein the seat of the grip head includes multiple clamping jaws, a basic clamping force being capable of being provided by first means.

6. Device according to claim **5**, wherein a clamping handle is situated on the handle, of via which a manually generated additional clamping force is transferable to the clamping jaws.

7. Device according to claim **1**, wherein the handle is designed as a supporting element, and the cooling module and the vibration module are at least partially accommodated in the handle.

8. Device according to claim **1**, wherein the cooling module includes a receptacle for a cooling body.

9. Device according to claim **1**, wherein the vibration module may generate vibrations having a vibration plane which is perpendicular to the rotational axis.

10. Device according to claim **1**, wherein a hammering module via which hammer blows are capable of being generated, the grip head being coupled to the hammering module in such a way that the hammer blows are transferable to the grip head and further to the bone implant or the adapter piece.

11. Device according to claim **10**, wherein the hammering module has a movable hammer head and a stationary anvil, the hammer head periodically striking the anvil, and a striking direction of the hammer head preferably being essentially perpendicular to the rotational axis which is defined by the seat.

12. The device according to claim **1**, wherein the bone implant to be explanted is an implant head of the bone implant.

13. The device according to claim **1**, wherein the rotational axis is situated at an angle at essentially a right angle.

14. The device according to claim **8**, wherein the cooling body is a dry ice body.

15. A method for loosening/detaching and explanting bone implants, in particular tooth implants, from their connection to bone tissue, comprising:

griping the bone implant with the grip head of the device of claim **1**, wherein

(a) the grip head is cooled to a desired temperature via the cooling module, and the vibrations of the vibration module are transferred into the grip head and further to the bone implant or the adapter piece;

(b) the at least one clamping element of the seat in the grip head exerts radial clamping forces, with respect to a rotational axis, on the bone implant or adapter piece held in the seat; and/or

(c) a rotational motion about the rotational axis applied manually via the handle is transferred to the bone implant or the adapter piece.

16. The method according to claim **15**, wherein the seat of the grip head of the device includes multiple clamping jaws and a basic clamping force is provided by first means.

17. The method according to claim **16**, wherein a clamping handle is situated on the handle, and a manually generated additional clamping force is transferred to the clamping jaws.

18. The method according to claim **15**, wherein the vibration module generates vibrations having a vibration plane which is perpendicular to the rotational axis.

19. The method according to claim **15**, wherein the device comprises a hammering module via which hammer blows are generated, and the grip head is coupled to the hammering module, wherein the hammer blows transferred to the grip head and further to the bone implant or the adapter piece.

20. The method according to claim **19**, wherein the hammering module has a movable hammer head and a stationary anvil and the hammer head periodically strikes the anvil.

* * * * *
