# Supplementary material for: Reversal of Osseointegration as a Novel Perspective for the Removal of Failed Dental Implants: A Review of Five Patented Methods
Source: Materials (Basel). 2021 Dec 17;14(24):7829. doi: 10.3390/ma14247829 (PMC8707383; doi:10.3390/ma14247829)
Supplement: Supplementary file 1 [file materials-14-07829-s001.zip › P3 (KR) Yonsei University, Korea, Lee Korean Original.pdf]

(19) 대한민국특허청(KR)
(12) 등록특허공보(B1)

(45) 공고일자 2011년02월24일
(11) 등록번호 10-1016177
(24) 등록일자 2011년02월14일

(51) Int. Cl.
A61C 8/00 (2006.01) *A61C 3/00* (2006.01)
(21) 출원번호 10-2009-0001556
(22) 출원일자 2009년01월08일
심사청구일자 2009년01월08일
(65) 공개번호 10-2010-0082188
(43) 공개일자 2010년07월16일
(56) 선행기술조사문헌
JP04133308 B2
KR200425722 Y1
KR100721603 B1
KR200414573 Y1

(73) 특허권자
연세대학교 산학협력단
서울 서대문구 신촌동 134 연세대학교
(72) 발명자
이정섭
강원도 원주시 일산동 162번지 연세대학교 원주기독병원치과교정과
(74) 대리인
양부현, 김승진

전체 청구항 수 : 총 8 항 심사관 : 조흥규

(54) 치과 임플란트 픽스츄어 제거용 디바이스

(57) 요 약

본 발명은 임플란트 픽스츄어를 가열하는 히터부, 임플란트 픽스츄어의 온도를 감지하는 온도센서부 및 온도센서부에 의해 감지된 온도에 의하여 히터부의 온도를 제어하는 제어부를 포함하는 치과 임플란트 픽스츄어 제거용 디바이스에 관한 것이다.

본 발명은 임플란트 시술 후 보철물 수복에 부적절하거나 치료 계획 변경등으로 인하여 식립된 임플란트 픽스츄어 제거시 골결손부를 최소화하고 제거 시술을 간단하게 할 수 있는 장점이 있다. 본 발명은 다양한 형태의 임플란트 픽스츄어에 적용할 수 있도록 분리 가능한 히터부를 갖추고 있으며, 임프란트 픽스츄어에 맞는 히터부를 사용함으로써 뛰어난 열전도율 및 에너지 효율을 나타낸다. 본 발명은 온도와 시간을 알리는 알림부가 있어서 식립된 임플란트 픽스츄어를 안전하고 빠르게 제거할 수 있다.

대 표 도 - 도1

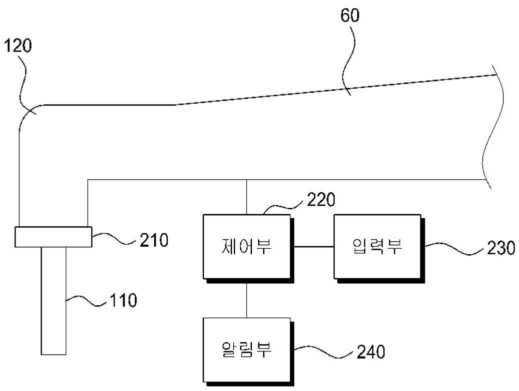

특허청구의 범위

청구항 1

치과 임플란트 픽스츄어(Implant Fixture) 제거용 디바이스에 있어서,

(a) 임플란트 픽스츄어를 가열하는 히터부;

(b) 상기 임플란트 픽스츄어의 온도를 감지하는 온도센서부; 및

(c) 상기 온도센서부에 의해 감지된 온도에 의하여 상기 히터부의 온도를 제어하는 제어부

를 포함하는 치과 임플란트 픽스츄어 제거용 디바이스.

청구항 2

제 1 항에 있어서,

상기 히터부는 상기 임플란트 픽스츄어의 내부와 면접촉이 가능한 형태를 가지는 것을 특징으로 하는 치과 임플란트 픽스츄어 제거용 디바이스.

청구항 3

제 1 항에 있어서,

상기 온도센서부는 외부로부터 열이 전달되는 것을 차단할 수 있는 단열부재를 포함하는 것을 특징으로 하는 치과 임플란트 픽스츄어 제거용 디바이스.

청구항 4

제 1 항에 있어서,

상기 제어부는 임플란트 픽스츄어를 기설정 온도까지 가열시키고, 일정시간 이상이 되는 경우 상기 히터부를 오프(off)시키는 것을 특징으로 하는 치과 임플란트 픽스츄어 제거용 디바이스.

청구항 5

제 4 항에 있어서,

상기 기설정온도는 40-60℃인 것을 특징으로 하는 치과 임플란트 픽스츄어 제거용 디바이스.

청구항 6

제 4 항에 있어서,

상기 일정시간은 상기 기설정온도에 도달한 후 상기 임플란트 픽스츄어의 주변 치조골이 괴사(osteronecrosis) 되는 시점의 시간인 것을 특징으로 하는 치과 임플란트 픽스츄어 제거용 디바이스.

청구항 7

제 1 항에 있어서,

상기 디바이스는 경보신호를 알려주는 알람부를 더 포함하고, 상기 제어부는 히터부의 온도 또는 임플란트 픽스츄어의 온도가 기설정된 온도 값에 도달 또는 기설정된 시간 값에 도달되는 경우, 상기 알람부가 경보신호를 표

출하도록 제어하는 것을 특징으로 하는 치과 임플란트 픽스츄어 제거용 디바이스.

청구항 8

제 1 항에 있어서,

상기 디바이스는 사용자 입력부를 더 포함하고, 상기 제어부는 사용자 입력부를 통해 입력된 온도 또는 시간 값에 따라 상기 히터부를 제어하는 것을 특징으로 하는 치과 임플란트 픽스츄어 제거용 디바이스.

명 세 서

발명의 상세한 설명

기 술 분 야

[0001] 본 발명은 치과 임플란트 픽스츄어 제거용 디바이스에 관한 것이다.

배 경 기 술

[0002] 임플란트 시술은 인체의 조직이 상실되었을 때 이를 회복시켜 주는 대치물을 의미하지만 치과에서는 인공치아 이식을 말한다. 치과 임플란트 시술은 상실된 치아의 치근을 대신할 수 있도록, 인체에 거부반응이 없는 티타늄으로 만든 인공치근을 치아가 빠져나간 치조골에 심어서 유착시킨 뒤 인공치아를 고정시켜 치아의 원래 기능을 회복하도록 하는 첨단시술이다.

[0003] 치과 임플란트 시술을 하는 경우 한번에 완료하는 경우에는 큰 문제가 발생하지는 않지만, 임플란트 픽스츄어 식립 후 보철물 수복에 부적절 하거나 치료계획을 변경해야 하는 경우에는 기존에 식립되어 있는 치과용 임플란트 픽스츄어를 제거해야 하는 경우가 발생한다. 이런 경우에는 통상적으로 트리파인 버(Trephine bur), 라운드 버(Round bur) 또는 피슈어 버(Fissure bur)를 주로 사용하여 식립되어 있는 임플란트 픽스츄어를 제거한다. 그러나, 이런 장비를 사용하는 경우에는 주위 골과 함께 광범위한 수술이 필요하며 수술 후에는 큰 골 결손부가 생겨 새로운 임플란트의 식립은 물론 가철성 의치의 장착 또한 어렵게 하는 결과를 낳는다.

[0004] 현재 지렛대원리를 이용하여 식립된 임플란트 픽스츄어를 간편하게 제거할 수 있도록 한 임플란트(implant)용 분리장치(대한민국특허 제721603호)나 임플란트 픽스츄어 걸림부에 추출공구를 결합시켜 빼냄으로써 임플란트 픽스츄어를 치조골에서부터 분리하는 기술이 개발되었다(대한민국실용신안 제414573호).

[0005] 하지만 전술한 바와 같이 치과 임플란트 금속 매식체를 제거하는 경우에 매식체 주변의 큰 결손부로 인한 문제점은 해결하지 못하고 있으며, 이러한 문제점을 해결하기 위한 장비의 필요성이 대두 되고 있다.

[0006] 본 명세서 전체에 걸쳐 다수의 논문 및 특허문헌이 참조되고 그 인용이 표시되어 있다. 인용된 논문 및 특허문헌의 개시 내용은 그 전체로서 본 명세서에 참조로 삽입되어 본 발명이 속하는 기술 분야의 수준 및 본 발명의 내용이 보다 명확하게 설명된다.

발명의 내용

해결 하고자하는 과제

[0007] 본 발명은 임플란트의 시술 안정성을 보장하고 시술의 편의성을 향상시킬 수 있는 치과 임플란트 픽스츄어 제거용 디바이스를 제공한다.

[0008] 특히, 본 발명은 적절한 온도를 일정 시간 식립된 임플란트 픽스츄어에 가해 고온에 의한 부분적 골괴 반응을 이용하여 치과 임플란트 금속 매식체 제거시 골결손부를 최소화하는 치과 임플란트 픽스츄어 제거용 디바이스를 제공한다.

[0009] 또한, 본 발명은 식립된 임플란트 픽스츄어 제거 시술을 간단하고 안정성이 뛰어난 치과 임플란트 픽스츄어 제

거용 디바이스를 제공한다.

[0010] 본 발명의 다른 목적 및 이점은 하기의 발명의 상세한 설명, 청구범위 및 도면에 의해 보다 명확하게 된다.

과제 해결수단

[0011] 본 발명의 일 양태에 따르면, 본 발명은 (a) 임플란트 픽스츄어를 가열하는 히터부; (b) 상기 임플란트 픽스츄어의 온도를 감지하는 온도센서부; 및 (c) 상기 온도센서부에 의해 감지된 온도에 의하여 상기 히터부의 온도를 제어하는 제어부를 포함하는 치과 임플란트 픽스츄어 제거용 디바이스를 제공하는데 있다.

[0012] 본 명세서 용어 '임플란트 픽스츄어'는 치과 임플란트 시술시 사용되는 인공치근을 의미한다. 상기 임플란트 픽스츄어는 바람직하게는 금속 또는 비금속으로 제작 될 수 있으며, 보다 바람직하게는 금속으로 제작된다. 가장 바람직하게는 티타늄(Titanium)을 재료로 사용된다.

[0013] 본 발명의 구현예에 따르면, 상기 히터부는 상기 임플란트 픽스츄어의 내부와 면접촉이 가능한 형태를 가지는 것을 특징으로 한다.

[0014] 본 발명의 구현예에 따르면, 상기 온도센서부는 외부로부터 열이 전달되는 것을 차단할 수 있는 단열부재를 포함하는 것을 특징으로 한다.

[0015] 본 발명의 구현예에 따르면, 상기 제어부는 상기 임플란트 픽스츄어를 기설정온도까지 가열시키고, 일정시간 이상이 되는 경우 상기 히터부를 오프(off)시키는 것을 특징으로 한다.

[0016] 본 발명의 구현예에 따르면, 상기 기설정온도는 40-60℃인 것을 특징으로 한다.

[0017] 본 발명의 구현예에 따르면, 상기 일정시간은 상기 기설정온도에 도달한 후 상기 임플란트 픽스츄어의 주변 치조골이 괴사되는 시점의 시간인 것을 특징으로 한다.

[0018] 본 명세서 용어 '골 괴사(osteronecrosis)'는 외부 열에 의한 골세포의 세포조직이 붕괴되거나 기능이 정지되는 물리적 괴사를 의미한다.

[0019] 본 발명의 구현예에 따르면, 상기 디바이스는 경보신호를 알려주는 알람부를 더 포함하고, 상기 기설정된 온도 또는 시간 값에 도달하는 경우, 상기 알람부가 경보신호를 표출하도록 제어하는 것을 특징으로 한다.

[0020] 본 발명의 구현예에 따르면, 상기 디바이스는 사용자 입력부를 더 포함하고, 상기 제어부는 사용자 입력부를 통해 입력된 온도 또는 시간 값에 따라 상기 히터부를 제어하는 것을 특징으로 한다.

효 과

[0021] 본 발명의 특징 및 이점을 요약하면 다음과 같다:

[0022] (ⅰ) 본 발명은 치과 임플란트 픽스츄어 제거용 디바이스에 있어서, (a) 임플란트 픽스츄어를 가열하는 히터부; (b) 상기 임플란트 픽스츄어의 온도를 감지하는 온도센서부; 및 (c) 상기 온도센서부에 의해 감지된 온도에 의하여 상기 히터부의 온도를 제어하는 제어부를 포함하는 치과 임플란트 픽스츄어 제거용 디바이스를 제공한다.

[0023] (ⅱ) 본 발명은 임플란트 시술 후 보철물 수복에 부적절하거나 치료 계획 변경등으로 인하여 식립된 임플란트 픽스츄어 제거시 골결손부를 최소화하고 제거 시술을 간단하게 할 수 있는 장점이 있다.

[0024] (ⅲ) 본 발명은 다양한 형태의 임플란트 픽스츄어에 적용할 수 있도록 분리가능한 히터부를 갖추고 있으며, 임프란트 픽스츄어에 맞는 히터부를 사용함으로써 뛰어난 열전도율 및 에너지 효율을 나타낸다.

[0025] (ⅳ) 본 발명은 온도와 시간을 알리는 알림부가 있어서 식립된 임플란트 픽스츄어를 안전하고 빠르게 제거할 수 있다.

발명의 실시를 위한 구체적인 내용

[0026] 이하 첨부된 도면들을 참조하여 본 발명의 바람직한 실시예를 상세하게 설명하지만, 본 발명이 실시예에 의해 제한되거나 한정되는 것은 아니다. 본 발명을 설명함에 있어서, 공지된 기능 혹은 구성에 대해 구체적인 설명은 본 발명의 요지를 명료하게 하기 위하여 생략될 수 있다.

[0027] 도 1은 본 발명에 따른 치과 임플란트 픽스츄어 제거용 디바이스에 관한 구성을 나타내는 도면이다. 상기 디바이스는 픽스츄어를 가열하는 히터부, 픽스츄어의 온도를 감지할 수 있는 온도센서부 및 온도센서부에 의해 감지된 온도에 의하여 상기 히터부의 온도를 제어하는 제어부로 구성된다.

[0028] 도 2는 본 발명의 히터부 및 온도센서부가 치과 임플란트 픽스츄어와 결합한 상태에서의 단면도이다.

[0029] 도 3 내지 도 6은 본 발명의 히터부와 온도센서부가 작동하여 치과 임플란트 픽스츄어를 제거하는 과정을 순차적으로 보여주는 도면이다.

[0030] 이하에서는 치과 임플란트 픽스츄어 제거용 디바이스로서, 보철물 수복에 부적절하거나 치료계획 변경등으로 인하여 식립된 치과 임플란트 픽스츄어를 제거하는 과정을 통하여 설명하기로 한다.

[0031] 상기 히터부의 구조에 있어서, 외부 형태는 통상적으로 원기둥 형태를 가질 수 있으며, 임플란트 픽스츄어의 내부 구조와 상응하는 다양한 모양의 형태를 가질 수 있다. 상기 히터부는 임플란트 내부와 점, 선 또는 면 접촉을 할 수 있는 형태를 가지는 것을 특징으로 한다.

[0032] 상기 히터부 내부는 자체 발열물질로 구성될 수 있으며, 열전도율이 높은 발열체를 포함할 수 있고, 내부와 외부가 분리되어 열전도율이 높은 물질을 포함시킬 수 있다.

[0033] 상기 히터부의 온도는 최소 40-1000℃의 열을 발산할 수 있다. 상기 히터부가 외부로 열을 발산하는 방식은 직접 또는 간접으로 발산하는 것이 가능하다.

[0034] 상기 히터부와 임플란트 픽스츄어로 직접 접촉하여 열을 전달하는 방식과 매질을 통하여 열을 전달하는 방식을 가질 수 있다. 상기 매질은 액체, 기체 또는 고체를 포함한다. 상기 열을 전달하는 방식은 조건 및 설계 사양에 따라 다양하게 변경될 수 있다.

[0035] 상기 히터부는 디바이스 또는 온도센서부와 탈부착이 가능하다. 히터부와 디바이스 또는 히터부와 온도센서부와의 연결 부위는 조건 및 설계 사양에 따라 다양하게 변경될 수 있다.

[0036] 상기 치과 임플란트 픽스츄어를 제거하는 경우 먼저 히터부를 픽스츄어 내부에 삽입 한 후 히터부를 발열시킨다. 이때, 상기 히터부와 픽스츄어와의 열전도율 및 에너지 효율을 높이기 위하여 상기 픽스츄어 내부에 매질체를 첨가시킬 수 있다. 바람직하게는, 상기 매질체로 액체, 기체 또는 고체를 사용할 수 있으며, 보다 바람직하게는 액체 또는 기체를 사용할 수 있고, 가장 바람직하게는 액체를 사용할 수 있다. 상기 매질체로 사용되는 액체는 통상적으로 치과 시술에 사용하는 인체에 독성이 없고 무해한 액체를 의미한다.

[0037] 상기 온도센서부는 픽스츄어의 온도를 감지할 수 있는 온도센서를 포함하고, 외부 열과 온도센서와의 열을 차단할 수 있는 단열부재를 포함한다. 온도 센서부는 디바이스와 히터부 사이에 작동적으로 연결(operatively linked)하여 구성되는 내장형과 분리되어 작동할 수 있는 외장형으로 구성될 수 있다.

[0038] 상기 온도센서는 픽스츄어의 온도를 아날로그 또는 디지털 방식으로 감지가 가능하고, 피접촉물과 직접 센서를 접촉시켜 온도를 감지하는 방식과 피접촉물에서 방사되는 적외선을 감지하는 방식으로 구성될 수 있다. 예를 들면, 치과 임플란트 픽스츄어에 직접 센서를 접촉시켜 온도를 감지하여 제어부로 전기적 신호를 전달하거나 치과 임플란트 픽스츄어 또는 주변 잇몸에서 방사되는 적외선을 감지하여 제어부로 전기적 신호를 전달한다.

[0039] 상기 단열부재는 통상적으로 열을 차단할 수 있는 재료로 구성될 수 있으며, 임상적으로 안전하고 검증된 재료라면 어느 것이든 가능하다. 상기 단열부재는 온도센서를 외부로 부터 열을 차단하기 위하여 온도센서 주위를 둘러싸는 형태로 구성될 수 있다.

[0040] 상기 제어부는 핸드피스에 포함될 수 있으며, 분리되어 독립적으로 구성될 수 있다.

[0041] 상기 제어부는 임플란트 픽스츄어를 기설정온도까지 가열시키고, 일정시간 이상이 되는 경우 히터부를 오프(off)시켜 제어하는 것을 특징으로 한다. 상기 기설정온도는 40-60℃이며, 보다 바람직하게는 45-50℃이며, 가장 바람직하게는 46-47℃인 것을 의미한다. 상기 일정시간은 임플란트 픽스츄어를 기설정온도까지 가열한 후 픽스츄어의 주변 치조골이 괴사가 발생되는 시점인 것을 의미한다.

[0042] 상기 디바이스는 경보신호를 알려주는 알람부를 더 포함하고, 기설정된 온도 또는 시간 값에 도달하는 경우, 알

람부가 경보신호를 표출하도록 제어하는 것을 특징으로 한다. 경보신호는 소리, 빛 또는 진동으로 표출하고, 사용자는 이러한 경보 신호를 바탕으로 임플란트 디바이스의 사용을 판단 할 수 있다.

[0043] 상기 디바이스는 사용자 입력부를 더 포함하고, 제어부는 사용자 입력부를 통해 입력된 온도 또는 시간 값에 따라 상기 히터부를 제어하는 것을 특징으로 한다.

[0044] 이하에서는 본 발명에 따른 치과용 수술 장치의 작동 구조를 설명하기로 한다. 도 7은 본 발명에 따른 치과용 수술 장치의 작동 구조를 설명하기 위한 블록도이다. 아울러, 전술한 구성과 동일 및 동일 상당 부분에 대해서는 동일 또는 동일 상당한 참조 부호를 부여하고, 그에 대한 상세한 설명은 생략하기로 한다.

[0045] 도 7에 도시한 바와 같이, 시술자는 상기 입력부를 통해 온도와 시간을 설정하고, 상기 치과 임플란트 픽스츄어 제거용 디바이스의 히터부를 상기 임플란트 픽스츄어 내부에 삽입을 한 뒤 히터부를 발열시킨다. 이때, 상기 히터부를 삽입하기 전 픽스츄어 내부에 매질체를 첨가하여 열전도율과 열효율을 증대시킬 수 있다.

[0046] 상기 치과 임플란트 픽스츄어의 온도가 기설정된 온도 또는 시간에 도달하는 경우에는 상기 알림부로 전기적 신호를 보내어 최종적으로 시술자에게 알림신호를 보낸다. 알림 신호를 전달받은 시술자는 상기 임플란트 픽스츄어 주변의 잇몸의 상태를 육안으로 측정하여 시술을 종료하거나 추가적인 시술 여부를 판단할 수 있다.

[0047] 이상으로 본 발명의 특정한 부분을 상세히 기술하였는 바, 당업계의 통상의 지식을 가진 자에게 있어서 이러한 구체적인 기술은 단지 바람직한 구현 예일 뿐이며, 이에 본 발명의 범위가 제한되는 것이 아닌 점은 명백하다. 따라서, 본 발명의 실질적인 범위는 첨부된 청구항과 그의 등가물에 의하여 정의된다고 할 것이다.

도면의 간단한 설명

[0048] 도 1은 본 발명에 따른 치과 임플란트 픽스츄어 제거용 디바이스의 구성을 설명하기 위한 도면이다.

[0049] 도 2는 본 발명의 히터부 및 온도센서부가 치과 임플란트 픽스츄어와 결합한 상태에서의 단면도이다.

[0050] 도 3 내지 도 6은 본 발명의 히터부와 온도센서부가 작동하여 치과 임플란트 픽스츄어를 제거하는 과정을 순차적으로 보여주는 도면이다.

[0051] 도 7은 본 발명에 따른 치과 임플란트 픽스츄어 제거용 디바이스의 작동 구조를 설명하기 위한 블록도이다.

[0052] <도면의 주요부분에 대한 부호의 설명>

[0053] 10 : 잇몸

[0054] 20 : 치과 임플란트 픽스츄어

[0055] 30 : 치과 임플란트 픽스츄어 내부

[0056] 40 : 매질체

[0057] 50 : 골괴사 부분

[0058] 60 : 핸드피스

[0059] 110 : 히터부

[0060] 210 : 온도 센서부

[0061] 220 : 제어부

[0062] 230 : 입력부

[0063] 240 : 알림부

도면

도면1

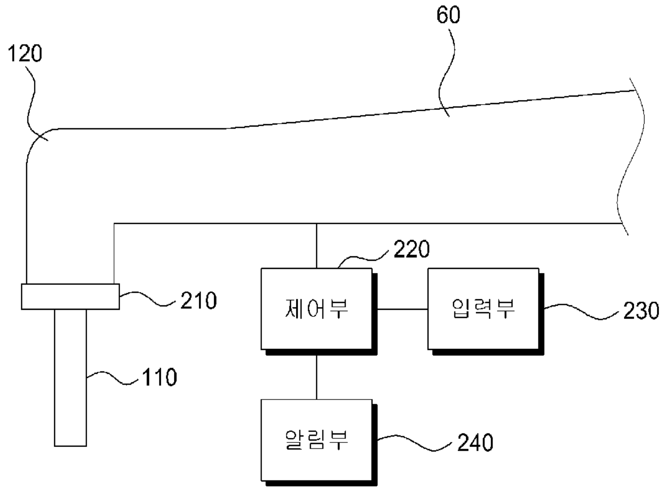

도면2

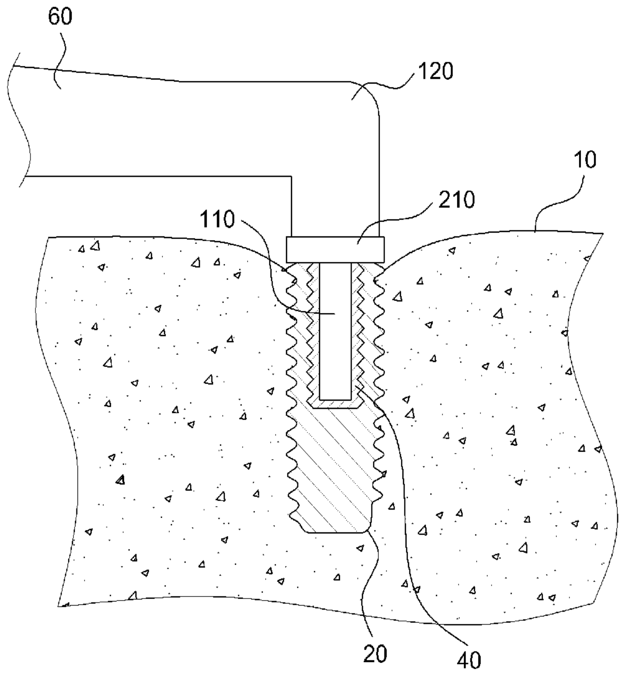

도면3

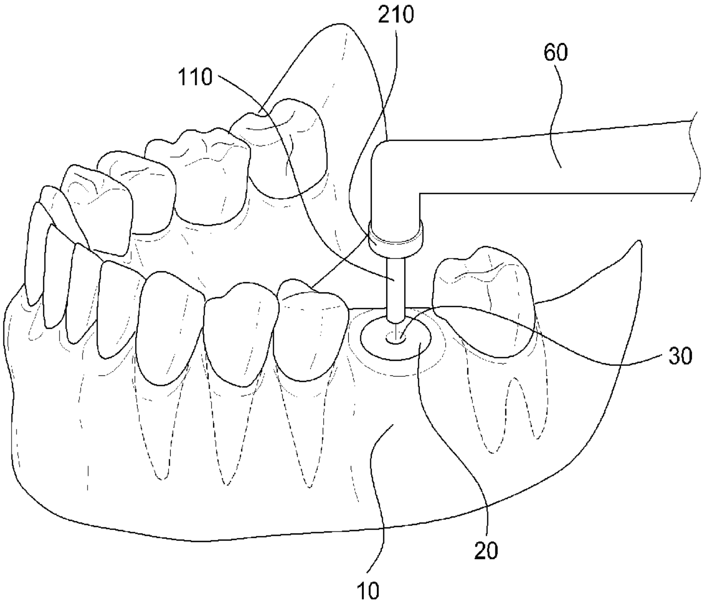

도면4

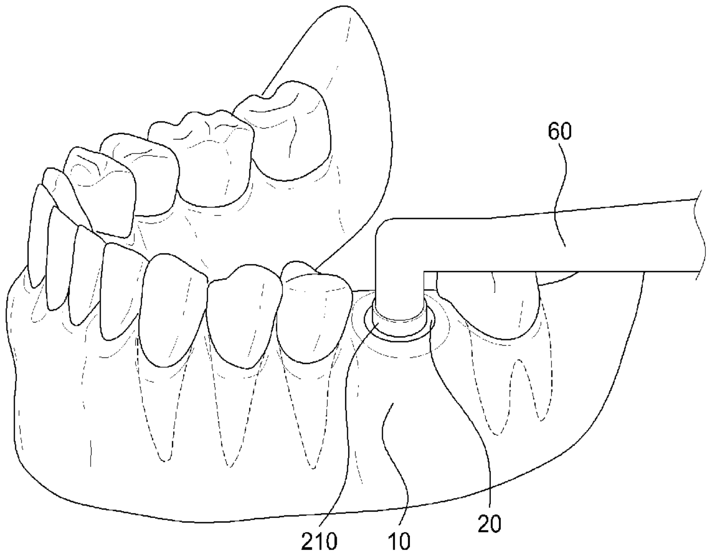

도면5

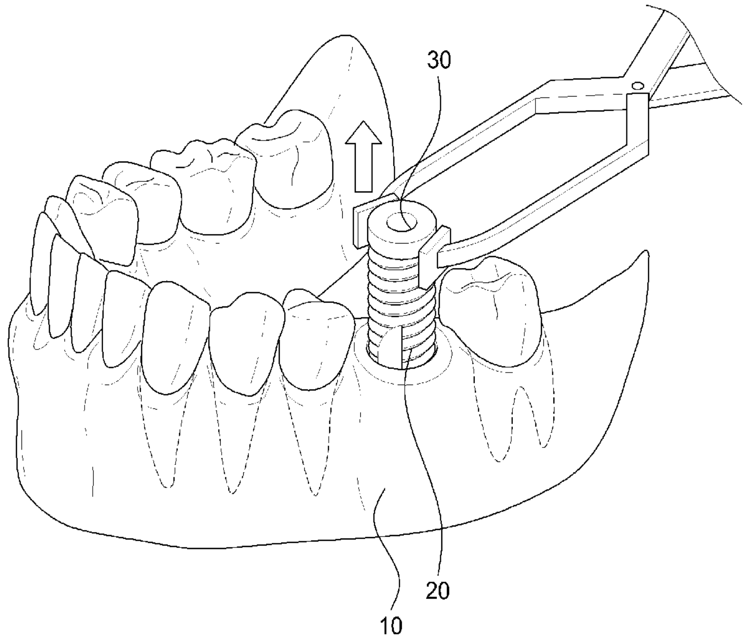

도면6

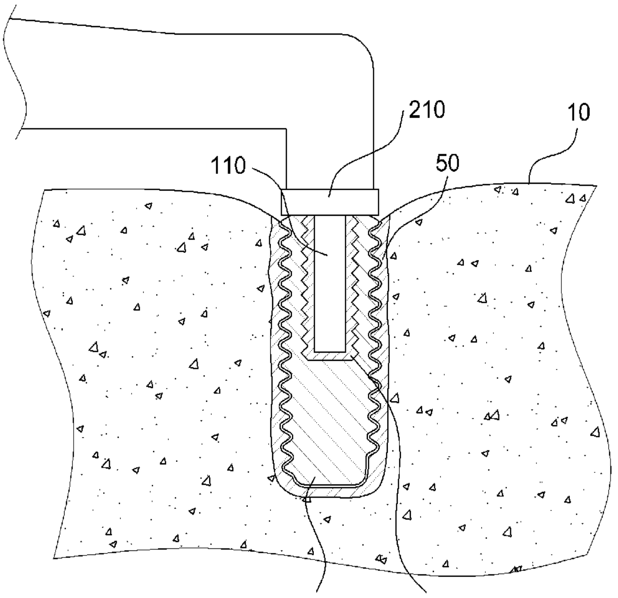

도면7

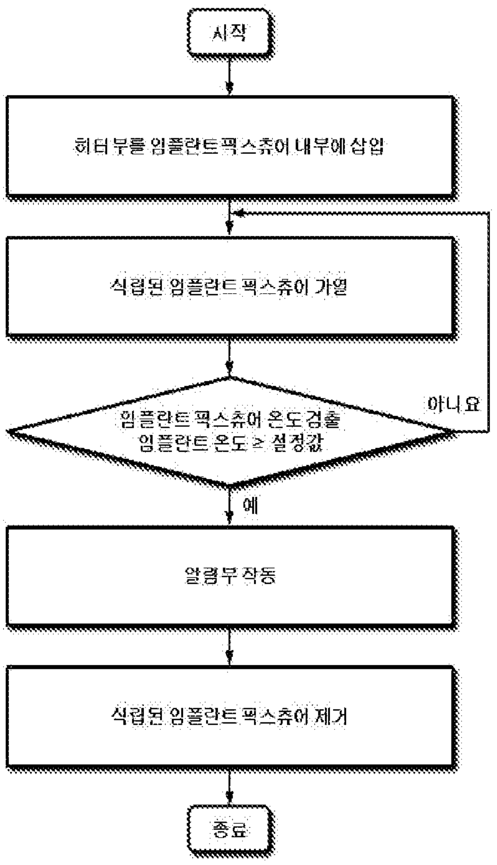
시작
히터부를 임플란트픽스츄어 내부에 삽입
식립된 임플란트픽스츄어 가열
임플란트픽스츄어 온도검출
임플란트 온도 ≥ 설정값
아니요
예
알람부 작동
식립된 임플란트픽스츄어 제거
종료
