# Supplementary material for: Reversal of Osseointegration as a Novel Perspective for the Removal of Failed Dental Implants: A Review of Five Patented Methods
Source: Materials (Basel). 2021 Dec 17;14(24):7829. doi: 10.3390/ma14247829 (PMC8707383; doi:10.3390/ma14247829)
Supplement: Supplementary file 1 [file materials-14-07829-s001.zip › P4 (US) Implaout, Denmark, Petersen.pdf]

US 20140248581A1

(19) United States
(12) Patent Application Publication
(10) Pub. No.: US 2014/0248581 A1
(43) Pub. Date: Sep. 4, 2014
Petersen et al.

(54) DEVICE FOR LOOSENING, INSERTION AND REMOVAL OF DENTAL IMPLANTS

(71) Applicant: **Implaout APS**, Aarhus C (DK)

(72) Inventors: **Jesper Øland Petersen**, Kobenhavn O (DK); **Paolo Maria Cattaneo**, Arhus C (DK)

(21) Appl. No.: **14/349,829**

(22) PCT Filed: **Oct. 5, 2012**

(86) PCT No.: **PCT/DK2012/050379**

§ 371 (c)(1), (2), (4) Date: **Apr. 4, 2014**

(30) **Foreign Application Priority Data**

Oct. 7, 2011 (EP) 11184292.8

Publication Classification

(51) **Int. Cl.**
A61C 1/07 (2006.01)
A61C 8/00 (2006.01)

(52) **U.S. Cl.**
CPC *A61C 1/07* (2013.01); *A61C 8/0089* (2013.01)
USPC **433/119**; 433/167

(57) **ABSTRACT**

A device for inserting a threaded dental implant (I) in a patients jawbone as well as for loosening and removing a threaded dental implant (I) mounted and possibly osseointegrated in the alveolus jaw-bone of a patient. An ultrasonic vibration actuator, e.g. a piezoelectric transducer, generates ultrasonic vibrations with a frequency within the range 20-50 kHz, preferably within 24-36 kHz, and preferably in a direction around the longitudinal axis of the dental implant (I). The actuator, being a handpiece (H) structurally connected with a handpiece head (HD). A fitting-means (F), e.g. a socket, is positioned in the head (HD) of the handpiece (H), the head (HD) being arranged for entering the patient's mouth. The fitting-means (F) is shaped to provide a rigid engagement directly with the dental implant (II, I2) or indirectly by an adaptor (AD1, AD2) being rigidly tightened on top of the dental implant (II, I2) platform. The fitting-means (F) thus transmits ultrasonic vibrations to the dental implant (II, I2) for either inserting a dental implant or alternatively loosening the dental implant from the jaw bone.

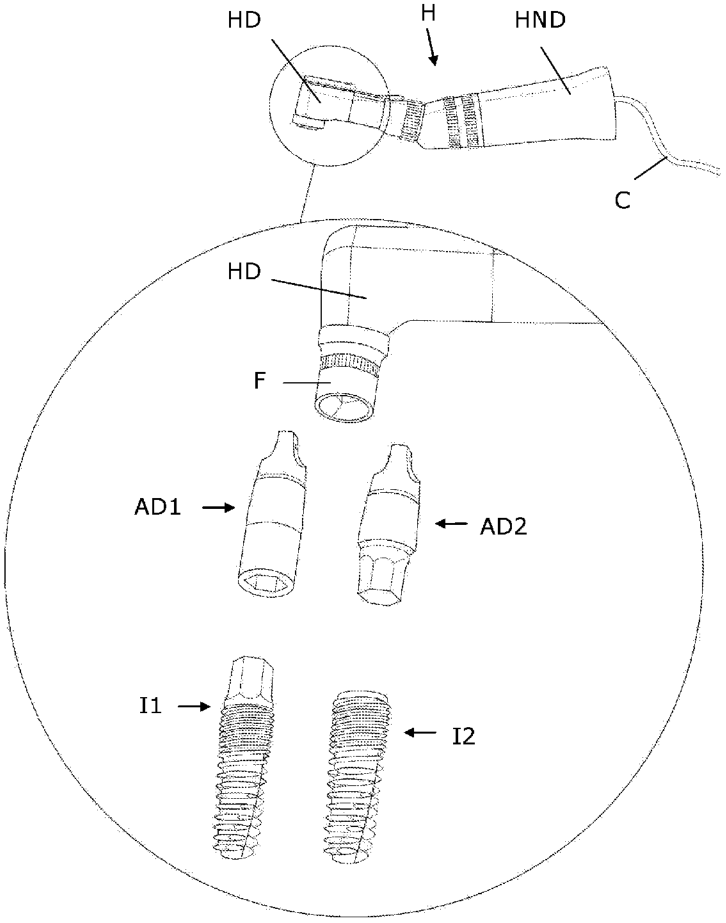

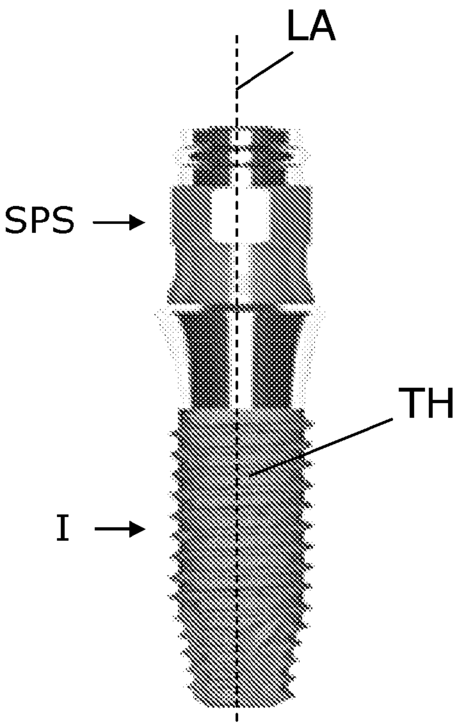

FIG. 1a
Prior art

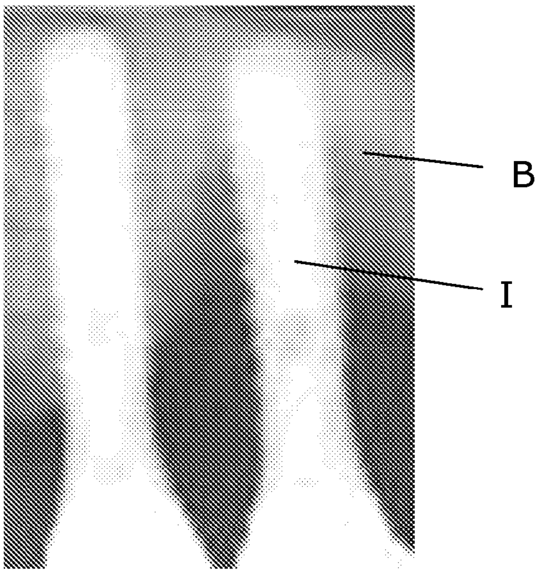

FIG. 1b
Prior art

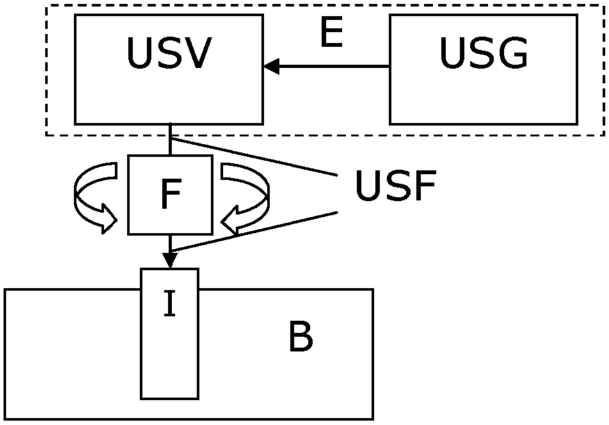

FIG. 2

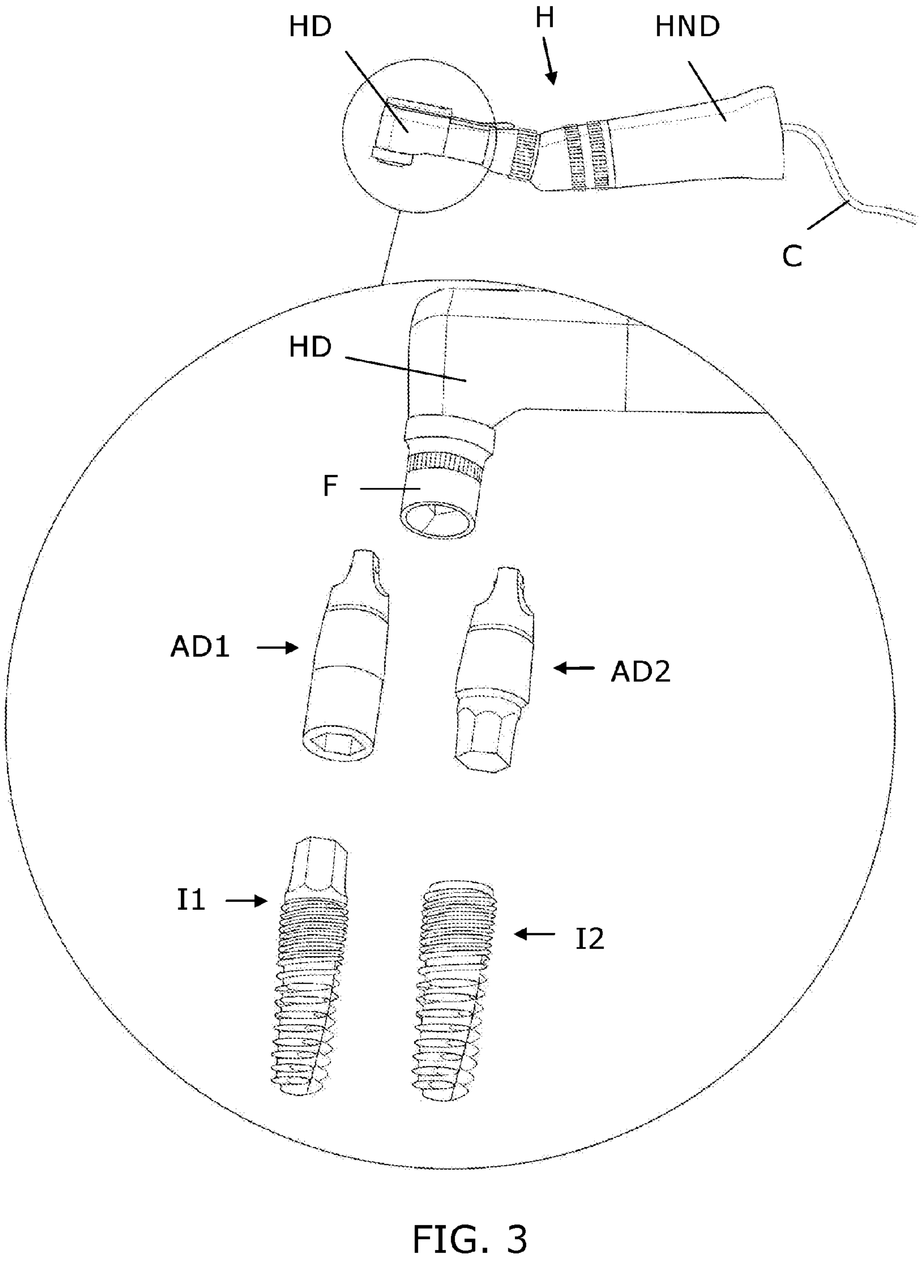

FIG. 3

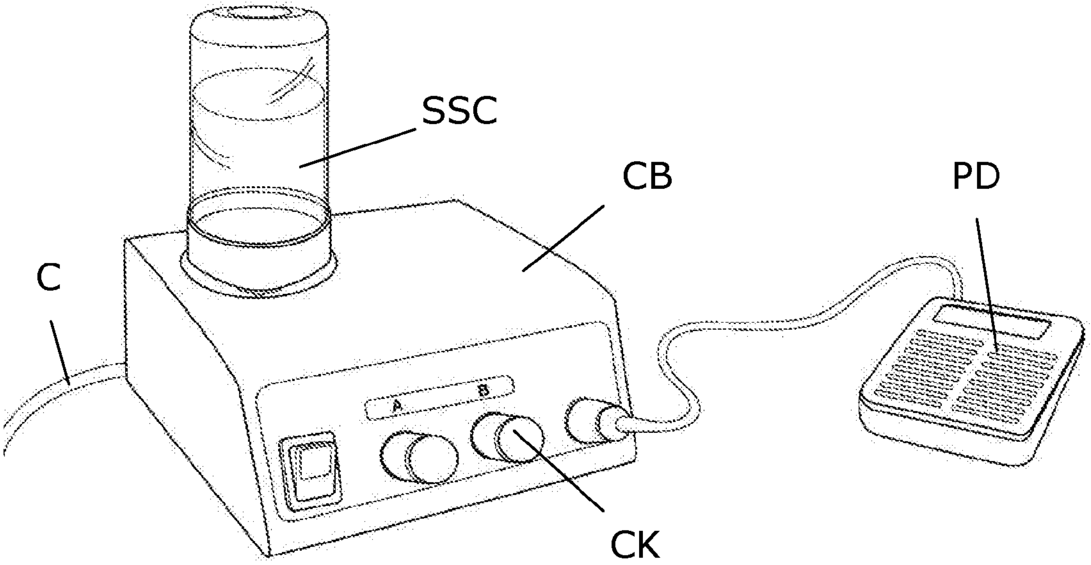

FIG. 4

DEVICE FOR LOOSENING, INSERTION AND REMOVAL OF DENTAL IMPLANTS

FIELD OF THE INVENTION

[0001] The invention relates to the field of medical devices, more specifically dental equipment. The invention provides a device and a method for friction-reducing and bone-saving insertion as well as atraumatic loosening and removal of a dental implant in the jaw-bone of a patient.

BACKGROUND OF THE INVENTION

[0002] Dental implants are "root substitutes" surgically placed in the alveolus jaw-bone to provide anchorage of dental supra-structures such as dental crowns (one dental implant) or bridgework (needs two or more dental implants). In other cases dental implants are placed to support metal bars, bullets, locators or other components for the retention of removable prostheses. Dental implants are typically made of titanium-alloy most often produced in lengths between 6 and 15 mm and in diameters between 3 and 6 mm. By far, the vast majorities of contemporary dental implants are characterized by an outer thread. In respect to the long-axis dental implants are either cylindrical or have a so-called root-form being slightly conical. When a dental implant is cut perpendicular to its long-axis it has a circular cross-section.

[0003] Prior to surgical insertion of a dental implant a cylindrical hole (pilot hole) is first made in the alveolus bone during a step-wise drilling procedure, where the pilot hole is progressively enlarged. With the purpose to minimize the amount of bone being removed during this step-wise drilling procedure, a sequential enlargement of the pilot hole is often achieved by using non-cutting bone-expanders (wedge-principle). This procedure will spare bone as well as improve primary stability of the dental implant after insertion. When the pilot hole in the alveolus jaw-bone has been prepared the dental implant is inserted/screwed in during a clock-wise movement. The diameter of the pilot hole is made slightly narrower than the diameter of the dental implant per se as a threaded dental implant is partly self-tapping. This means that the dental implant is screwed into the alveolus jaw-bone with some resistance that will not only depend on the alveolus jaw-bone quality but also by the difference in diameters of the dental implant and the pilot hole in the alveolus jaw-bone. This modus operandi has shown to give the dental implant a good primary stability and therefore good retention immediately after placement. Under optimal conditions all threads of the dental implant will be sitting below the alveolus jaw-bone ridge.

[0004] After insertion, dental implants are supposed to heal into the alveolus jaw-bone by the principle of "osseointegration", which means that the bone surrounding the dental implant will gradually remodel and grow into a tight connection with the surface of the dental implant. Contemporary dental implants most often have a rough textured surface which increases the overall implant-to-bone contact, thus increasing the bone-to-implant connection once healed.

[0005] The success rate of dental implants is quite high (more than 90% heals in). However, in some cases, dental implants need to be removed for one reason or another. This can be needed either immediately in relation to the surgical insertion process; or delayed after the implant has already healed into the alveolus jaw-bone by the principle of osseointegration.

[0006] Immediate removal of a dental implant sometimes becomes relevant if the dental implant cannot be properly seated in the bony bed during insertion, this as the dental implant unintended becomes friction-locked on its way down into the prepared pilot hole in the jaw-bone. Such locking can occur as a consequence of high friction caused by: dense and rigid alveolus jaw-bone; a too narrow pilot hole; and/or by the building up of bony debris along the threads during the dental implant insertion. Another situation where immediate removal becomes necessary is if the dental implant ends up having an unfavorable angulation after insertion, eventually leading to unintended contact between the dental implant and the neighboring vital structures such as teeth, nerve-structures, or the maxillary sinus.

[0007] When a dental implant has to be removed due to unsuccessful insertion, at present there is a lack of efficient and sufficient removal-tools designed to facilitate unscrewing of the dental implant without destroying it. Thus, the surgeon is faced with the challenge of removing the misplaced dental implant with unsuited utensils (e.g. with tools used for teeth extraction), or alternatively the surgeon has to grind away the jaw bone surrounding the dental implant in order to expose part of it and achieve a better grip on the dental implant so to unloosen it from the surrounding bony structures. The latter procedure normally entails an extensive bone-loss, bearing the additional risk of damaging neighboring vital structures. As a result, the surgeon may often choose to accept the misplaced dental implant from a biological cost-benefit point-of-view.

[0008] Delayed removal of osseointegrated dental implants sometimes become necessary due to chronically infection (peri-implantitis) causing marginal bone-loss and loss of control of infection around the dental implant. Peri-implantitis always develops around the cervical part (collar) of the dental implant pointing towards the oral cavity. Peri-implantitis is developed due to oral bacteria colonizing the dental implant supra-structure (a supra-structure is rigidly mounted on top of the dental implant platform and penetrates through the gingiva into the oral cavity). After this the bacteria can spread down into the gingival pocket surrounding the supra-structure and thus continue all the way down to the bone attaching directly to the dental implant surface. At this stage the bacteria will cause chronically infection followed by bone-loss around the cervical part of the dental implant. This often leads to shrinkage and retraction of the gingiva, contributing to an unfavorable esthetic situation. In addition to bone loss and esthetic complications, the loss of infection control may in itself constitute a severe health-risk affecting the course and pathogenesis of a number of systemic diseases, such as cardiovascular disease, bacterial pneumonia, and diabetes mellitus. For this reason, in those cases where peri-implantitis cannot be arrested with the present treatment options the removal of the dental implant would be the optimal solution from a health-care point of view.

[0009] Even though a dental implant affected with peri-implantitis has lost a significant amount of marginal bone support around its cervical part, it will still be osseointegrated at its apical part, thus still being rigidly anchored to the alveolus jaw-bone. Typically, the only way to loosen such a dental implant is by performing an open surgery. The major problem associated with this operation is that tools strong enough to unscrew the dental implant are not available at present. Moreover, if such a strong tool would actually exist, the dental implant removal would need that much force and

torque that it would likely lead to a major damage of the alveolar bone around the dental implant. This damage would not just be limited to the strong cortical bone-to-dental implant connection (as preferred) but would most probably lead to the disruption of the fragile trabecular bone in a radius of about 2-3 millimeters from the surface of the dental implant. This would cause an unintended and unfavorable bone loss with the creation of a crater-like void including further possible drawbacks like having uncontrolled damage to the above-mentioned neighboring vital structures (bone, teeth, and nerves). Thus, at present, dental implants have to be removed during open flap surgery, where the overlying mucous flap is raised after which the remaining alveolus jaw-bone surrounding the dental implant must be removed gradually by an extensive grinding procedure (e.g. by the use of a trephine bur). Even though this procedure can be performed in a relatively controlled manner it still causes unfavorable loss of alveolus jaw-bone and in addition bears a considerable risk of over-heating and/or inducing direct trauma to the neighboring vital structures.

[0010] In conclusion, there remains a need to find a better solution for inserting as well as for removing dental implants safely and less traumatically than is possible today. An apparatus that would be able to decrease the loss of alveolus jaw-bone during dental implant insertion as well as to facilitate dental implant insertion seems needed. At the same time a tool seems needed that would enable easy, rapid, non-traumatic, and non-invasive removal of a dental implant.

[0011] U.S. Pat. No. 5,330,481 describes implantation and extraction of osteal prostheses employing ultrasonic energy source to be used by orthopedic surgeons to provide a vibration along the long axis of the osteal prosthesis. Further, it is described that the method can be used for other human implants as well. However, U.S. Pat. No. 5,330,481 does not specifically address the problem of loosening an osseointegrated threaded dental implant.

[0012] Thus, the prior art does not provide any method or device for removal of a threaded dental implant without substantial bone damage.

SUMMARY OF THE INVENTION

[0013] Thus, according to the above, it is an object of the present invention to provide method and a device for inserting a dental implant with reduced alveolus bone-loss and with less chance of friction-locking during dental implant insertion. In addition the present invention provides a method and a device for the loosening of a threaded dental implant being friction locked during insertion; and for loosening and removal of an osseointegrated threaded dental implant, which has to be removed, e.g. due to peri-implantitis.

[0014] In a first aspect, the invention provides a device for loosening a dental implant from the alveolus jaw-bone of a patient. In addition, the device may be arranged both for the insertion and removal of a dental implant. The device comprises: a) an ultrasound vibration actuator arranged to generate an ultrasonic vibration with a frequency within the range 20-50 kHz, preferably predominantly within the range 24-36 kHz; b) a handpiece with a head which is arranged for entering the patient's mouth; c) fitting-means positioned in a head end of the handpiece and shaped to provide a substantially rigid engagement either directly with the dental implant platform or indirectly by an intermediate rigid "adaptor" mounted onto the dental implant platform, wherein the fitting-means is connected to the ultrasound vibration actuator so as to transmit the ultrasonic vibration force down into the dental implant. The adaptor too is by principle a supra-structure being attached to the dental implant platform.

[0015] The vibration transferred to the dental implant during insertion will help reducing the friction between the dental implant and the alveolus jaw-bone, thus allowing a reduced diameter of the pilot hole in the alveolus jaw-bone before dental implant insertion. In addition, the vibration produced by the tool being transferred to the dental implant will be suited for loosening/removal of a friction-locked or osseointegrated dental implant from the alveolus jaw-bone. The device would be built up/designed so that it will be highly advantageous for use in a dental practice or the like for easy inserting as well as for easy removing of dental implants.

[0016] The device is thus suitable as a dental instrument e.g. in versions combined with the known function of providing a screwing or unscrewing force, to insert or loosen/remove a dental implant. The device is based on the fact that ultrasonic vibrations can be used to cut bone and at the same time save other tissues, especially in the frequency range 24-36 kHz. The invention device is based on the idea of application of this knowledge to the function of 1) inserting a dental implant by utilizing ultrasonic vibration force to make the implant vibrate during insertion thus decreasing the amount of friction that will occur between the dental implant and the surrounding alveolus jaw-bone, and 2) loosening/removing a dental implant by utilizing the ultrasonic vibration force to cause bone fatigue directly in the bone-to-dental implant interface area. Hereby, the zone of osseointegration is broken with a minimal damage to the bone surrounding the dental implant. The dental implant will thus become loose and can be removed by simply unscrewing it, both avoiding unfavourable loss of alveolus jaw-bone and saving the dental implant from surface destruction or fracture during removal. This procedure only causes a minimum loss of bone, thus increasing the chances of a subsequent successful implantation in the same bone region. Further, in those cases where the dental implant has become friction-locked during insertion, this method may allow reusing the dental implant.

[0017] Compared to invasive methods that are presently used for dental implant removal, such as surgical removal of large volumes of alveolus jaw-bone and/or by trephine-drilling, both causing patient discomfort, possibility of nerve-damage, and increased infection risks, the method of the present invention is minimal invasive. Thus, the device according to the present invention will provide a dental implant loosening/removal method which is much more comfortable for the patient, who will suffer a minimum of risks for additional complications/side-effects caused by the removal process, and further the device will help to improve the prognosis for later implant insertion in the same alveolus jaw-bone region.

[0018] The device of the invention can supplement/modify the existing dental armamentarium suited for screwing or unscrewing dental implants. E.g., in addition to a screwing/unscrewing motor, the handpiece of such existing device can be supplied with a piezoelectric transducer which can generate the ultrasonic vibration. Thus, such technical solution only requires a minimum of modifications of existing equipment. Even though ultrasonic vibration is hitherto unknown in relation to removal of dental implants, dentists are familiar with the use of ultrasound equipment, namely the well-known ultrasonic instruments used for removal of tartar from teeth surfaces.

[0019] In a some embodiments, the ultrasonic vibration actuator and the fitting-means are arranged to apply an ultrasonic vibration force with a rotational component to the dental implant, so as to cause the dental implant to perform an ultrasonic vibration around its longitudinal axis. With ultrasound vibrations limited to predominantly provide rapid clockwise and counter-clockwise movements of the dental implant, the bone is minimally damaged. Ultrasonic vibrations will facilitate dental implant insertion (reduce building up of debris and thus reduce friction), and ultrasonic movements will facilitate loosening of a friction locket dental implant (loosen the debris that has built up and thus reduce friction); and for an osseointegrated dental implant (with peri-implantitis) ultrasonic movements will lead to atraumatic loosening of the dental implant from the surrounding alveolus jaw-bone by fatigue of the alveolus jaw-bone after which the dental implant can be easily unscrewed.

[0020] In other embodiments, the mentioned rotational component of the ultrasonic vibration force is either replaced by or supplemented by other directions. Such other directions may be in the direction of the longitudinal axis of the dental implant, or perpendicular to the longitudinal axis of the dental implant. Depending on the type of implant and other factors, the ultrasound vibration actuator may thus include one or more transducers positioned to transmit such various ultrasonic force directions to the fitting-means. Especially, it may be preferred to be able to select two or more dominant direction components of the ultrasonic vibration force. Alternatively, it may be desired to mix two or more direction components into a random direction pattern.

[0021] In preferred embodiments, the device is arranged to apply a torque to the fitting-means upon activation so as to turn the fitting-means counter-clockwise for unscrewing the dental implant. This force may be applied manually by the operator or the force may be applied by a motor. Additionally or alternatively, the device may be arranged to apply a torque to the fitting-means to turn the fitting-means clockwise for mounting the dental implant.

[0022] The device may comprise a motor connected to the fitting-means upon activation so as to turn the fitting-means clockwise for inserting, and counter-clockwise for unscrewing the dental implant. The motor may be an electric or pneumatically driven motor. In such embodiments, the motor and the ultrasound vibration actuator may be mounted so in relation to the fitting-means that both the unscrewing force and the ultrasonic vibrations are transmitted through the fitting-means. This is advantageous, since this enables direct transmission of energy to the dental implant and simultaneously or subsequently screwing (inserting) or unscrewing the dental implant using only one instrument. Especially, the motor may be arranged to turn the fitting-means both clockwise and counter-clockwise upon selective activation, so as to enable selective screwing or unscrewing of the dental implant. Such embodiment allows one single instrument to be used for both inserting and removing of a dental implant. In a specific embodiment, the ultrasonic vibration actuator and the motor are arranged to function simultaneously, so as to enable application of the ultrasonic force to the dental implant during screwing or unscrewing. The combination of ultrasound vibration with motor driven rotations can facilitate insertion as well as loosening/removal of a dental implant with a minimum of alveolus jaw-bone damage: During dental implant insertion, the resistance between the dental implant and the surrounding alveolus jaw-bone will be reduced when motor driven clockwise rotations is combined with ultrasonic vibrations of the dental implant, thus minimizing the risk of the dental implant being friction locked during insertion. Contrary, during loosening/removal, first the ultrasonic vibrations are used for loosening of the dental implant from the alveolus jaw-bone (fatigue) after which removal of the dental implant is completed by motor driven counter-clockwise unscrewing of the dental implant.

[0023] In preferred embodiments, the applied ultrasonic vibration force is mainly directed clockwise for inserting a dental implant, while the ultrasonic vibration force is mainly applied is a counter-clockwise direction for loosening of the dental implant.

[0024] In contrast to the above mentioned combined screwing/unscrewing and ultrasound vibration device, a specific ultrasonic vibration device embodiment can be desired. E.g. in a battery driven version with the battery placed in the handpiece, thus allowing the user to easily operate the device as a separate tool and selectively use such tool to loosen a dental implant, and then unscrew the implant with another instrument. Such embodiment can be made in a low cost version, since its structure is simpler due to the fact that no combination of ultrasonic vibration forces and screwing/unscrewing forces are required.

[0025] In some embodiments, the ultrasonic vibration actuator is driven by a piezoelectric transducer element or by pneumatics. As mentioned, two or more transducer element may be combined at different positions and different angles of engagement with the fitting-means, so as to provide the desired vibration direction pattern to the dental implant.

[0026] A function of the ultrasonic vibration actuator may be controlled by a processor arranged to at least start and stop the generation of ultrasonic vibration force upon user activation. E.g. the processor generates electric control signals to control the ultrasonic vibration actuator. In some embodiments, the processor is arranged within a separate control box connected to the handpiece by a cord. The control box has a user interface with related user activation means, such as push buttons, turning knobs, foot pedals etc., allowing the user to control function of the device. Thus, with such separate control box having a user interface, the handpiece can be operated effectively, and the function and operation of the device will fit with existing equipment in a dental or surgical clinic.

[0027] Especially, the processor may be arranged to control the ultrasonic vibration actuator so as to vary at least one parameter of the ultrasonic vibration force. This will allow the operator, or an automatic control scheme, to control the ultrasonic vibration force for the optimal loosening or insertion of the dental implant taking into account the actual circumstances. E.g. a number of predefined vibration patterns with different parameter profiles stored in the control box may be selectively activated, allowing the user, or an automated algorithm, to find the most effective vibration pattern in the actual situation. In some cases only a limited amplitude or duration of ultrasonic vibration may be required for loosening the dental implant, and thus the vibrations can be limited to a minimum, thus providing a minimum damage to the bone. In more advanced embodiments, one or more sensors may be placed in connection with the fitting-means so as to sense if the dental implant has been loosened. Output from such sensor(s) may be used as a feedback signal to a control algorithm controlling the ultrasonic vibration force. E.g. a force sensor may sense a clock-wise or counter clock-wise torque provided during insertion or loosening of the dental implant and

provide an output signal accordingly, wherein this output signal is used as input to a control algorithm controlling a function of the ultrasonic vibration actuator, such as an algorithm activating the ultrasonic vibration only when the torque exceeds a predefined limit, e.g. 20 Ncm, 30 Ncm, or 40 Ncm.

[0028] The processor arranged to control at least one parameter of the ultrasonic vibration force may be arranged to vary the ultrasonic vibration force with respect to at least one of: amplitude, frequency content, and direction. Especially, the processor may be arranged to vary an amplitude of the ultrasonic vibration force, e.g. within a vibration displacement range of 10 μm to 500 μm, such as within 30 μm to 300 μm, such as within 60 μm to 210 μm. A turning knob may be provided to allow the user to adjust the vibration displacement to a desired level. More specifically, the processor may be arranged to temporary vary the amplitude of the ultrasonic vibration force, e.g. providing vibrations modulated in amplitude with a fixed modulation frequency, or providing impulsive vibrations, or providing random amplitude vibrations.

[0029] In some embodiments, the processor is arranged to vary a frequency content of the ultrasonic vibration force, such as varying a frequency of a single frequency vibration at least within 24-36 kHz, such as selecting between pluralities of vibration profiles with different frequency spectra. A turning knob may be provided to allow the user to adjust a frequency of a single frequency or narrow band vibration to a desired frequency within a limited frequency range, at least covering 24-36 kHz, e.g. 20-50 kHz. Different frequencies may provide the most effective loosening depending on the actual circumstances and structure of both alveolus jaw-bone and surface of the dental implant.

[0030] The device may be supplemented by a plurality of removable adaptors (supra-structures) which each have a first end shaped to provide a rigid fit to the fitting-means of the working handpiece and with a second end shaped to provide a rigid fit to respective different shapes of associated types of dental implants. This allows the device to be manufactured with one fixed fitting-means arranged for engagement with the first end of the adaptors, and by means of color codes or the like, such adaptors will allow the device to be used with different types of dental implants. Preferably, the adaptors are made of a material capable of effective transmission of the ultrasonic vibration force. Especially, the first end of the adaptors may have a locking mechanism, such as a resilient click-function mechanism, so as to lock the adaptors to the fitting means of the working handpiece during use, and still allow the user to remove the adaptor when needed.

[0031] In a second aspect, the invention provides a method for loosening a dental implant from the jaw-bone of a patient, such as for inserting or removing the dental implant, the method comprising the generation of an ultrasonic vibration force with a frequency within the range 20-50 kHz, preferably predominantly within the range 24-36 kHz, transmitting the ultrasonic vibration force to the dental implant by a fitting-means being rigidly connected either directly to the dental implant platform or to an intermediate adaptor tightly fixed to the dental implant platform with a locking-screw. Whatever type of rigid connection is used, the ultrasonic vibration will be transferred directly to the dental implant. This will cause loosening of the dental implant from the alveolus jaw-bone by fatigue. After loosening of the dental implant it can be gently removed by applying an unscrewing motor driven force.

[0032] Preferred embodiments comprises applying a torque to the fitting-means upon activation so as to turn the fitting-means counter-clockwise for unscrewing the dental implant simultaneous with transferring the ultrasonic vibration to the dental implant. This unscrewing torque may be applied manually by an operator, or it may be applied by a motor.

[0033] It is appreciated that the mentioned embodiments may in any way be combined with each other. Further, the same embodiments and advantages mentioned for the first aspect apply as well for the second aspect. In both aspects, dental implant insertions as well as dental implant removals are preferred uses of the device and method.

BRIEF DESCRIPTION OF DRAWINGS

[0034] In the following, the invention will be described in more details by referring to embodiments illustrated in the accompanying drawings, of which

[0035] FIG. **1***a* shows a photo of a dental implant mounted with a rigid adaptor,

[0036] FIG. **1***b* shows an x-ray image of two dental implants situated in the alveolus jaw-bone, the left with healthy bone conditions, the right with pronounced peri-implantitis,

[0037] FIG. **2** shows a block diagram of a device embodiment,

[0038] FIG. **3** illustrates a sketch of a handpiece embodiment as well as two types of adaptors/supra-structures or designed to fit two different types of dental implants (dependent on type of dental implant platform), and

[0039] FIG. **4** illustrates a sketch of a control box embodiment for controlling the handpiece of FIG. **3**.

DETAILED DESCRIPTION OF THE INVENTION

[0040] FIG. **1***a* shows a photo of a typical dental implant I with a supra-structure SPS mounted thereon. A dental implant I is in principle a bone-screw supplied with an inner wind for mounting of the supra-structure SPS. A dental implant I is parallel or eventually slightly tapered in its longitudinal axis LA. When sectioned perpendicular to its longitudinal axis LA, the dental implant I is circular in cross section. The vast majority of dental implants I are characterized by an outer-surface thread TH.

[0041] Moreover, an internal wind is present, stretching from the dental implant platform (cervical part) down into the inner central part of the dental implant I along its longitudinal axis LA. The purpose of this inner wind is to allow the mounting of the supra-structure SPS onto the dental implant platform, e.g. a so-called "abutment" to support a cemented dental crown. In order to avoid rotation of the mounted supra-structure SPS in relation to the dental implant I, the platform of the dental implant I is made with an anti-rotation design. In some dental implants I the anti-rotation design is made externally, typically as a hexagon then being part of the dental implant platform, whereas other dental implants I are designed with an anti-rotation platform made internally then reaching from the cervical part into the inner aspect of the dental implant I in its apical direction. In the latter, the internal wind for mounting the supra-structure SPS is positioned underneath and in continuation with the internal anti-rotational platform. Independently from the fact that the design of the dental implant platform is external or internal, the supra-structure SPS to be mounted on top of the dental implant I is customized to match the anti-rotation platform exactly.

[0042] With regard to the interface between the dental implant I and the surrounding alveolar jaw-bone, the outer surface of the successfully healed dental implant I will be covered completely by alveolus jaw-bone being tightly attached to the threaded external surface TH of the dental implant I. The strong bone-to-implant attachment is referred to as "osseointegration". The dense cortical bone that covers the surface of the dental implant I is relatively narrow compared to the wider zone of trabecular bone next to the cortical bone.

[0043] FIG. **1***b* shows an x-ray image of two dental implants, white color, mounted and surrounded by bone structure B, i.e. the grey colored zone. The implant to the left is surrounded tightly by the bone B structure, as intended. However, the implant I to the right is seen to be only partly surrounded by the bone B, as it appears that a loss of bone B has occurred in the area around the implant I. Such implant I should normally be removed, however even though it appears that the implant I is not totally osseointegrated, it is normally impossible to remove by means of simple unscrewing.

[0044] The device and method according to the invention facilitates such removal without complicated surgery, without the patient suffering from discomfort, and without further loss of alveolus jaw-bone. The device embodiments described in the following are developed both to facilitate dental implant insertion as well as to enable atraumatic dental implant removal.

[0045] FIG. **2** illustrates a diagram of central elements of a device embodiment. The device comprises an ultrasonic vibration actuator USV based on one or more piezoelectric transducers supplied by an electric signal E from an ultrasound signal generator USG. In the specific embodiment, both the actuator USV and the generator USG are placed inside a handpiece housing, indicated by a dashed line, however it is to be understood that the generator USG may be placed outside the handpiece.

[0046] The ultrasonic vibration actuator USV can generate an ultrasonic vibration force USF with dominating amplitude in the frequency range 24-36 kHz, e.g. a single frequency vibration or a narrow band vibration. The actuator USV is connected to a fitting-means F externally accessible and shaped to engage either directly with the top (platform) of a dental implant I or indirectly with an intermediate suprastructure SPS (adaptor) rigidly connected to the dental implant platform, so as to transmit the ultrasonic vibration force USF to the dental implant I. Of course, the part of the handpiece holding the fitting-means F should have dimensions allowing insertion into a patients mouth so as to allow engagement between the fitting-means F and the dental implant I, preferably the handpiece is shaped and sized so as to allow such engagement (contra-angle handpiece) to allow access to dental implants at all quadrants of a patient's mouth.

[0047] In the sketch, the dental implant I is positioned and osseointegrated in the alveolus jaw-bone B of a patient. By application of the ultrasonic vibration force USF, preferably predominantly a rotational force as indicated by the arrows, the dental implant I will be loosened from the bone B due to fatigue breakage of the bone structure immediately surrounding the dental implant I. This facilitates removal of the implant I by unscrewing. The unscrewing force may be built into the same tool as the ultrasonic device, however if preferred, the ultrasonic device may be a dedicated loosening device, while another instrument may be used for unscrewing.

[0048] FIG. **3** shows a sketch of a dental instrument handpiece H embodiment comprising a handpiece handle HND structurally connected to the head HD of the handpiece. The magnified view illustrates the head of the handpiece HD mounted with a fitting-means F. The fitting-means F will fit exactly onto different kind of supra-structures, for this invention called adaptors AD**1**, AD**2**. The adaptors AD**1**, AD**2** are different in design as to match various platforms of dental implants I**1** and I**2**.

[0049] Thus, implant I**1** is illustrated with an external platform whereas implant I**2** is illustrated with an internal platform. The adaptor type AD**1** is rigidly fixed on top of the implant type I**1**, and the adaptor type AD**2** is rigidly fixed into the dental implant I**2**, so as to allow ultrasonic forces generated in the head HD to be directly transmitted to the dental implant I**1**, I**2**. The rigid fixation between the dental implant I and the adaptor AD**1**, AD**2** is provided with a locking-screw running through the central part of the adaptor AD**1**, AD**2** into the internal wind of the dental implant I**1**, I**2** (not illustrated in the drawing).

[0050] In the opposite end of the handle HND, a cord C serves to connect the handpiece H to a control box (see FIG. **4**). Preferably, the handpiece H contains an ultrasonic vibration actuator (not visible) which is electrically powered and controlled via the cord C, e.g. the actuator includes one or more piezoelectric transducers. Further, the handpiece H preferably also contains a motor (not visible) arranged to provide a screwing and unscrewing force to the fitting-means F, so as to allow rotations of the handpiece head HD to perform a complete insertion and removal of a dental implant without the use of other instruments. The motor may be electrically or pneumatically driven, and the cord C thus also serves to provide the necessary electric and/or pneumatic connection to supply and control the motor. Preferably, the handpiece H is crafted in accordance with existing principles for dental equipment, and thus at least the head portion HD may be made of metal.

[0051] FIG. **4** shows an example of a control box CB for use with the handpiece H of FIG. **3**. A processor (not visible) and associated electronic circuits are contained inside a box which on its exterior has a user interface in the form of control knobs CK for adjustment of the mode of operation and/or for control of parameters related specifically to control of the ultrasonic vibration force, e.g. with respect to amplitude, frequency, temporal modulation and the like. At least one of the control knobs CK is preferably dedicated to switch on/off the ultrasonic vibration. However, the processor may run an automatic algorithm that, based on an input from a sensor in the handpiece H, allows adjustment of the ultrasonic vibration force in order to vary the ultrasonic vibration according to the actual conditions during insertion or removal of a dental implant I**1**, I**2**. A wire connected pedal PD also forms part of the user interface. A sterile saline solution container SSC is mounted on top of the control box CB allowing a saline solution filled therein to be transported via a tube in the cord C to the handpiece H.

[0052] In the following, further explanations are given, referring to the device embodiment shown on FIGS. **3** and **4**. The device preferably has three distinct operating modes which are delivered by the handpiece H:

[0053] 1) Ultrasonic vibrations are generated in either a clockwise or a counter-clockwise direction in respect to the handpiece head HD;

[0054] 2) Motor driven rotations are generated likewise in either a clock-wise or a counter-clockwise direction in respect to the handpiece head HD;

[0055] 3) Ultrasonic vibrations are combined with the motor driven rotations, again in a clock-wise or a counter-clockwise direction in respect to the handpiece head HD.

[0056] By connecting the fitting-means F of the handpiece head HD with the adaptor AD**1**, AD**2** being rigidly fixed on the dental implant I**1**, I**2**, the dental implant I**1**, I**2** will vibrate with the same frequency as the fitting-means F engaged in the handpiece head HD. Thus, the ultrasonic vibrations generated by the actuator in the handpiece H are transferred to the dental implant I**1**, I**2**. Preferably, all parts in this chain of transmission are made of materials rigidly connected at ultrasonic frequencies below 50 kHz, so as to provide a vibration transmission without severe loss of energy. Further, the interconnections should preferably be made also to avoid severe losses at ultrasonic frequencies.

[0057] The ultrasonic vibrations and motor rotations are transferred from the fitting-means F of the handpiece H to the dental implant I**1**, I**2** in the following manner: An adaptor AD**1**, AD**2** is rigidly fixed on top of the dental implant I**1**, I**2** platform. To the left on FIG. **3**, an adaptor AD**1** that will match a dental implant I**1** having an external antirotation platform is shown, while an adaptor AD**2** that will match with a dental implant I**2** having an internal antirotation platform is depicted on the right. These drawings are just made for exemplification and they do not reflect the real antirotation platforms which may have a large variety of shapes.

[0058] Independently from the shape of the adaptor AD**1**, AD**2** on the end that needs to match a specific dental implant I**1**, I**2** platform, the opposite end of the adaptor AD**1**, AD**2** will always have the same shape and dimension, in order to match the connecting fitting-means F of the handpiece head HD, preferably using a click-on principle such as known from a "bit". The chain of connections all the way from: 1) the handpiece head HD to; 2) the fitting-means F to; 3) the adaptor AD**1**, AD**2** to; 4) the implant I**1**, I**2** are preferably rigid so that the ultrasonic vibrations and/or the rotational movement can be effectively transferred from the handpiece H via the handpiece head HD to the dental implant I**1**, I**2** with a minimal loss of energy.

[0059] Dependent on the task that has to be performed, the handpiece H can work both in a clockwise mode and in a counter-clockwise mode. For removal of a dental implant, the device is initially set to operating mode 1 (counter-clockwise directed ultrasonic vibrations) for atraumatic loosening of the dental implant I**1**, I**2**. After loosening, the device is set to operating mode 2 for motor driven counter-clockwise directed unscrewing of the dental implant I**1**, I**2**. For insertion of a dental implant I**1**, I**2**, the device can either be set to mode 1, i.e. clockwise directed ultrasonic vibrations, or mode 2, i.e. motor driven clockwise directed rotations, or mode 3, i.e. motor driven rotations simultaneously with clockwise ultrasonic vibrations in a clockwise direction.

[0060] The modus operandi of the dental handpiece H is controlled by the user interface of the control box CB. In one embodiment, this user interface comprises a set of regulators CK to control the motor driven torque, the power of the ultrasonic vibrations as well as their vibration frequency, which is preferably in the range 24-36 kHz. Further, a control knob CK is used to control whether the direction should be clockwise or counter-clockwise. Moreover, a switch is present to control a water pump in order to change the flow rate of the saline solution from the container SSC. This solution is needed for irrigation and for cooling of the area of intervention. The activation of the handpiece H is controlled by means of a foot pedal PD and/or directly by an operator on the handpiece H itself.

[0061] For implant removal, one of the advantages of using ultrasonic vibrations to disrupt the bone-to-implant connection is that such an apparatus will only require relatively little power to operate. Indeed, this type of apparatus requires near zero force in the axial direction, and a very low torque force will be necessary. As mentioned above, one of the properties of the apparatus is that it produces ultrasonic vibrations that are transferred to the seated dental implant making the dental implant vibrating. To work properly, the vibrations at the dental implant level must exceed those vibrations that will inevitably arise in the alveolus bone surrounding the dental implant. The apparatus is designed to make the dental implant vibrate with a frequency preferably in the range 24-36 kHz which is known to be sufficient to cut bone safely during Piezo-surgery, see e.g. EP 2 066 255 B1 by PIEZOSURGERY S.R.L. By making the dental implant vibrating within this range only mineralized bone tissue will be affected while neurovascular and other soft tissues will not be damaged. It has been previously proven that such vital structures will only be damaged at frequencies above 50 kHz. By using saline irrigation during function the temperature of the alveolus jaw-bone will preferably be kept below 47° to prevent cell damage and death in the area.

[0062] The use of different types of adaptors AD**1**, AD**2** is necessary in order to closely match the different dental implant platforms I**1**, I**2** that differ substantially from one dental implant type to another. The rigid fixation between the adaptor AD**1**, AD**2** and the dental implant I**1**, I**2** is secured by a locking-screw going through the long-axis central part of the adaptor AD**1**, AD**2** into the internal wind/thread of the dental implant. By the establishment of a strong and rigid connection the actuator will be able to transfer the ultrasonic vibrations and the rotation through the handpiece head HD down into the dental implant I**1**, I**2**. Through the use of specific color codes and/or identification codes the selection of the suited adaptor AD**1**, AD**2** will be essentially error-proof. Unintended loosening and unintended rotation of the adaptor AD**1**, AD**2** during function is avoided by using that specific adaptor especially customized to closely match with the antirotation platform of that specific type of dental implant I**1**, I**2**. The adaptor AD**1**, AD**2** is preferably produced in a material that will minimize the risk of the adaptor AD**1**, AD**2** destroying the dental implant I**1**, I**2** platform, i.e. machined from stainless steel, titanium, etc. but it may also be sintered, cast, etc. to the appropriate dimensions and using different materials.

[0063] The advantage of using the device during dental implant insertion is that it will reduce the risk of friction locking of the dental implant before it is properly seated in the alveolus jaw-bone. This is avoided due to the vibrations added to the dental implant which will prevent bone debris building up during the clock-wise self-tapping insertion of the dental implant. This means that the pilot-hole made in the alveolus bone prior to dental implant insertion can be made with a smaller diameter than is recommended in today's protocols for dental implant placement. By this, primary stability of the dental implant just after insertion will be

increased reducing the healing period needed before loading the dental implant with a supra-structure, e.g. a dental crown.

[0064] If a dental implant should anyway become friction-locked during insertion, the invention secures safe and easy loosening and unwinding of the dental implant simply by changing the clock-wise directed vibrations and motor rotation into counter-clockwise directed vibrations followed by counter-clockwise motor rotations of the handpiece after loosening of the dental implant. In detail, the friction-locked dental implant will initially be exposed to vibrations only. Not until after the dental implant has become loose from the vibrations the motor driven counter-clockwise rotations will take over to gently unscrew the dental implant, thus applying only minor forces and moments. After dental implant removal the drill-hole in the alveolus bone can then be rinsed with sterile saline, and, if necessary, the diameter of the bony hole can be slightly increased, e.g. with drills or bone-expanders, before the re-insertion of the dental implant will take place. As the dental implant has been removed in an atraumatic way it will still be fully intact and suited for re-insertion.

[0065] The need of removing a misplaced dental implants during the insertion procedure is rather common, especially in cases where the dental implant has become friction-locked during unsuccessful insertion. The device will transfer vibrations to the dental implant in a counter-clockwise direction and only the debris directly in the bone-to-dental implant contact zone will be disrupted thus preserving the alveolus jaw-bone. After loosening followed by unscrewing of the dental implant the wound can be left for healing. Alternatively, if the amount of alveolus jaw-bone available is still sufficient, the direction of the drill-hole can be slightly altered after which the dental implant can be properly re-inserted.

[0066] When an osseointegrated dental implant has to be removed due to loss of infection control (peri-implantitis) or malposition, ultrasonic vibrations are applied to the dental implant through the adaptors, as already described, preferably in a counter-clockwise direction. The dental implant is set to vibrate with a frequency in the range 24-36 KHz. This vibration would be able to disrupt the alveolus bone-to-dental implant contact by the principle of fatigue-failure of the bone directly in contact with the dental implant causing only minor damages to the alveolar bone at a short distance from the implant and practically no damages in the surrounding soft tissues like gingiva, blood vessels, and nerves. With this modus operandi the dental implant will cut itself loose from the alveolus jaw-bone, thus rendering it possible to perform the implant removal with a flapless (non-invasive) procedure. After loosening, and the subsequently unscrewing of the dental implant, a new dental implant can eventually be immediately re-inserted, taking into consideration the new marginal alveolus bone level so to achieve a better esthetical and functional result. Alternatively, the wound in the alveolus bone can be augmented with a bone-transplant or just left for healing for the necessary period after which eventually a new dental implant can be placed.

[0067] In the following, results of a preliminary experiment to test the device and method according to the invention will be described, namely in the form of a test of loosening and removing embedded or friction-locked dental implants with the use of ultrasonic vibrations combined with a light contra-clockwise rotation torque.

[0068] In the experiments, the Brånemark MarkIII dental implants were used, but it is understood that other types of dental implants could have been used just as well with a similar result. The implants had a diameter of either 3.75 mm or 4.0 mm and they shared the same type of platform.

[0069] Materials used for embedding dental implants were:

1. Plaster: Snow White Plaster No 2, Kerr; a material that after mixing is fluid and allows a dental implant to be embedded; after the hardening process, the plaster gets solid and attaches itself to the windings of the dental implant surface.

2. A bone-like plastic material: Polyurethane foam; a material that after mixing is fluid and allows a dental implant to be embedded, and a material that, after having hardened, is porous and has approximately the same elasticity modulus as human bone. This material is often used as bone substitute for orthopaedic training.

3. Excised fresh bovine tibia bones: for the insertion procedure, pilot holes of 2.7 mm (smaller than what typically used for clinical dental insertion of a 3.75 mm or 4.0 mm dental implant) were drilled into the cortical and cancellous bone. The dental implants were then inserted into the bony pilot hole as deep as possible, just until they became tightly friction-locked and could neither be loosened nor further inserted using the typical existing implant equipment (torque well above 35 N/cm).

4. Hard epoxy-bone cementum: Refobacin® Bone Cement, Poly-methyl acrylate & poly-methyl methacrylate; a material that after mixing is fluid and allows a dental implant to be embedded; after polymerization, this material gets hard and has a Young's modulus higher than vital human bone. Under normal working conditions this material serves as bone cement for fixation of hip prosthesis.

[0070] A Sonopuls HD 2070, (Bandelin electronic, Germany) with a power of 70 W was used as a source for ultrasonic vibrations. This machine is an "Ultrasonic homogenizer" that under normal working conditions is used to emulsify, homogenize, and stir fluids. For the experiments, the Sonopuls was used as a relative powerful source of ultrasonic vibrations. The Sonopuls HD 2070 ultrasonic tool had an ultrasonic generator mounted with a long metallic "lance" that under normal working conditions would go into the fluid that has to be homogenized.

[0071] Cylindrical hollow supra-structures cast in titanium in 3 different lengths (7 mm, 10 mm and 13 mm) were used for the experiments. The different lengths used were designed in order to be able to change the distance between the location of ultrasonic force application and the dental implant platform. The bottom end of the supra-structure was shaped to exactly match the platform of the dental implant. The supra-structure was then rigidly tightened to the dental implant platform with a standard internal stainless steel screw. At the opposite end, a cutting-through hole was made on the lateral aspect of the supra-structure. This hole was made so that the tip of the ultrasonic "lance" could loosely engage during testing: the diameter of the hole was thus large enough to accommodate the ultrasonic lance, still wide enough to allow the lance to vibrate freely onto the hole-wall. The reason to have the hole positioned at the lateral aspect of the cylinder was to be able to apply a counter-clockwise—unscrewing—torque to the supra-structures and thus indirectly to the dental implant (the cylinder was rigidly connected to the dental implant platform) via the ultrasonic lance. Thus, the ultrasonic vibrations applied to the dental implant supra-structure were combined with a slight counter-clockwise unscrewing of the dental implant.

[0072] During all experiment, irrigation with running cold water was applied to reduce the generation of heat as well as to hinder the formation of sparks.

[0073] With the above-described experimental setup it was possible to apply counter-clockwise torque to the dental implant while applying an ultrasonic vibration. In addition, it was possible to apply such combined forces in different distances from the dental platform, this by using different lengths of supra-structures (7 mm, 10 mm and 13 mm).

[0074] A customized titanium supra-structure was mounted and rigidly connected with a stainless steel screw onto the platform of a dental implant. After this, the tip of the ultrasonic “lance” was engaged into the lateral hole of the supra-structure. The ultrasonic vibrations were applied with a slight counter-clockwise rotational torque applied via the lance. The experiment was performed under water irrigation.

Results with Plaster

[0075] After a short while (depending from the type of the material into which the implants were embedded) the dental implant became loose. The time it took to loosen the dental implants seems not to be strongly dependent from:

[0076] The diameter of the embedded dental implant;

[0077] The length of the dental implant;

[0078] The height of the titanium supra-structure;

[0079] The type of material used for embedding the dental implant (with the exception of the epoxy bone cement).

[0080] A dental implant embedded in plaster can be loosened by applying ultrasonic vibrations combined with a light counter-clockwise torque to a titanium supra-structure being rigidly connected onto the dental implant platform. After the retrieval of the explanted dental implant following loosening and removal, no sign of macroscopic damages can be detected neither at the surface of the dental implant nor in the plaster. The dental implant platform is seemingly intact too. The stainless steel screw seems not to suffer from fatigue, and the tendency for loosening of the fixation-screw during loosening of the dental implant is negligible.

[0081] The loosening mechanism of the dental implant from plaster seems to occur at the implant to plaster interface: indeed, there is no sign indicating that the host material is destroyed in a zone beyond the interface as the windings are still clearly visibly in the former implant bed. Regarding heat generation, there are no visible signs indicating that heat generation cannot be controlled with simple irrigation: indeed, the plaster in the proximity of the former dental implant bed does not present sign of discoloration following the ultrasonic loosing of the dental implant.

Results with Bone-Like Material

[0082] A dental implant embedded in a plastic material (Polyurethane foam with bony-like mechanical properties) can be loosened and removed quite quickly when ultrasonic forces combined with a slight counter-clockwise torque are applied to a titanium supra-structure being rigidly fixed to the dental implant platform with an internal stainless steel screw. The dental implant surface as well as the dental implant platform is fully intact after removal. The bony-like plastic material seems not to be burned or overheated after loosening and removal of the dental implant when the loosening procedure is performed under irrigation with water. The stainless steel screw does not break, and the tendency for loosening of the supra-structure is minimal.

Results with Excised Fresh Bovine Tibia Bones

[0083] A dental implant being friction-locked into very dense cortical bovine tibia bone (torque way beyond 35 N/cm) can be loosened by ultrasonic vibrations within 30 seconds. The surface of the friction-locked dental implant being removed from bovine bone is seemingly completely unaffected after removal. This means that the dental implant, in a clinical situation, could probably be reinserted after its loosening (the properties of the titanium of the dental implants has to be tested in further trials to assure that such a dental implant will not be more prone to fatigue than normal). The bone-walls of the implant bed is slightly discolored after loosening of a friction-locked implant indicating that at least some local temperature increase is generated despite water-irrigation during the loosening procedure (which adds to the heat already generated during insertion/friction-locking). To which extend such heating will affect the vitality of the surrounding bone and neighboring structures can only be tested in animal experiments. The time needed to loosen the dental implant was seemingly independent from the height of the titanium supra-structure mounted on top of the dental implant platform. In addition, the time needed to loosen the dental implant was seemingly independent of the height of the dental implant being friction locked. The screw connecting the supra-structure with the platform of the dental implant is not damaged by the ultrasonic vibrations applied to the supra-structure. However, it may occur that the screw loosens and has to be re-tightened.

[0084] Results with hard epoxy-bone cementum. So far, a dental implant embedded into very hard epoxy-cement cannot be loosened by the use of ultrasonic vibrations (applied for a maximum of 5 minutes), no matter what height of the supra-structure mounted on the dental implant platform, and not matter how much counter-clockwise torque that is applied to the supra-structure. However, even though the dental implant is not loosened by the ultrasonic force, a very positive finding is that the stainless steel screw that rigidly connects the supra-structure with the dental implant platform does not break due to fatigue. And, the tendency for loosening of the supra-structure is still minimal if the stainless steel screw is tightened sufficiently beforehand. Though it was not possible to loosen a dental implant embedded in hard epoxy-cementum, still this finding is not reputed to be very relevant for the ability of ultrasonic vibration in removing dental implants from humans. The reason is that the mechanic properties of epoxy-cement is very far from vital jaw-bone, and thus this setup is very far from normal biological conditions in which the invention is intended to work.

[0085] As a conclusion on the experiments, ultrasonic vibrations combined with a slight counter-clockwise rotation is a suitable method to effectively loosen a dental implant being embedded in either plaster or a bony-like material. Neither the plaster nor the bony-like material is destroyed. The same working-principle is very time-effective to loosen a dental implant being friction-locked in fresh bovine cortical tibial bone. However, the bony bed is slightly discolored due to heat generation. Thus, the experiments indicate that the invention is suited for loosening and removing osseointegrated or friction-locked dental implants in humans time-effectively, non-invasively and non-traumatically.

To sum up, the invention provides a device for loosening a threaded dental implant I mounted and possibly osseointegrated in a jaw bone B of a patient. In the addition the invention can be used to facilitate easy insertion of dental implants.

An ultrasound vibration actuator USV, e.g. a piezoelectric transducer, generates ultrasonic vibrations USF with a frequency within the range 20-50 kHz, preferably within 24-36 kHz, and preferably in a direction around the longitudinal axis of the dental implant I. The actuator USV being a handpiece H in a structural connection with a handpiece head HD, is connected to a fitting-means F. The handpiece head HD with the fitting means F is arranged for entering the patient's mouth. The fitting-means F is shaped to provide a rigid engagement directly with the dental implant platform I**1**, I**2** or alternatively with an adaptor AD**1**, AD**2** rigidly attached to the dental implant platform I**1**, I**2**. The adaptor AD**1**, AD**2** is rigidly tightened to the platform of the dental implant I**1**, I**2** with a locking-screw penetrating through the central axis of the adaptor AD**1**, AD**2** into the inner wind of the dental implant I**1**, I**2**. Through the fitting-means F the handpiece head HD transmits ultrasonic vibrations USF to the dental implant I during either insertion or removal of a dental implant. This device is suitable for atraumatic removal of a dental implant as well as for bone saving insertion of a dental implant. Preferably, the handpiece H can also provide screwing and unscrewing directions to insert or remove a threaded dental implant I. A torque for such screwing or unscrewing can be applied manually by the operator of the handpiece H, or by a motor in or in connection with the handpiece H.

[0086] Although the present invention has been described in connection with the specified embodiments, it is not intended to be limited to the specific form set forth herein. Rather, the scope of the present invention is limited only by the accompanying claims. In the claims, the term "comprising" or "including" does not exclude the presence of other elements. Additionally, although individual features may be included in different claims, these may possibly be advantageously combined, and the inclusion in different claims does not imply that a combination of features is not feasible and/or advantageous. In addition, singular references do not exclude a plurality. Thus, references to "a", "an", "first", "second" etc. do not preclude a plurality. Furthermore, reference signs in the claims shall not be construed as limiting the scope.

1. A device for loosening a dental implant from the jawbone of a patient, the device comprising:
- an ultrasonic vibration actuator arranged to generate an ultrasonic vibration force with a frequency within the range 20 kHz to 50 kHz,
- a handpiece with a head which is arranged for entering the patient's mouth,
- a socket positioned in a head end of the handpiece and shaped to provide a substantially rigid engagement with the dental implant or an intermediate rigidly fixed adaptor wherein the socket is connected to the ultrasonic vibration actuator so as to transmit the ultrasonic vibration force to the dental implant for loosening the dental implant from the jaw bone.

2-18. (canceled)

19. The device according to claim **1**, wherein the ultrasonic vibration actuator and the socket are arranged to apply an ultrasonic vibration force with a rotational component to the dental implant, so as to cause the dental implant to perform an ultrasonic vibration around its longitudinal axis.

20. The device according to claim **1**, comprising a motor connected to the socket so as to turn the socket counter-clockwise for unscrewing the dental implant, upon activation.

21. The device according to claim **20**, wherein the motor is arranged to turn the socket both clockwise and counter-clockwise upon selective activation, so as to enable selective screwing or unscrewing of the dental implant.

22. The device according to claim **20**, wherein the ultrasonic vibration actuator and the motor are arranged to function simultaneously, so as to enable application of the ultrasonic force to the dental implant during screwing or unscrewing.

23. The device according to claim **1**, wherein the ultrasonic vibration actuator is driven by a piezoelectric transducer element or pneumatics.

24. The device according to claim **1**, wherein a function of the ultrasound vibration actuator is controlled by a processor arranged to at least start and stop the generation of ultrasonic vibration force upon user activation.

25. The device according to claim **24**, wherein the processor is arranged within a separate control box connected to the handpiece by a cord, wherein the control box has a user interface with a user activated controller.

26. The device according to claim **24**, wherein the processor is arranged to control the ultrasonic vibration actuator so as to vary at least one parameter of the ultrasonic vibration force.

27. The device according to claim **26**, wherein the processor is arranged to vary the ultrasonic vibration force with respect to amplitude, frequency content, or direction.

28. The device according to claim **27**, wherein the processor is arranged to vary the amplitude of the ultrasonic vibration force.

29. The device according to claim **28**, wherein the processor is arranged to temporally vary the amplitude of the ultrasonic vibration force.

30. The device according to any of claim **26**, wherein the processor is arranged to vary a frequency content of the ultrasonic vibration force.

31. The device according to claim **1**, comprising a plurality of removable adaptors which each have a first end shaped to provide a tight fit to the socket and with a second end shaped to provide a rigid fit to respective different platform shapes of associated types of dental implants.

32. The device according to claim **1**, arranged to apply a torque to the socket upon activation so as to turn the socket counter-clockwise for unscrewing the dental implant.

33. The device according to claim **32**, wherein the torque is applied by a motor.

34. The device according to claim **32**, wherein the torque is applied manually by an operator of the device.

35. A method for loosening a dental implant from the alveolus jaw-bone of a patient, the method comprising:
- generating an ultrasonic vibration force with a frequency within the range 20 kHz to 50 kHz, and
- transmitting the ultrasonic vibration force directly to the dental implant or by the use of an intermediate rigidly fixed adaptor for loosening the dental implant from the alveolus jaw-bone.

* * * * *
