# Supplementary material for: Reversal of Osseointegration as a Novel Perspective for the Removal of Failed Dental Implants: A Review of Five Patented Methods
Source: Materials (Basel). 2021 Dec 17;14(24):7829. doi: 10.3390/ma14247829 (PMC8707383; doi:10.3390/ma14247829)
Supplement: Supplementary file 1 [file materials-14-07829-s001.zip › P5 (US) Winnen, Germany, Winnen.pdf]

US010136935B2

(12) **United States Patent**
Winnen

(10) **Patent No.: US 10,136,935 B2**

(45) **Date of Patent: Nov. 27, 2018**

(54) **DEVICE FOR THE CONTROLLED REMOVAL OF OSSEOINTEGRATED IMPLANTS AND IMPROVED OSSEODISINTEGRATABLE IMPLANTS**

(71) Applicant: **Rolf G. Winnen**, Duesseldorf (DE)

(72) Inventor: **Rolf G. Winnen**, Duesseldorf (DE)

(*) Notice: Subject to any disclaimer, the term of this patent is extended or adjusted under 35 U.S.C. 154(b) by 0 days.

(21) Appl. No.: **14/911,312**

(22) PCT Filed: **Aug. 22, 2014**

(86) PCT No.: **PCT/EP2014/067928**

§ 371 (c)(1),
(2) Date: **Feb. 10, 2016**

(87) PCT Pub. No.: **WO2015/025042**

PCT Pub. Date: **Feb. 26, 2015**

(65) **Prior Publication Data**

US 2016/0192975 A1 Jul. 7, 2016

(30) **Foreign Application Priority Data**

Aug. 23, 2013 (EP) 13181624

(51) **Int. Cl.**
A61B 17/92 (2006.01)
A61C 8/00 (2006.01)
(Continued)

(52) **U.S. Cl.**
CPC ***A61B 17/92*** (2013.01); ***A61B 34/10*** (2016.02); ***A61C 8/005*** (2013.01); ***A61C 8/0006*** (2013.01);
(Continued)

(58) **Field of Classification Search**
None
See application file for complete search history.

(56) **References Cited**

U.S. PATENT DOCUMENTS

4,170,990 A * 10/1979 Baumgart A61B 17/0644 606/62
5,188,109 A 2/1993 Saito
(Continued)

FOREIGN PATENT DOCUMENTS

AT 385 196 B 2/1988
EP 2578183 A1 4/2013
(Continued)

OTHER PUBLICATIONS

Office Action dated Jan. 17, 2017, which issued in corresponding European Patent Application No. 14 753 270.9, 5 pages.
(Continued)

Primary Examiner — Nicholas Lucchesi
Assistant Examiner — Shannel Wright
(74) *Attorney, Agent, or Firm* — Rothwell, Figg, Ernst & Manbeck, P.C.

(57) **ABSTRACT**

The present invention relates to a medical device for freeing an implant from the bone of a patient to which the implant adheres to and to an implant for insertion into a bone of a patient, designed such that the implant is more homogenously heatable or coolable in a controlled way. Also provided is a method of removing an implant from a bone of a patient and a computer readable storage medium having recorded thereon a database structure.

14 Claims, 4 Drawing Sheets

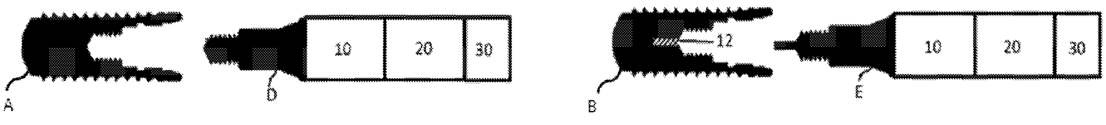

(51) **Int. Cl.**

A61B 34/10	(2016.01)
A61C 8/02	(2006.01)
A61N 1/40	(2006.01)
A61B 34/00	(2016.01)
A61B 17/00	(2006.01)
A61F 7/00	(2006.01)

(52) **U.S. Cl.**

CPC ***A61C 8/0012*** (2013.01); ***A61C 8/0089*** (2013.01); ***A61N 1/403*** (2013.01); *A61B 2017/00017* (2013.01); *A61B 2034/256* (2016.02); *A61F 2007/0013* (2013.01); *A61F 2007/0057* (2013.01); *A61F 2007/0088* (2013.01); *A61F 2007/0095* (2013.01)

(56) **References Cited**

U.S. PATENT DOCUMENTS

5,330,481 A * 7/1994 Hood A61B 17/8847 128/898
5,980,528 A * 11/1999 Salys A61B 17/92 606/100
2009/0024161 A1* 1/2009 Bonutti A61B 17/0401 606/213
2010/0063499 A1* 3/2010 Pein A61F 2/4603 606/41
2010/0100139 A1* 4/2010 Young .... A61F 2/4603 606/86 A
2011/0282418 A1* 11/2011 Saunders A61B 17/7002 607/105
2013/0071813 A1* 3/2013 Braegger A61C 8/0089 433/173
2014/0248581 A1* 9/2014 Petersen A61C 8/0089 433/119

FOREIGN PATENT DOCUMENTS

KR 20100082188 A * 7/2010
WO 9748348 A1 12/1997
WO 2012093044 A1 7/2012
WO 2012142517 A2 10/2012
WO WO 2013050046 A1 * 4/2013 .... A61C 8/0089

OTHER PUBLICATIONS

International Preliminary Report on Patentability in PCT/EP2014/067928 dated Feb. 23, 2016 (9 pages).
European Office Action issued in application No. 14 753 270.9 dated May 26, 2017, 6 pages.
International Search Report cited in PCT/EP2014/067928, dated Oct. 21, 2014, 5 pages.
Communication pursuant to Article 94(3) EPC dated Nov. 23, 2017, cited in European Application No. 14 753 270.9, 6 pages.

* cited by examiner

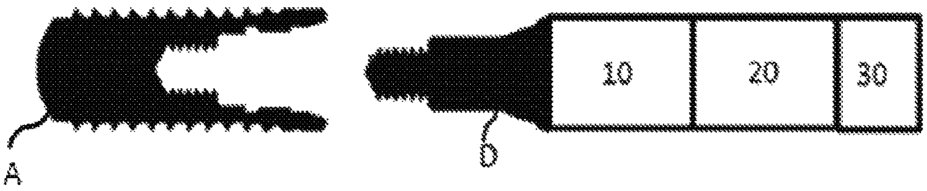

FIG. 1A

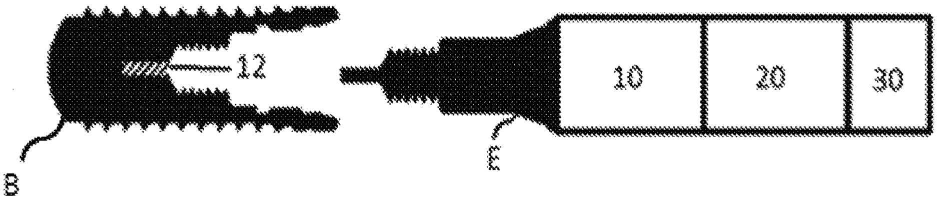

FIG. 1B

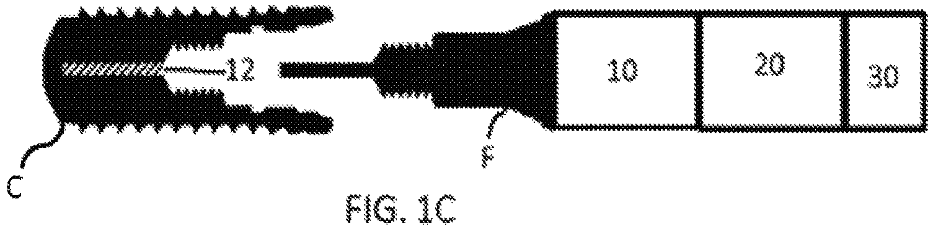

FIG. 1C

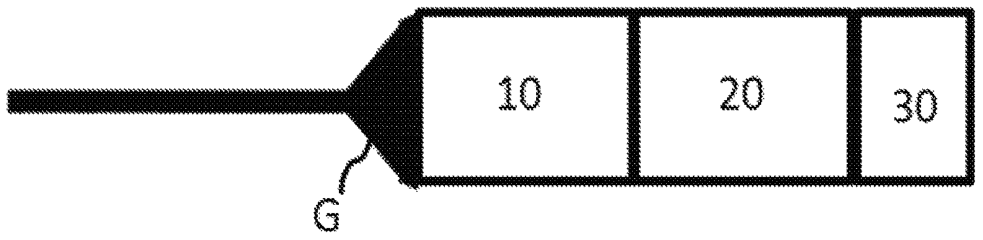

FIG. 1D

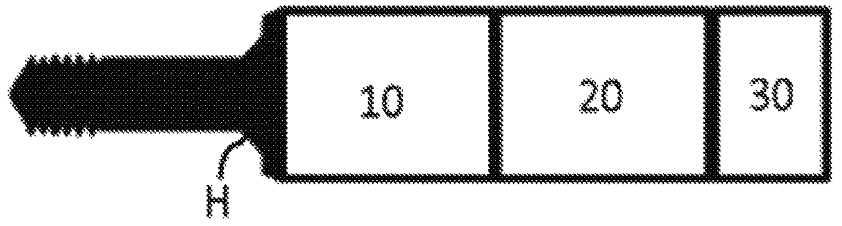

FIG. 1E

Figure 2

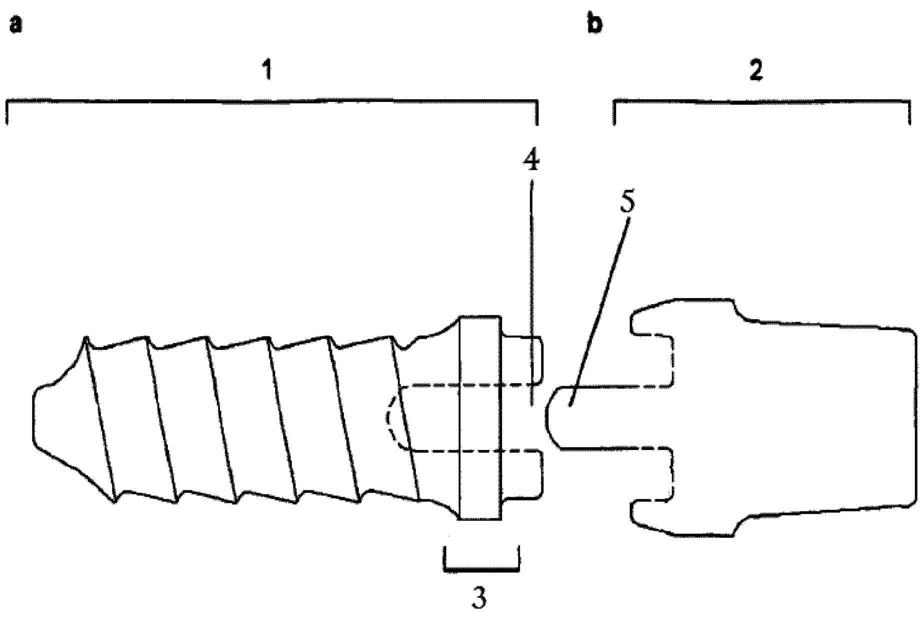

Figure 3

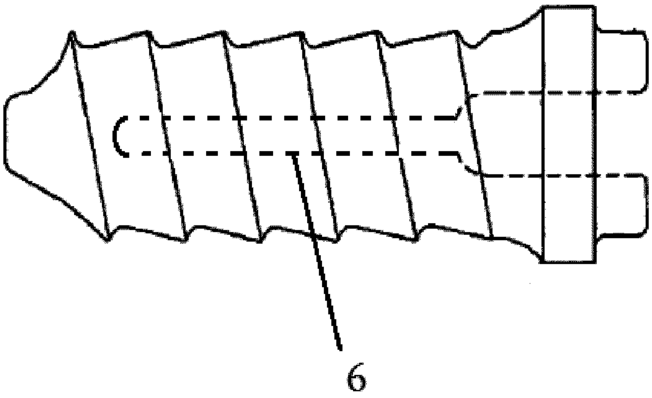

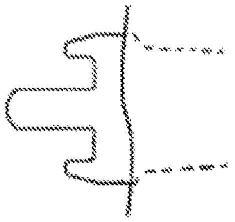

FIG. 4A

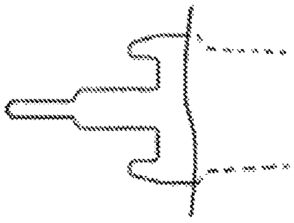

FIG. 4B

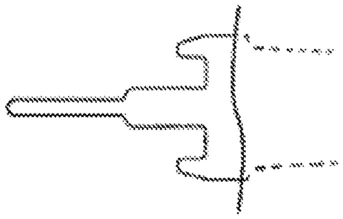

FIG. 4C

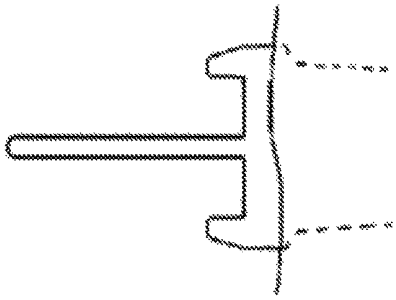

FIG. 4D

DEVICE FOR THE CONTROLLED REMOVAL OF OSSEOINTEGRATED IMPLANTS AND IMPROVED OSSEODISINTEGRATABLE IMPLANTS

CROSS REFERENCE TO RELATED APPLICATION

This application is a 35 U.S.C. 371 National Phase Entry Application from PCT/EP2014/067928, filed Aug. 22, 2014, which claims the benefit of European Patent Application No. 13181624.1 filed on Aug. 23, 2013, the disclosure of which is incorporated by reference in its entirety.

The present invention relates to a medical device for freeing an implant from the bone of a patient to which the implant adheres to and to an implant for insertion into a bone of a patient, designed such that the implant is more homogenously heatable or coolable in a controlled way. Also provided is a method of removing an implant from a bone of a patient and a computer readable storage medium having recorded thereon a database structure.

BACKGROUND OF THE INVENTION

The present invention relates to a device for the controlled "osseodisintegration" of partially or completely osseointegrated implants by forming a denatured tissue layer in the implant bedding where the layer is preferably very thin. The denatured layer then allows the atraumatic removal of the implant which conserves as much of the bone material surrounding the implant as possible.

It is known that medical implants can be used for therapeutic purposes. Some types of implants are designed to adhere to the bone of the subject. If such a connection is formed it is also termed osseointegration. Osseointegration generally refers to the structural and functional connection between living bone and the surface of a load-bearing implant. Preferably said connection is direct such that no scar tissue, cartilage or ligament fibers are present between the bone and the implant surface. An osseointegrated implant is mostly a type of implant which comprises pores into which osteoblasts and supporting connective tissue can migrate to form said connection. When osseointegration occurs, the implant is tightly held in place by the bone. The process typically takes several weeks or months to occur.

In dentistry, implants capable of osseointegration are used as so-called dental implants onto which (aesthetic) prostheses are attached, to replace one or more missing teeth. A dental implant showing typical implant elements is disclosed in e.g. EP 2 412 335 A1. In other medical fields implants capable of osseointegration are used mostly when the implant is load-bearing such as when implanting total joint replacement prostheses. If the implant is not intended for osseointegration, the implant will typically be cemented into the subject by using a suitable cement composition. Examples are cemented and/or cementless endo-prostheses as total joint replacement for hip, knee, shoulder, elbow, and spine.

Implants in medicine are shaped very differently depending on the use. These implants are frequently made of inorganic material such as a specific metal, a metal alloy or a ceramic. In dentistry, the dental implants which are most frequently used have a mostly helical shape which can be screwed into bone and typically are made of titanium, titanium alloys or sometimes ceramics. Exemplary schematics of dental implants (A) are shown in FIG. **1**A and FIG. **2** of this application.

The surfaces of the implants can be conditioned (e.g. roughened) by various methods to achieve a good growth of the bone tissue onto the enlarged contact surface. It is critical that the implant is successfully osseointegrated. During the time of healing it can occur that the implant is only partially osseointegrated. Also implants which have been implanted in the patient for some time may lose part or all of the osseointegration. This process is also referred to as osseodisintegration. In these cases it seems sensible to speak of "unwanted" osseodisintegration whereas this invention refers to intentionally induced, "intended" osseodisintegration. If an implant completely loses osseointegration this usually results in a loosening of the implant relative to the bone so that the implant can be removed generally without much trauma to or destruction of the bone material. For example, screw implants can be removed by unscrewing the implant. Sometimes, especially in orthopedics, a new implant can be set in the same surgical procedure or mostly after the old implant was removed and the bone tissue had sufficient time to heal.

Often, however, it becomes necessary to remove an implant which is still partially or fully osseointegrated. This situation can arise for example if an implant failed to be completely osseointegrated after implantation or when an implant is in an incorrect position or when an irreparable fracture occurred in the implant or when there is an active inflammation in the implant bedding (periimplantitis).

In such cases, the current treatment procedure is that the implant is removed by drilling out, cutting out or milling off sufficient bone surrounding the implant such that the implant is freed from the bone material and can be removed. The harm done to the bone material in such a procedure is quite severe resulting in a large loss of bone substance. The gap made between the bone and the implant is typically at minimum 2 mm to make it possible that sufficient cooling agent can be flushed into the gap. Furthermore, such gap is also a result of drilling, cutting and milling equipment which must be structurally sufficiently stable and hence sufficiently thick to remove the bone material.

If a dental implant having mostly a diameter of only about 3-6 mm is removed in this way, this treatment may result in a bone cavity which is almost twice the diameter of the cavity in which the implant was originally located. As a consequence of the large loss of bone substance, it becomes frequently difficult to insert a new implant. If sensitive anatomical structures are close by, which is very often the case, the removal of the implant by this traditional method bears a very high risk of permanently damaging these structures. To avoid these risks sometimes the compromised implant will be left in place, sometimes even without using it, as so called "sleeping implant".

An alternative treatment has been described to "unscrew" a partially or fully osseointegrated implant by using strong forces, breaking the bone structure. Of course such large forces required to remove the implant result in a very uncomfortable situation for the patient during the removal of the implant and also include a high risk of permanently damaging sensitive anatomical structures.

Thus, there is still a high and unmet need for a safe and effective means for removing partially or completely osseointegrated implants from bone without destroying a large part of the bone material in the process. Also implants are needed which can be easily removed from bone material once there is the indication for removing the implant.

SUMMARY OF THE INVENTION

Thus, it was one object of the invention to overcome the above outlined problems. In particular it was an object of the

invention to provide a safer medical device for freeing an implant from the bone of a patient to which the implant adheres to. Since this release from the bone occurs in a controlled manner, by influencing the temperature of the implant in a controlled process, the implant can be removed while preserving most of the bone substance. In this method only bone tissue which is in direct contact with the implant is selectively denatured by a controlled heat and/or cold pulse. This process is facilitated by a further aspect of the invention which provides novel implants which enable the surgeon to remove these implants from bone more easily even when the implants are at least partially osseointegrated in the bone. It is expected that such an implant according to the invention when osseointegrated can be heat and/or cold treated in a controlled manner such that part of the bone tissue which contacts the implant is selectively destroyed just in an amount sufficient to allow removal of the implant affecting a much thinner layer of bone material than using the conventional method.

Thus, the present invention provides in a first aspect a medical device for releasing an implant from the bone of a patient to which the implant adheres to, the medical device comprising

(A) a device capable of heating and/or cooling the implant, wherein the heating energy and/or cooling agent produced or released by the device (A) is preferably transferred from the device (A) to said implant via the coupling device (C);
(B) a controlling device controlling the device (A);
(C) a coupling device capable of releasably connecting to said implant while said implant adheres to the bone of the patient; and
(D) optionally an input device operably linked with the controlling device (B).

Also provided is an implant for insertion into a bone of a patient, designed such that the implant is controllably heatable or coolable. One advantage of the implant of the invention is that it is more homogeneously heatable and/or coolable than a conventional implant.

The invention further provides a method of removing an implant from a bone of a patient comprising the step of heating and/or cooling the implant, wherein at least part of the implant is osseointegrated in the bone.

A further aspect of the invention relates to a computer readable storage medium having recorded thereon a database structure where the database structure is capable of storing data representing

(a) implant information such as information that defines the type, dimensions, mass, surface structure and/or material of the implant or that defines the cavity within the implant; and/or
(b) information defining the properties of the coupling device such as its shape, the amount of connecting surfaces with the implant and/or its thermal conductivity; and/or
(c) information defining the surface of the implant which is osseointegrated ; and/or
(d) information defining the type of heating energy and/or cooling agent to be used and optionally the amount of heating energy and/or cooling agent necessary to reach osseodisintegration of an implant as defined in (a) preferably with minimal trauma to the surrounding tissues; and/or
(e) information for controlling the device (A) to heat or cool the implant such to achieve osseodisintegration of bone material contacting the implant or located within a distance of less than a predefined distance from the surface of said implant; and/or
(f) information defining the temperature, temperature curve, heating time, cooling time, frequency of heating and/or cooling which are suitable to effect osseodisintegration of an implant as defined in (a); and/or
(g) information defining the bone density of the bone contacting or surrounding the implant; and/or
(h) information defining the date on which the implant was implanted into the patient; and/or
(i) information defining the age of the patient.

A preferred embodiment of said aspect of the invention relates to a computer readable storage medium having recorded thereon a database structure where the database structure is capable of storing data representing

(a) implant information such as information that defines the type, dimensions, mass and/or material of the implant or that defines the cavity within the implant;
(b) information defining the properties of the coupling device such as its shape, the amount of connecting surfaces with the implant and/or its thermal conductivity
(c) information defining the surface of the implant which is osseointegrated
(d) information defining the type of heating energy and/or cooling agent to be used and optionally the amount of heating energy and/or cooling agent necessary to reach osseodisintegration of an implant as defined in (a) preferably with minimal trauma to the surrounding tissues; and/or
(e) information for controlling the device (A) to heat or cool the implant such to achieve osseodisintegration of bone material contacting the implant or located within a distance of less than a predefined distance from the surface of said implant.

DETAILED DESCRIPTION OF THE INVENTION

Before the present invention is described in detail below, it is to be understood that this invention is not limited to the particular methodology, protocols and reagents described herein as these may vary. It is also to be understood that the terminology used herein is for the purpose of describing particular embodiments only, and is not intended to limit the scope of the present invention which will be limited only by the appended claims. Unless defined otherwise, all technical and scientific terms used herein have the same meanings as commonly understood by one of ordinary skill in the art. Preferably, the terms used herein are defined as described in "A multilingual glossary of biotechnological terms: (IUPAC Recommendations)", Leuenberger, H. G. W, Nagel, B. and K6101, H. eds. (1995), Helvetica Chimica Acta, CH-4010 Basel, Switzerland).

Throughout this specification and the claims which follow, unless the context requires otherwise, the word "comprise", and variations such as "comprises" and "comprising", will be understood to imply the inclusion of a stated integer or step or group of integers or steps but not the exclusion of any other integer or step or group of integers or steps. In the following passages different aspects of the invention are defined in more detail. Each aspect so defined may be combined with any other aspect or aspects unless clearly indicated to the contrary. Also any feature indicated as being preferred or advantageous may be combined with any other feature or features indicated as being preferred or advantageous.

Some documents are cited throughout the text of this specification. Each of the documents cited herein (including all patents, patent applications, scientific publications, manufacturer's specifications, instructions, DIN norms etc.), whether supra or infra, are hereby incorporated by reference in their entirety. Nothing herein is to be construed as an admission that the invention is not entitled to antedate such disclosure by virtue of prior invention.

In the following some general definitions are provided. These terms will in each instance of its use in this specification have the respectively defined meaning and preferred meanings.

The expression "proximal" as used when referring to an implant of the present invention shall refer to that end of the implant which is closer to the bone into which the implant is inserted. The opposite expression "distal" refers to that end of the implant which is farther away from the bone into which the implant is inserted. Thus, the proximal end of the implant is buried in the bone, whereas the distal end of the implant is to be exposed at the surface of the bone and/or may extend out of the bone (see for example the distal part (**3**) of the dental implant shown in FIG. **2**), after the implant has been implanted into the bone. If the implant is a dental implant and if the dental implant is part of a two-part medical device comprising an abutment (see for example FIG. **1**A (reference letter (D)) and FIG. **2** (reference number (**2**)) then the proximal end of the abutment can be coupled with the dental implant, whereas the distal end of the abutment can face the oral cavity and can receive for example a crown or a superstructure which may be placed upon the distal end of the abutment.

In the field of dentistry, positions on a tooth can be described inter alia by the terms "apical" and "coronal". The term "apical" refers to the direction towards the root tip(s) of a tooth. In contrast, the term "coronal" refers to the direction towards the crown of a tooth. These terms can also be applied to implants. Thus, herein the "apical" end of an implant is that end which is to be inserted into the bone. The "coronal" end of an implant is that end which is exposed to the outside or may extend out of the bone, after the implant has been implanted. Accordingly, in the present application the term "apical" is used interchangeably with the term "proximal" explained above. Likewise, the term "coronal" is used interchangeably with the term "distal" explained above.

The term "biocompatible ceramic" refers to ceramic materials, which are inert or substantially inert against the environment when implanted and are non-toxic. Preferably the term refers to ceramics comprising ceramic materials selected from the group consisting of zirconium dioxide, aluminum oxide, yttrium oxide, hafnium oxide, silicon oxide, magnesium oxide, cerium oxide, other metal oxides, and mixtures thereof. The pure or mixed ceramic materials can have various crystal forms including, for example, monoclinic, tetragonal, trigonal, hexagonal etc. as can be found e.g. in ruby and sapphire. Preferably, the ceramic material consists of zirconium dioxide, hafnium oxide, yttrium oxide, aluminum oxide and small amounts of other metal oxides. In preferred embodiments, the zirconium dioxide ceramic is selected from the group consisting of zirconium dioxide ceramic according to ISO 13356, alumina toughened zirconia (ATZ), zirconia toughened alumina (ZTA), so called "yttrium-stabilized zirconium" (see e.g. EP 0 218 853 A1 and EP 0 624 360 A1) and Y-TZPA (yttrium oxide stabilized tetragonal zirconia polycristals doped with alumina).

The term "biocompatible synthetic material" refers to an organic polymer preferably having an Emodule >2,000 N/mm', preferably >2,500 N/mm', preferably >3,000 N/mm', more preferably >3,500 N/mm'. A "biocompatible synthetic material" is preferably inert or substantially inert against the environment when implanted and is non-toxic. Preferred synthetic materials are selected from a group consisting of polycarbonate (PC), polyamide (PA), polyimide (PI), polyetheretherketone (PEEK), polyphenylenesulfide (PPSE), epoxide resin (EP), unsaturated polyester (UP), phenol resin (PF), melamine resin (MP), cyanate ester (CA), polytetrafluoroethylene (PTFE) and mixtures thereof. A particular preferred material is PEEK (e.g. PEEK-Optima®, which can be purchased from Invibio, Lancashire, U.K.). To improve the mechanical properties of biocompatible synthetic material, it can be reinforced by introducing other materials, especially fibres from glass, coal, aramide, or metal fibres. The resulting compounds are especially suitable synthetic materials due to their hardness. Examples for especially suitable fibres are aramide fibres (AF), coal fibres (CF) and glass fibres (GF). These can be used individually or as mixtures. Preferably, these fibres are embedded in one or more of the following duroplast or thermoplastic matrices: epoxide, polyester, phenol, cyanate ester, polyamide, polyimide, polyetheretherketone, polyphenylenesulfide. A number of such compounds are known in the art and comprise especially the group of glass fibre reinforced synthetic materials (GFK), carbon fibre reinforced synthetic material (CFK) and aramide fibre reinforced material (AF) as well as mixtures thereof. All of the above are also comprised in the term "biocompatible synthetic material".

As used herein "releasably connected" means that a user can disconnect the connection without physically or chemically damaging the parts making the connection. For example a releasable connection does not include a connection wherein the parts are soldered or glued together. Examples for releasable connections include, but are not limited to a screw connection, a clip or clamp connection and a plug connection.

The present invention will now be further described.

In a first aspect the invention provides a medical device for freeing an implant from the bone of a patient to which the implant adheres to, the medical device comprising

(A) a device capable of heating and/or cooling the implant, wherein preferably the heating energy and/or cooling agent produced or released by the device (A) is transferred from the device (A) to said implant via the coupling device (C);

(B) a controlling device controlling the device (A);

(C) a coupling device capable of releasably connecting to said implant while said implant adheres to the bone of the patient; and

(D) optionally an input device operably linked with the controlling device (B).

The implant can be any implant. Thus, the medical device of the invention is capable of operating with conventional implants as well as with optimized implants according to the invention (see detailed embodiments below). Preferred implants that can be used with the invention include an implant selected from the group consisting of an orthopaedic implant and a dental implant. Preferred orthopaedic implants include without limitation an implant selected from the group consisting of a hip implant, knee implant, shoulder implant, elbow implant and a spine implant. Most preferably, the implant that is used with the device according to the invention or as an implant of the invention (outlined further

below) is a dental implant which is at least partially osseointegrated in the mandibular or maxillary bone of a patient.

An implant according to the invention can be made from various materials including a biocompatible ceramic, a biocompatible synthetic material and a biocompatible metal or metal alloy. If the implant is made from a metal or metal alloy then it is preferred that the implant is made from titanium or a metal alloy such as tantalum titanium, Titanium 6AL-4V, a cobalt-chromium alloy, an alloy comprising titanium and zirconium (such as e.g. Roxolid by Straumann) or stainless steel.

The medical device according to the invention comprises a coupling device (C). The coupling device is preferably for connecting the device (A) to said implant. If an osseointegrated implant is to be removed which extends out of the bone then the implant and/or said device (C) can be shaped to permit a temporal attachment of the coupling device with the part of implant that extends out of the bone and is therefore accessible. If the implant is a dental implant then the medical device according to the invention preferably comprises a coupling device (C) which has at least part of the shape of a dental implant abutment which allows said coupling device (C) to be releasably connectable to said dental implant. Alternatively, or additionally the coupling device (C) may have the shape of a ring which allows said coupling device (C) to be releasably connectable to said implant for example by putting the coupling device (C) over at least part of the implant which extends out of the bone. If the implant does not extend out of the bone but part of the implant is accessible through the surface of the bone and/or the soft tissue then the coupling device (C) may comprise a protrusion which can be inserted into a respective cavity which is present in the osseointegrated implant for this purpose. The coupling device (C) is preferably designed to conduct the heating energy and/or cooling agent produced or released by the device (A). Alternatively, the coupling device (C) can also be designed only to enable the medical device of the invention to be attached directly to the implant to be heated and/or cooled. If the coupling device (C) is designed to conduct the heating energy and/or cooling agent to the implant then it is preferred that the coupling device contacts at least 10%, 20%, 30%, 40%, 50%, 60%, 70%, 80% or at least 90% of that surface area of the osseointegrated implant which is exposed to the exterior of the bone. Different areas of contact of the different coupling devices to the implant can be used according to the amount and expected region of osseointegration.

The coupling device (C) is not necessary solid but may comprise channels through which the heating energy and/or cooling agent can pass to reach the implant. Such an embodiment may be preferable for example when using a laser to heat the implant or cold air or cold liquid to cool the implant.

Also if the part of the coupling device (C) that conducts the heating energy is solid, then the coupling device is preferably made from a material with good thermal conductivity. In one preferred embodiment of the medical device according to the invention the device comprises a coupling device (C) made from a material that has a thermal conductivity of at least 0,5, preferably at least 22 and most preferably at least 400 W/(m·K). The coupling device (C) can for example be made from gold, silver or copper or an alloy comprising one or more of the aforementioned metals or of the same material as the implant.

If the thermal energy is an electromagnetic radiation, the coupling device (C) or part thereof is preferably permeable for said electromagnetic radiation. In a particularly preferred embodiment the coupling device (C) or part thereof is translucent.

To conduct the thermal energy more efficiently, the coupling device (C) can in one preferred embodiment also consist of or comprise a thermally conductive paste. The paste is preferably non-toxic and may be for example a silicone or non-silicone based grease preferably comprising particles having a thermal conductivity of at least 100 W/(m·K). Thermal conductivity can be measured for example according to ASTM Standard E1225-04.

In one preferred embodiment the thermally conductive paste is inserted into a cavity of the implant which is accessible and not covered by the bone tissue and then the device (A) of the device of the invention is brought into contact with the thermally conductive paste and if required also with the implant.

The coupling device (C) is preferably releasably connecting the device (A) and/or (B) to said implant. In one preferred embodiment, the coupling device (C) is shaped such that a part of the implant can be selectively heated and/or cooled. "Selectively heated and/or cooled" means that the shape of said coupling device (C) conducts said heating energy and/or cooling agent only to one particular part of the implant. As a consequence, this part of the implant will be heated and/or cooled most. Exemplary embodiments of such a coupling device (C) are shown in FIGS. **1**D, **1**E (reference letters (G)-(H)) and in FIG. **4**D (reference letter (d)). If no selective heating and/or cooling is required preferred coupling devices (C) of the invention as shown in FIGS. **1**A-E (reference letters (D)-(H)) and in FIGS. **4**A-D (reference letters (a)-(d)) can be used. If the implant is a dental implant it is preferably not self-cutting, i.e. it preferably comprises a thread but no cutting section. The reason for this being, that after the intentional osseodisintegration of the implant according to this invention the implant shall be screwed out of the bone with low forces. The substantial groove in the implant, which is typically associated with self-cutting implants, might hinder the screwing out process.

Referring to FIG. **1**, the medical device according to the invention comprises a controlling device **20** controlling the heating/cooling device **10** by which the amount of heating energy and/or cooling agent transferred from the heating/cooling device **10** to said implant (B) and (C) is controlled. Preferably, the controlling device **20** controls the amount of heating energy and/or cooling agent delivered to the implant (B) and (C) to an amount that is sufficient to achieve osseodisintegration of bone material which contacts the implant; thereby the osseodisintegration is preferably limited to the bone material located within a distance of about 0.1 to 3 mm from the surface of the implant. Said amount is controlled by the controlling device **20** either by limiting the time during which the heating energy and/or cooling agent is produced or released and/or by limiting the actual amount of heating energy and/or cooling agent produced or released, respectively. For example in one embodiment, the controlling device may comprise a timer in order to limit the duration during which said device (A) produces or releases said heating energy and/or cooling agent. Preferably, the controlling device is capable of computing and limiting the total amount of heating energy and/or cooling agent produced or released for a pre-set treatment time. Preferably the controlling device reduces the heating energy and/or cooling agent for long treatment times and increases the heating energy and/or cooling agent for short treatment times. Some exemplary most preferred settings are selected from the

group consisting of 10 minutes heating of the implant to 50° C.-60° C. and under 10 seconds heating of the implant to 100° C.-300° C. Further examples are provided also below.

In one preferred embodiment, the controlling device (B) comprises a database and functions by using information in said database, wherein the database comprises

(a) implant information such as information that defines the type, dimensions, mass and/or material of the implant or information that defines the cavity within the implant (such as length and diameter of a screw channel, or other geometries for taking up the abutment);
(b) information defining the properties of the coupling device such as its form, its surface connecting with the implant, its thermal conductivity
(c) information defining the surface of the implant which is osseointegrated
(d) information defining the type of heating energy and/or cooling agent to be used and optionally the amount of heating energy and/or cooling agent necessary to reach osseodisintegration of an implant as defined in (a) preferably with minimal trauma to the surrounding tissues; and/or
(e) information for controlling the device (A) to heat or cool the implant such to achieve osseodisintegration of bone material contacting the implant or located within a distance of less than a predefined distance from the surface of said implant.

In one preferred embodiment, the controlling device (B) comprises a database and functions by using information in said database, wherein the database comprises

(a) implant information such as information that defines the type, dimensions, mass, surface structure and/or material of the implant or information that defines the cavity within the implant (such as length and diameter of a screw channel, or other geometries for taking up the abutment); and/or
(b) information defining the properties of the coupling device such as its form, its surface connecting with the implant, its thermal conductivity; and/or
(c) information defining the surface of the implant which is osseointegrated; and/or
(d) information defining the type of heating energy and/or cooling agent to be used and optionally the amount of heating energy and/or cooling agent necessary to reach osseodisintegration of an implant as defined in (a) preferably with minimal trauma to the surrounding tissues; and/or
(e) information for controlling the device (A) to heat or cool the implant such to achieve osseodisintegration of bone material contacting the implant or located within a distance of less than a predefined distance from the surface of said implant; and/or
(f) information defining the temperature, temperature curve, heating time, cooling time, frequency of heating and/or cooling which are suitable to effect osseodisintegration of an implant as defined in (a); and/or
(g) information defining the bone density of the bone contacting or surrounding the implant; and/or
(h) information defining the date on which the implant was implanted into the patient; and/or
(i) information defining the age of the patient.

The database can be hard-coded into the controlling device (B) or can be stored in the controlling device (B) or elsewhere in the medical device on a memory device which is either read-only or can be readable and writable. Preferred memory devices are USB-Sticks, SD-Cards, CD-Rom, EPROM, flash-EEPROM, floppy disc or the like. In any case according to the invention said memory device comprises the above outlined information which is stored on the memory device. In one embodiment of the medical device of the invention the medical device (for example the controlling device (B)) further comprises a temperature sensor which is capable of measuring the temperature of the implant. Preferably, the sensor can be attached to the implant or to the coupling device (C) to measure the temperature of the implant. In this preferred embodiment, the controlling device (C) is capable of monitoring the temperature of the implant and preferably capable of stopping device (A) if a temperature is reached which is sufficient for inducing osseodisintegration.

In the illustrated embodiment of the medical device of the invention shown in FIG. **1**, the device also comprises an input device **30**. Preferably said input device **30** is capable of receiving a parameter from an operator operating the medical device; wherein the parameter

(i) defines the type, dimensions, mass and/or material of said implant;
(ii) defines the expected amount of osseointegrated surface on the implant
(iii) defines the amount and/or duration of the heating and/or cooling;
(iv) defines the expected position or region of the osseointegrated surface on the implant;
(v) defines the implant temperature which is reached during heating and/or cooling of the implant and/or
(vi) modifies the amount and/or duration of the heating and/or cooling suggested by the database in the controlling device;
(vii) modifies the implant temperature suggested by the database in the controlling device which shall be reached during heating and/or cooling of the implant and/or
(viii) is a parameter selected from any of the parameters defined herein above or below in the context of said database.

In a further preferred embodiment said input device **30** is capable of receiving a parameter from an operator operating the medical device; wherein the parameter

(i) defines the type, dimensions, mass, surface structure and/or material of said implant; and/or
(ii) defines the expected amount of osseointegrated surface on the implant; and/or
(iii) defines the amount and/or duration of the heating and/or cooling; and/or
(iv) defines the expected position or region of the osseointegrated surface on the implant; and/or
(v) defines the implant temperature which is reached during heating and/or cooling of the implant and/or
(vi) modifies the amount and/or duration of the heating and/or cooling suggested by the database in the controlling device; and/or
(vii) modifies the implant temperature suggested by the database in the controlling device which shall be reached during heating and/or cooling of the implant and/or
(viii) is a parameter selected from any of the parameters defined herein above or below in the context of said database; and/or
(ix) is a parameter defining the temperature, temperature curve, heating time, cooling time, frequency of heating and/or cooling which are suitable to effect osseodisintegration of an implant as defined in (i); and/or
(x) is a parameter defining the bone density of the bone contacting or surrounding the implant; and/or

(xi) is a parameter defining the date on which the implant was implanted into the patient; and/or

(xii) is a parameter defining the age of the patient.

If the implant is a dental implant then the parameter may also define the dimension of the cavity of the implant, for example the diameter and depth of the screw channel or other geometries of the implant's cavity for taking up the abutment. Referring to FIG. **1**, the parameter received by the input device **30** is then communicated to the controlling device **20**. The controlling device **20** is preferably capable of controlling the heating/cooling device **10** in dependence on the value of the parameter or parameters received from the input device wherein the parameter may be one as outlined above, e.g. under (i) through (viii). For example, if the operator operating the medical device enters into the input device the mass of the implant which is to be freed from the bone of a patient to which the implant adheres to then the controlling device **20** can preferably compute or look up from a database (such as a database of the invention) how much heating energy and/or cooling agent is required to heat and/or cool the implant. The same can be done if information is relayed from the input device **30** to the controlling device **20** concerning the type or dimension of the implant.

If an implant is to be removed from the bone of a patient, the implant will generally have a starting temperature which is close to the temperature of the surrounding bone tissue, i.e. about 37° C. If the temperature of the implant is to be increased for example from 37° to 47° C. the amount of energy required for this can easily be determined by computational prediction or preferably by conducting experimental tests with animal cadaver bone tissue which is pre-heated to 37° C. and into which an implant of the respective type has been inserted. Based on these tests, lookup tables can be generated for different types of implants. This table or these tables can be incorporated into the medical device of the invention allowing the controlling device to access these tables to controllably heat the implant to a desired temperature.

In one preferred embodiment, the controlling device of the medical device according to the invention controls the amount of heating energy and/or cooling agent delivered to the implant by limiting the amount of heating energy and/or cooling agent delivered to the implant in correlation to the parameters received by the input device (D). In a preferred embodiment, the input device (D) is capable of receiving the producer's name of the implant and/or the brand name of the implant plus preferably its dimensions and/or the type of coupling device being used and deriving from this information the parameter required for controlling the heating and/or cooling of the implant.

In a particularly preferred embodiment, the medical device according to the invention further comprises a thermal sensor that is capable of determining the temperature of the implant. In one embodiment the medical device is constructed such that the thermal sensor information can be received by said controlling device. This permits the controlling device to stop producing the heating energy and/or cooling agent if the desired implant temperature is reached. It is noted in this context that the selective destruction of cells located in the bone matrix can be achieved by heating or by cooling. A heat treatment of osteoclasts, osteoblasts and other cells embedded in the bone matrix of at least 42° C., preferably at least 50° C. and more preferably at least 80° C. for some time will result in the death of these cells. The same effect is achievable when cooling the cells to a temperature in which water crystals disrupt the cell membranes of these cells. This typically occurs at a temperature of below 1° C. But also a temperature of at or below 10° C. can be used to kill the cells since below this temperature also the cell's microtubule will depolymerize resulting in apoptosis of the cells. Since this biologically driven cell death is slower, the cells are preferably killed by ultra-low temperatures of at or below −30° C. which is particularly effective in rupturing the cells by ice formation.

It may be preferable to first heat the implant and then cool the implant to accelerate implant cooling and to prevent a too long heating duration. This may be advantageous for example when larger implants are used and preferably when the mass of the implant is greater than about 1 gram. Thus, in a further preferred embodiment of the medical device according to the invention said controlling device (B) is built such that it controls the device (A) such that said implant is first heated and then cooled or first cooled then heated. If the implant is first heated and then cooled a preferred medical device of the invention is built such that the implant is cooled to 40° C. or a temperature below this after the heating step.

In one embodiment of the medical device according to the invention which is also preferred, the device (A) for heating the implant directly or indirectly delivers heating energy to the implant in the form of

(i) mechanical energy (such as heating by friction)

(ii) radiation energy (e.g. heating be irradiation),

(iii) electrical energy (e.g. heating by resistance) and/or

(iv) thermal energy (e.g. direct heating).

In other words, the device (A) for heating the implant directly or indirectly delivers heating energy to the implant in the form of

(i) mechanical energy (such as heating by friction), and/or

(ii) radiation energy (e.g. heating be irradiation), and/or

(iii) electrical energy (e.g. heating by resistance) and/or

(iv) thermal energy (e.g. direct heating).

Preferably the device (A) is selected from the group consisting of moving dental bur, an ultrasonic device, an induction heating device, high frequency electric current, an emitter of laser light (or any other electromagnetic radiation), a microwave emitter, an electric heater, a heat exchanger, an infrared heater and hot air fan.

Preferably, the device (A) for cooling the implant is capable of delivering to the implant a cooling agent selected from the group consisting of air, liquefied gas, solidified gas and liquid, wherein said cooling device is preferably further capable of cooling said cooling agent to a temperature of below 20° C. and more preferably of below 10° C. and most preferably of below 0° C. In a preferred embodiment the device (A) releases an amount of cold gas or cold liquid of between 0,1 microliter and 1 milliliters and preferably between 0,5 microliter and 200 microliters. In a preferred embodiment of the medical device of the invention the medical device comprises water or isotonic saline (solution) as cooling agent.

In one embodiment of the medical device according to the invention the radiation energy is selected from ultrasonic waves, microwaves, laser light, UV light, infrared light and radio waves. Preferably, said device for heating is capable of heating the implant to a temperature of at least 42° C. and wherein the cooling device is capable of cooling the implant to a temperature of below 40° C. and more preferably of below 20° C. and even more preferably of below 10° C. and most preferably of below 0° C.

In one embodiment which is also preferred the device (A) is located within device (C). Such an embodiment could be

made for example by placing the device (A) (for example an electrical heating element) within said coupling device of the invention.

In a further preferred embodiment of the medical device of the invention the device comprises also a display to read the data entered via the input device and/or the data for heating or cooling suggested by the controlling device (e.g. target temperature of implant after heating and/or cooling).

In a further aspect the invention provides an implant for insertion into a bone of a patient, designed such that the implant is controllably heatable or coolable. An advantage of the implant according to the invention is that it is more controllably heatable or coolable than conventional implants, which are not designed according to this invention. The more controlled heating and/or cooling is possible since preferably, the implant comprises a cavity adapted to accommodate a temperature sensor, a heating device and/or a cooling device. Preferably, said cavity is or comprises a channel that has a diameter of maximally 80% of the implants diameter and wherein the diameter of the channel is at least 0.01 mm and preferably at least 0.05 mm and more preferably at least 0.1 mm and most preferably at least 0.5 mm. In another embodiment, said cavity is or comprises a channel that has a diameter of maximally 20% of the implants diameter and wherein the diameter of the channel is at least 0.01 mm and preferably at least 0.05 mm and more preferably at least 0.1 mm and most preferably at least 0.5 mm. In yet another preferred embodiment, said cavity is or comprises a channel that has a diameter of maximally 10% of the implants diameter and wherein the diameter of the channel is at least 0.01 mm and preferably at least 0.05 mm and more preferably at least 0.1 mm and most preferably at least 0.5 mm. In one preferred embodiment of the implant of the invention, the implant comprises multiple cavities. In one preferred embodiment of the implant of the invention, the cavity of the implant is conical and/or tapered and in a more preferred embodiment, the cavity is such that the diameter of the cavity at the proximal end of the implant is smaller than the diameter of the cavity at the distal end of the implant.

In one embodiment, the said cavity is or comprises a channel extending along the longitudinal axis of the implant over a distance of up to 90% and more preferably up to 95% at most preferably up to 98% of the length of the implant. In one embodiment, the said cavity is or comprises a channel extending along the longitudinal axis of the implant over a distance of at least 90% and more preferably at least 95% at most preferably over at least 98% of the length of the implant. The cavity can also extend all the way through the implant from the proximal to the distal side opening up to the outside at both ends.

In a preferred embodiment of the implant according to the invention, the implant is a dental implant for insertion into the mandibular or maxillary bone of a patient. Prior art dental implants are well known in the prior art and two examples are depicted in FIG. **1**A and FIG. **2**. If the implant of the invention is a dental implant it typically has an indentation (see e.g. FIG. **2** reference number (**4**)) suitable to insert a protrusion (see e.g. FIG. **2** reference number (**5**)) of an abutment as is well known in the art. What sets the implant of the invention apart from prior art implants is that it is controllably heatable or coolable in a more homogenous way. If the implant of the invention is a dental implant then preferred examples thereof are shown in FIGS. **1**B, **1**C (reference letters (B)-(C)) and in FIG. **3**. In these examples, the implants comprise a cavity adapted to accommodate (i) a temperature sensor, (ii) a heating device, (iii) a cooling device and/or (iv) a coupling device capable of heating and/or cooling the implant. In one embodiment the heating/cooling device or a coupling device of the invention as defined herein is inserted into the implant while the temperature sensor contacts the implant at a location other than said cavity.

In one preferred embodiment of the implant of the invention said cavity is suitable to permit a homogenous heating and/or cooling of the implant. By homogenous heating and/or cooling of the implant it is meant that the temperature of the implant can be raised and lowered such that the same temperature is reached in all targeted regions of the implant after approximately the same time of heating and/or cooling. Preferably, by homogenous heating and/or cooling of the implant according to this invention it is meant that during heating and/or cooling of the implant the temperature of the implant can be raised and lowered such that over the entire course of heating and/or cooling no part of the implant has a temperature which deviates more than 50%, more than 30%, more than 20% or most preferably more than 10% from the temperature of another part of the implant.

In one preferred embodiment of the dental implant the length of the implant is between 5 to 16 mm and the diameter is between 2 and 9 mm.

In one embodiment, said cavity of the implant of the invention opens only to the coronal surface of the implant. In one embodiment the implant according to the invention comprises a temperature sensor, a heating device, a cooling device and/or a coupling device as defined herein. In one embodiment the cavity is conically shaped such that it can receive for example a dental drill bit which has the same conical form and is capable of heating the implant by heat developed by friction between the cavity and the drill bit once the drill bit is turning. In such an embodiment however it is critical to control the temperature of the implant to avoid uncontrolled excessive heating which would result in excessive damage to the bone tissue. In such an embodiment the heating can be controlled for example by monitoring the temperature of the implant or on the drill bit. To be able to monitor the temperature of the implant the implant can comprise as outlined above within its cavity or also on its outside a temperature sensor.

In one embodiment, said cavity of the implant of the invention comprises an internal cavity with two different threaded sections, where one of the sections is capable of receiving a fixation screw capable of fixing the abutment to the implant (see for example FIG. **1**A (reference letter (A)) and a further threaded section which is suitable for receiving a coupling device of the invention. In another preferred embodiment the implant of the invention is a one-piece dental implant wherein the abutment is integrated in the implant. In embodiments where the implant of the invention is a one-piece dental implant it is further preferred that said one-piece dental implant comprises a cavity as described further herein. Preferably said cavity is or comprises a channel extending along the longitudinal axis of the one-piece implant over a distance of up to 90% and more preferably up to 95% at most preferably up to 98% of the length of said implant. In one embodiment, said cavity is or comprises a channel extending along the longitudinal axis of the implant over a distance of at least 90% and more preferably at least 95% at most preferably over at least 98% of the length of said implant. The cavity can also extend all the way through the one-piece implant from the proximal to the distal side opening up to the outside at both ends.

The diameter of said channel is preferably maximally 20% of the implants diameter wherein the diameter of the

channel is at least 0.01 mm and preferably at least 0.05 mm and more preferably at least 0.1 mm and most preferably at least 0.5 mm. In yet another preferred embodiment, said cavity is or comprises a channel that has a diameter of maximally 10% of the implants diameter and wherein the diameter of the channel is at least 0.01 mm and preferably at least 0.05 mm and more preferably at least 0.1 mm and most preferably at least 0.5 mm. In one preferred embodiment of the one-piece implant of the invention, the implant comprises multiple cavities or only a single cavity. In one preferred embodiment of the implant of the invention, the cavity of the implant is conical and/or tapered and in a more preferred embodiment, the cavity is such that the diameter of the cavity at the proximal end of the implant is smaller than the diameter of the cavity at the distal end of the implant.

Regardless whether the implant is a dental implant or not, it is preferred that in one embodiment of the implant of the invention said cavity is filled by a solid material which can be removed from the implant prior to inserting said temperature sensor, heating device, cooling device and/or a coupling device. The material is preferably suitable to seal the cavity to prevent ingrowth of bacteria. Preferably the material is flexible and/or brittle which will allow an easy removal. In one embodiment the material is rubber or a thermoplastic polymer.

In one embodiment at least part of the material present in said cavity has the shape of a screw. This permits the material to be removed by screwing the material out of the cavity prior to insertion of the coupling device of the invention.

In one preferred embodiment of the implant of the invention said cavity overlaps with the centre of the volume of the implant.

A further aspect of the invention relates to a method of removing an implant from a bone of a patient comprising the step of heating and/or cooling the implant, wherein at least part of the implant is osseointegrated in the bone.

In this method the implant is preferably heated to a temperature of between 40° C. and 500° C., more preferable between 42° C. and 90° C. and most preferable between 50° C. and 80° C. It is preferred that the implant is actively cooled after the implant is heated if the implant is heated to a temperature of above 80° C. Thus, the method allows the implant to be quickly heated to a temperature of above 80° C. and then immediately cooled back down e.g. to a temperature of below 40° C. Using high temperatures for a short time by active cooling immediately thereafter will coagulate only a thin layer of bone tissue and is expected to reduce damage to the bone. In one preferred embodiment of the method of the invention the implant is cooled to a temperature of below 37° C., preferably below 20° C. and most preferably below 3° C. If the method does not include a heating step then it is preferred that the implant is cooled to a temperature of below 10° C. and preferably of below 0° C. and most preferably to a temperature of below −30° C. A controlled cooling can be achieved by contacting the implant with a measured predetermined amount of cooling agent. For example, the implant can be cooled by contacting it with between 0.001 vol % and 10000 vol % of a cooling agent based on the volume of the implant. If the implant is heated in the method of the invention it is preferred that the implant is heated for a time period of between 1/10 of a second and 3 days. If the implant is heated for up to 5 minutes it is preferred that the temperature of the implant is preferably kept above 50° C. If the implant is heated for longer than 5 minutes it is preferred that the temperature of the implant is preferably kept between 42° C. and 50° C. In one preferred method of the invention the implant is heated to between 70° C. and 90° C. for 1-5 minutes. In another preferred method the implant is heated to above 100° C. for under 30 seconds and actively cooled to under 40° C. directly thereafter.

In a further preferred embodiment of the method of the invention, the implant used in the method is an implant of the invention as defined herein.

A database is a collection of related data that is generally stored on a non-volatile memory medium. Data in the database is commonly organized in a two-dimensional row and column form called a table. A database typically includes multiple tables.

A further aspect of the invention relates to a computer readable storage medium having recorded thereon a database structure where the database structure comprises data representing

(a) implant information such as information that defines the type, dimensions, mass, surface structure and/or material of the implant and/or that defines the cavity within the implant; and/or
(b) information defining the properties of the coupling device such as its thermal conductivity; and/or
(c) information defining the part or surface of the implant which is expected to be osseointegrated; and/or
(d) information defining the type of heating energy and/or cooling agent to be used and optionally the amount of heating energy and/or cooling agent necessary to achieve osseodisintegration of an implant as defined in (a) preferably with minimal trauma to the surrounding tissues; and/or
(e) information for controlling the device (A) to heat or cool the implant such to achieve osseodisintegration of bone material contacting the implant or located within a distance of less than a predefined distance from the surface of said implant; and/or
(f) any parameter that the input device of the invention is capable of receiving as outlined above under (i) through (viii); and/or
(g) information defining the temperature, temperature curve, heating time, cooling time, frequency of heating and/or cooling which are suitable to effect osseodisintegration of an implant as defined in (a); and/or
(h) information defining the bone density of the bone contacting or surrounding the implant; and/or
(i) information defining the date on which the implant was implanted into the patient; and/or
(j) information defining the age of the patient.

A preferred embodiment of the above aspect of the invention relates to a computer readable storage medium having recorded thereon a database structure where the database structure comprises data representing

(a) implant information such as information that defines the type, dimensions, mass and/or material of the implant and/or that defines the cavity within the implant;
(b) information defining the properties of the coupling device such as its thermal conductivity
(c) information defining the part or surface of the implant which is expected to be osseointegrated
(d) information defining the type of heating energy and/or cooling agent to be used and optionally the amount of heating energy and/or cooling agent necessary to achieve osseodisintegration of an implant as defined in (a) preferably with minimal trauma to the surrounding tissues;
(e) information for controlling the device (A) to heat or cool the implant such to achieve osseodisintegration of

bone material contacting the implant or located within a distance of less than a predefined distance from the surface of said implant; and/or

(f) any parameter that the input device of the invention is capable of receiving as outlined above under (i) through (viii).

As mentioned above, such database structure can be produced based on experimental data obtained for example from tests with bone tissue from dead animals, whereby it is determined what amount of heating energy or cooling agent is required for a specific implant and for a specific degree of osseodisintegration in order that the bone material contacting the implant or located within a distance of less than a predefined distance from the surface of said implant is selectively degraded. Preferably said computer readable storage medium also has recorded thereon a software suitable to operate the device of the invention.

As a further aspect the invention also provides a coupling device as defined herein.

Various modifications and variations of the invention will be apparent to those skilled in the art without departing from the scope of the invention. Although the invention has been described in connection with specific preferred embodiments, it should be understood that the invention as claimed should not be unduly limited to such specific embodiments. Indeed, various modifications of the described modes for carrying out the invention which are obvious to those skilled in the relevant fields are intended to be covered by the present invention.

The following examples depicted in the drawings are merely illustrative of the present invention and should not be construed to limit the scope of the invention as indicated by the appended claims in any way.

BRIEF DESCRIPTION OF THE DRAWINGS

FIGS. **1**A-C: Shown are different two-part devices comprising a dental implant (A, B or C). All parts are non-limiting examples, are illustrated as cross-sections and not drawn to scale. The dental implants shown are implantable into the mandibular or maxillary bone of a patient. Also shown in FIG. **1**A is a conventional abutment part (D) for the two-part device. The proximal end of the abutment part can be inserted into the respective dental implant as shown, whereas the distal end of the abutment will face the oral cavity and a crown or a superstructure may be placed on this distal end of the abutment. As shown in FIGS. **1**B and **1**C, dental implants (B) and (C) are according to the invention and comprise a cavity adapted to accommodate (i) a temperature sensor, (ii) a heating device, (iii) a cooling device and/or (iv) a coupling device capable of heating and/or cooling the implant (said cavity is depicted as hatched area). In the non-limiting examples (B) and (C) the cavity is a channel extending along the longitudinal axis of the implant. The heating and cooling can be achieved for example by using a coupling device as illustrated in FIGS. **1**A-E (reference letters (D), (E), (F), (G) or (H)) which has at least part of the shape of a dental implant abutment which allows said coupling device to be releasably connectable to said dental implant. As shown in FIG. **1**, the coupling device (D), (E), (F), (G), or (H) connects a heating/cooling device **10** to the implant (B) and (C) such that a channel **12** of implant (B) and (C) receives heating energy or a cooling agent delivered from the heating/cooling device **10**. The heat and/or cold transfer is achievable using a medical device according to the invention comprising a coupling device as exemplified.

FIG. **2**: Shows a different conventional type of two-part device comprising a dental implant (**1**) and a respective abutment part (**2**).

FIG. **3**: Exemplary illustration of a dental implant according to the invention comprising a cavity (**6**) adapted to accommodate (i) a temperature sensor, (ii) a heating device, (iii) a cooling device and/or (iv) a coupling device capable of heating and/or cooling the implant.

FIGS. **4**A-D: Shown are different types of preferred coupling devices according to the invention that can be used with the implant shown in FIG. **3**. The coupling devices are capable of releasably connecting a device capable of heating and/or cooling the implant, to an implant such as the implant shown in FIG. **3**.

When an implant to be removed is located in the patient and is osseointegrated in the bone, the dentist can chose from different types of coupling devices such as shown in FIGS. **1**A-E (reference letters (D)-(H)) or FIGS. **4**A D) to heat and/or cool the implant. If the implant to be removed is a conventional implant (like implant A shown in FIG. **1**A, i.e. one not modified according to this invention), he may have to choose a coupling device in the shape of coupling device (D) or (H) shown in FIGS. **1**A, **1**B, because E-G coupling devices (E) and (G) shown in FIGS. **1**B, **1**D will not fit. For the conventional implant shown in FIG. **2** he preferably uses a coupling device as shown in FIG. **4**A, because coupling devices shown in FIGS. **4**B-D will not fit. A controlled thermal influencing of conventional implants (like implant (A) shown in FIGS. **1**A and **2**) to reach intended osseodisintegration will be possible using the medical device of the invention in connection with e.g. a coupling device (D), (H) and (a) as shown in FIGS. **1**A, **1**E and FIG. **4**A, respectively. However, the thermal heating and/or cooling of the implant can be controlled over time even more precisely if the heating and/or cooling of the implant is more homogenous. This is possible when using implants modified according to this invention (such as shown in FIGS. **1**B, **1**C and **3**.

If the implant to be removed is an implant modified according to this invention (like implants B and C shown in FIGS. **1**B, **1**C, he may choose for example a coupling device in the shape of coupling devices (D)-(H) shown in FIGS. **1**A-E, and preferably a coupling device according to any of the coupling devices (E)-(G) shown in FIGS. **1**B-D, depending on the expected region of osseointegration on the implant's surface. If the complete implant is osseointegrated, he may choose coupling device F shown in FIG. **1**C, to thermally influence the whole body of the implant. If only the proximal part of the implant is expected to be osseointegrated, as is clinically frequently the case with periimplantitis, he may choose type G shown in FIG. **1**D, to influence especially the proximal part of the implant. Therefore, in the device G the distal part connecting to the distal walls of the implant should have little surface connecting directly to the implant and be of low thermal conductivity.

If the implant to be removed is an implant modified according to this invention (like FIG. **3**), the treating medical expert may choose a coupling device in the shape of coupling device (b) shown in FIG. **4**B, coupling device (c) shown in FIG. **4**C, or coupling device (d) shown in FIG. **4**D, depending on the expected region of osseointegration on the implant's surface. If the complete implant is osseointegrated, he may choose coupling device c, to thermally influence the whole body of the implant. If only the proximal part of the implant is expected to be osseointegrated, as is clinically often the case with periimplantitis, he may choose coupling device (d) shown in FIG. **4**D, to selectively influ-

ence especially the proximal part of the implant. Therefore, the coupling device (d) shown in FIG. **4**D is designed such that the most distal part of the coupling device has a reduced contact with the distal walls of the implant when the coupling device is inserted into the implant, resulting in low thermal conductivity between the distal region of the implant and the distal part of the coupling device.

Many different shapes and designs are possible for the implants of the invention, for the respective coupling devices and the coupling devices for conventional implants

LISTING OF REFERENCE NUMERALS

(**1**) Dental implant
(**2**) Abutment
(**3**) Distal part
(**4**) Indentation
(**5**) Protrusion
(**6**) Cavity adapted to accommodate a temperature sensor, a heating device and/or a cooling device

The invention claimed is:

1. A medical device for releasing an implant from a bone of a patient to which the implant adheres to, the medical device comprising:

a heating/cooling device configured to produce or release heating energy and/or a cooling agent to heat and/or cool the implant;

a controlling device configured to control the heating/cooling device, wherein the controlling device comprises a database storing information and controls the heating/cooling device based on the information stored in the database;

wherein the database comprises:

implant information; and

information defining a temperature, a temperature curve, a heating time, a cooling time, and a frequency of heating and/or cooling suitable to effect osseodisintegration of the implant based on the implant information;

a coupling device configured to releasably connect to the implant while the implant adheres to the bone of the patient, wherein the coupling device is configured to transfer the heating energy and/or the cooling agent produced or released by the heating/cooling device to the implant; and

an input device operably linked with the controlling device, wherein the input device is configured to receive a parameter from an operator operating the medical device, wherein the parameter:

(i) defines a type, dimension, mass, surface structure and/or material of said implant; and/or

(ii) defines an expected amount of osseointegrated surface on the implant; and/or

(iii) defines an amount and/or duration of the heating and/or cooling; and/or

(iv) defines an expected position or region of the osseointegrated surface on the implant; and/or

(v) is selected from the group consisting of

(a) information defining a type, dimensions, mass, surface structure and/or material of the implant or a cavity geometry within the implant;

(b) information defining a form of the coupling device or information defining a thermal conductivity of the coupling device;

(c) information defining a surface of the implant which is osseointegrated;

(d) information defining a type of heating energy and/or cooling agent to be used or information defining an amount of heating energy and/or cooling agent necessary to reach osseodisintegration of an implant as defined in (a); and

(e) information for controlling the heating/cooling device to heat or cool the implant to achieve osseodisintegration of bone material contacting the implant or located within a distance of less than a predefined distance from the surface of said implant;

and/or

(vi) defines bone density of the bone contacting or surrounding the implant; and/or

(vii) defines a date on which the implant was implanted into the patient; and/or

(viii) defines an age of the patient.

2. The medical device according to claim **1**, wherein the implant is a dental implant which is at least partially osseointegrated in the mandibular or maxillary bone of a patient.

3. The medical device according to claim **2**, wherein said coupling device has at least part of the shape of a dental implant abutment which allows said coupling device to be releasably connectable to said dental implant.

4. The medical device according to claim **1**, wherein said coupling device has the shape of a ring which allows said coupling device to be releasably connectable to said implant.

5. The medical device according to claim **1**, wherein said coupling device is made from a material that has a thermal conductivity of at least 0.5 W/(m·K).

6. The medical device according to claim **1**, wherein said controlling device controls the amount of heating energy and/or cooling agent delivered to the implant to an amount that is sufficient to achieve osseodisintegration of bone material which contacts the implant.

7. The medical device according to claim **1**,

wherein the implant information defines a type, dimensions, a mass, a surface structure and/or a material of the implant or a cavity within the implant, wherein the database further comprises:

information defining the properties of the coupling device such as form, surface connection with the implant, and/or thermal conductivity;

information defining the surface of the implant which is osseointegrated; and/or

information defining the type of heating energy and/or cooling agent to be used and the amount of heating energy and/or cooling agent necessary to reach osseodisintegration of the implant based on implant information; and/or

information for controlling the heating/cooling device to heat or cool the implant such to achieve osseodisintegration of bone material contacting the implant or located within a distance of less than a predefined distance from the surface of said implant; and/or

information defining the bone density of the bone contacting or surrounding the implant; and/or

information defining the date on which the implant was implanted into the patient; and/or

information defining the age of the patient.

8. The medical device according to claim **1**, wherein said controlling device controls the amount of heating energy and/or cooling agent delivered to the implant by limiting the amount of heating energy and/or cooling agent delivered to the implant in correlation to the parameter or parameters received by the input device.

9. The medical device according to claim 1, wherein said controlling device controls the heating/cooling device such that said implant is first heated and then cooled or first cooled then heated.

10. The medical device according to claim 1, wherein the heating/cooling device for heating the implant directly or indirectly delivers heating energy to the implant in the form of

(i) mechanical energy, and/or
(ii) radiation energy, and/or
(iii) electrical energy and/or
(iv) thermal energy.

11. The medical device according to claim 1, wherein the heating/cooling device for cooling the implant is configured to deliver to the implant said cooling agent selected from the group consisting of air, liquefied gas, solidified gas and liquid.

12. The medical device according to claim 1, wherein heating/cooling device is located within the coupling device.

13. The medical device of claim 6, wherein the osseodisintegration is limited to the bone material located within a distance of about 0.1 mm to 3 mm from the surface of the implant.

14. The medical device of claim 11, wherein said cooling device is configured to cool said cooling agent to a temperature of below 20° C.

* * * * *
